# Tailored Catalysts Based on Polymers of Intrinsic Microporosity for Asymmetric Aza-Henry Reaction of Pyrazolinone Ketimines in Batch and Flow

**DOI:** 10.1021/acsapm.5c02439

**Published:** 2025-10-06

**Authors:** Rodrigo Sánchez-Molpeceres, Paolo Zhan, Laura Martín, Alicia Maestro, Jesús A. Miguel, Bibiana Comesaña-Gándara, José M. Andrés

**Affiliations:** † GIR SintACat, IU CINQUIMA/Química Orgánica, Facultad de Ciencias, 16782 Universidad de Valladolid, Paseo Belén 7, 47011 Valladolid, Spain; ‡ IU CINQUIMA/Química Inorgánica, Facultad de Ciencias, Universidad de Valladolid, Paseo Belén 7, 47011 Valladolid, Spain

**Keywords:** polymers of intrinsic microporosity, heterogeneous catalyst, quinine-derived thiourea, asymmetric aza-Henry, pyrazolinone ketimines, continuous flow

## Abstract

We report the design and application of heterogeneous organocatalysts based on polymers of intrinsic microporosity (PIMs) for the asymmetric aza-Henry reaction of *N*-Boc-protected pyrazolinone ketimines with nitromethane. Two copolymers (PIM-10 and PIM-20) incorporating a flexible isatin-derived monomer were synthesized and postfunctionalized with a quinine-derived thiourea. The resulting materials, PIM-10-TU and PIM-20-TU, exhibited high thermal stability, tailored porosity, and effective enantioselective catalytic performance in batch- and continuous-flow conditions. PIM-10-TU showed superior activity and recyclability, achieving full conversion in 2–4 h and affording β-nitroamine derivatives in up to 87% yield and 85:15 er. Flow experiments enabled gram-scale synthesis with short residence times and a sustained efficiency. The synthetic utility of the chiral aminopyrazolones was demonstrated via derivatization to ureas and thioureas without erosion of the enantiopurity. This study highlights the potential of PIM-supported organocatalysts as robust and recyclable platforms for asymmetric synthesis under sustainable conditions.

## Graphical abstract

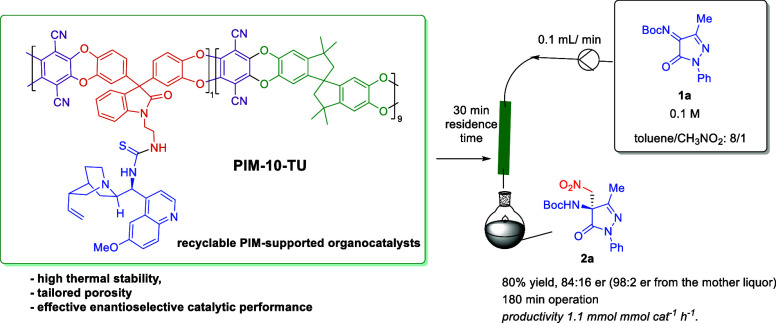

## 1 Introduction

The chemical industry increasingly demands efficient, ecofriendly, and stable catalysts for organic synthesis. In asymmetric catalysis, reducing metal leaching is vital, particularly in pharmaceutical applications. One effective solution involves the use of heterogeneous materials, where catalysts are supported on or embedded in an insoluble material to improve recovery.^1^ The growing need for enantiomerically pure compounds in fields such as agrochemicals and pharmaceuticals has driven the development of efficient synthesis strategies. Organocatalysis offers a metal-free alternative, but separating these catalysts from products can be challenging.^2^ In this context, cinchona-based heterogeneous catalysts offer practical advantages in flow chemistry, including easier handling, improved stability, and enhanced recovery and recyclability.^3,4^

Polymers of intrinsic microporosity (PIMs) are a class of materials that combine the microporosity of solids with the solubility and processability of glassy polymers.^5^ The first example, PIM-1, was synthesized in 2004 through nucleophilic condensation between a spirobisindane-based bis­(catechol) and planar aromatic *ortho*-dihalo monomers.^6^ Thanks to their solubility in common organic solvents, PIMs are easily processable and widely used in gas separation membranes.^7–9^ Their high porosity, simple synthesis, thermal and chemical stability, and good processability make them promising candidates for various catalytic applications.^10^

Despite this, their use as supports for asymmetric catalysis remains limited. A few studies report the postfunctionalization of PIM-1 and PIM-1n with bifunctional thioureas and squaramides, which were employed as recyclable organocatalysts in enantioselective nitro-Michael reactions^11^ and α-amination of 3-aryl oxindoles under batch and flow conditions,^12^ highlighting the role of the microporous environment in asymmetric induction. Recently, our group developed a cost-effective method for the synthesis of oxindole-containing bifunctional thioureas, which were subsequently immobilized on a linear organic polymer. The resulting materials serve as efficient heterogeneous catalysts for the α-amination of 4-substituted pyrazolones under flow conditions.^13^ These materials, together with porous organic polymers reported by Rico-Martínez et al., have also shown potential in carbon capture, gas separation, and as supports for heterogeneous organometallic catalysis.^14^

The enantioselective aza-Henry (or nitro-Mannich) reaction, in which a nitroalkane reacts with an imine to form a β-nitroamine, is a versatile tool for synthesizing biologically active compounds and their intermediates.^15–17^ Various transition metal complexes and organocatalysts have been employed to promote this reaction enantioselectively with different types of imines, often achieving high levels of asymmetric induction.^18–22^

Pyrazoles and pyrazolones are important five-membered aza-heterocycles commonly found in pharmaceutical drugs,^23^ though rarely in natural products. Chiral α-tertiary amines are also key structural motifs in many natural products, bioactive molecules, pharmaceuticals, and agrochemicals.^24,25^ Combining these two elements into hybrid molecules could offer promising avenues for drug discovery, yet few examples exist despite their potential.^26,27^ Recently, significant efforts have focused on developing organocatalytic enantioselective methods to synthesize pyrazolones bearing a tetrasubstituted stereocenter with an amino group at the C4-position.^28–30^ Two main approaches have been explored: electrophilic amination of 4-monosubstituted pyrazolones^31^ and enantioselective nucleophilic addition to pyrazole-4,5-dione ketimines, as developed by Enders. While several organocatalytic transformations such as Strecker,^32^ aza-Friedel–Crafts,^33,34^ and Mannich reactions^35–39^ have been reported using these ketimines, no examples of enantioselective aza-Henry reactions involving *N*-Boc-protected pyrazolinone ketimines have been described to date (Scheme 1).

**Scheme 1:**
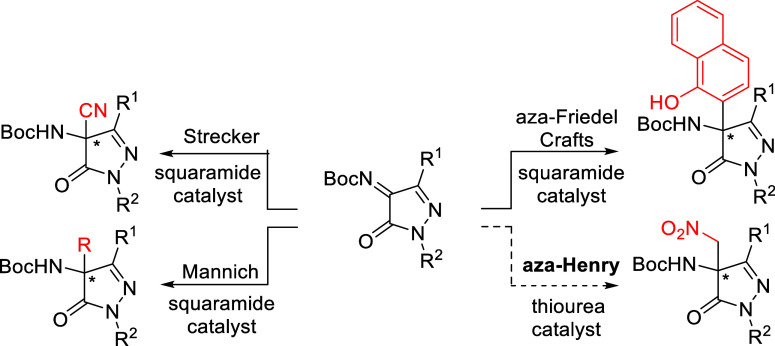
Background of Catalytic Asymmetric Reaction of Pyrazolinone Ketimines

Herein, we present our results on the asymmetric aza-Henry reaction of *N*-Boc-protected pyrazolinone ketimines with nitromethane, catalyzed by a bifunctional quinine-derived thiourea immobilized on a PIM-based copolymer. To improve swelling in organic solvents and create larger pores for accommodating bulky substrates, we used a more flexible isatin-based monomer in the PIM synthesis. These heterogeneous catalysts, applied in both batch and continuous flow systems, benefit from a high surface area and excellent thermal and chemical stability. Their performance was evaluated in the enantioselective synthesis of enantioenriched 4-aminopyrazolone derivatives with potential pharmacological relevance.

## 2 Experimental Part

### 2.1 Materials

Commercially available organic and inorganic compounds were used without further purification. Solvents were dried and stored over microwave-activated 4 Å molecular sieves. Compounds such as 9-amino (9-deoxy)­epi-quinine (QN-NH_2_),^40^ (8a,9*S*)-9-isothiocyanato-6′-methoxycinchonan (QN-NCS),^41^ *N*-Boc pyrazolinone ketimines,^35^ model catalysts C1–C9,^13^ *N*-(2-bromoethyl)-2-phtalimide,^13^ and *N*-alkyl isatin derivative^42^ were prepared as previously described. Racemic reference samples were prepared using an achiral bifunctional thiourea derived from *N* ^1^,*N* ^1^-dimethylethane-1,2-diamine^43^ as a catalyst in the same conditions as the asymmetric reaction.

### 2.2 Synthesis of Polymeric Materials

#### 2.2.1 PIM-10

The three monomers, 3,3-diaryl oxindole (M1, 0.30 g, 0.6 mmol, 1.0 equiv), 5,5′,6,6′-tetrahydroxy-3,3,3′,3′-tetramethylspirobisindane (TTBSI, 1.75 g, 5.1 mmol, 9.0 equiv), and 2,3,5,6-tetrafluoroterephthalonitrile (TFTPN, 1.14 g, 5.7 mmol, 10.0 equiv), were combined with an excess of potassium carbonate (K_2_CO_3_, 2.38 g, 1.8 mmol, 30.0 equiv) in anhydrous *N*,*N*-dimethylformamide (DMF, 38 mL), and the mixture was stirred at 65 °C for 3 days. Upon completion of the reaction, the crude product precipitated into cold water (120 mL) and stirred for 20 min. The resulting solid was filtered and washed with water and methanol (3 × 20 mL), and dried. The crude material was then redissolved overnight in chloroform (CHCl_3_, 20 mg/mL) and reprecipitated in methanol (100 mL). After filtration, it was washed again with methanol and dried at 180 °C under a dynamic vacuum (60 mbar) for 24 h. The material PIM-10 was obtained as a yellow powder in nearly quantitative yield (2.60 g, 93%). ^1^H nuclear magnetic resonance (NMR) (500 MHz, CDCl_3_, δ) 7.88–7.54 (m, 4H), 7.25–6.89 (m, 4H), 6.82 (br, 20H), 6.43 (br, 22H), 4.26–3.97 (m, 4H), 2.70–2.00 (br, 36H), 1.76–0.93 (m, 108H) ppm. FTIR (ATR, cm^–1^): 2955, 2864, 2240, 1720, 1607, 1446, 1263, 1108, 1009, 874. Inherent viscosity (CHCl_3_, 0.5 g/dL): 0.392 dL/g. *M*_n_ = 13,717 and *M*_w_ = 16,634 by gel permeation chromatography (GPC), calculated against polystyrene standards.

#### 2.2.2 PIM-20

The reaction was carried out starting from M1 (160 mg, 0.3 mmol, 2.0 equiv), TTBSI (0.41 g, 1.2 mmol, 8.0 equiv), and TFTPN (0.30 g, 1.5 mmol, 10.0 equiv) in the presence of an excess of K_2_CO_3_ (0.63 g, 30.0 equiv) in DMF (20 mL) following the procedure described for PIM-10. The product, PIM-20, was obtained as a yellow powder (0.70 g, 91%). ^1^H NMR (500 MHz, CDCl_3_, δ) 7.79–7.59 (m, 4H), 7.20–6.94 (m, 4H), 6.81 (br, 13H), 6.43 (br, 9H), 4.15 (br, 2H), 4.05 (br, 2H), 2.34 (br, 8H), 2.18 (br, 8H), 1.32 (br, 48H) ppm. FTIR (ATR, cm^–1^): 2955, 2864, 2240, 1718, 1607, 1448, 1264, 1108, 1011, 911. Inherent viscosity (CHCl_3_, 0.5 g/dL): 0.491 dL/g. *M*_n_ = 38,783 and *M*_w_ = 75,899 by GPC, calculated against polystyrene standards.

#### 2.2.3 Amine-PIM-10

Copolymer PIM-10 (2.30 g) was suspended in methanol (70 mL), followed by the addition of an excess of hydrazine hydrate (2.3 mL) under stirring. The reaction was carried out at 40 °C overnight. The reaction mixture was filtered, washed with methanol (3 × 20 mL), and dried at 60 °C under dynamic vacuum (60 mbar) for 24 h, affording amine-PIM-10 as a yellow powder (2.10 g, 94%). ^1^H NMR (500 MHz, CDCl_3_, δ) 6.81 (br, 32H), 6.41 (br, 14H), 3.86 (br, 2H), 3.03 (br, 2H), 2.35–2.17 (m, 36H), 1.60–1.10 (m, 110H) ppm. FTIR (ATR, cm^–1^): 2955, 2864, 2239, 1714, 1607, 1450, 1265, 1108, 1011, 875.

#### 2.2.4 Amine-PIM-20

Copolymer PIM-20 (0.50 g) was suspended in methanol (15 mL), and an excess of hydrazine hydrate (0.5 mL) was added. The mixture was stirred by following the procedure described for *amine-PIM-10*. The product, *amine*-*PIM-20*, was obtained as a yellow powder in a nearly quantitative yield (0.43 g, 90%). ^1^H NMR (500 MHz, CDCl_3_, δ) 7.10–6.15 (m, 26H), 3.87 (br, 2H), 3.06 (br, 2H), 2.46–2.10 (m, 16H), 1.77–1.09 (m, 50H) ppm. FTIR (ATR, cm^–1^): 2954, 2864, 2239, 1716, 1607, 1448, 1265, 1108, 1011, 874, 811.

#### 2.2.5 PIM-10-TU

Amine-PIM-10 polymer (1.80 g) was dissolved in chloroform (90 mL) with an excess of QN-NCS (0.60 g). The resulting solution was stirred at 50 °C for 3 days. After solvent evaporation, the resulting solid was then triturated with methanol (20 mL) and collected by filtration to recover the unreacted isocyanate. The polymer was washed with methanol (3 × 20 mL) and dried at 60 °C under dynamic vacuum (60 mbar) for 24 h, affording polymeric thiourea as a yellow powder in quantitative yield. The degree of effective functionalization (*f*) was calculated based on sulfur content determined by elemental analysis. ^1^H NMR (500 MHz, CDCl_3_, δ) 8.68 (br, 1H), 7.96 (br, 1H), 6.81 (br, 31H), 6.42 (br, 18H), 5.87–4.42 (m, 3H), 5.08 (br, 2H), 4.47–3.31 (m, 7H), 2.33 (br, 18H), 2.17 (br, 19H), 1.90–0.63 (m, 119H) ppm. ^13^C NMR CPMAS: 30 (br), 43 (br), 58 (br), 94 (br), 111 (br), 128 (br), 140 (br), 149 (br), 157 (br) ppm. FTIR (ATR, cm^–1^): 2955, 2865, 2239, 2162, 2051, 1713, 1608, 1448, 1264, 1108, 1010, 874, 753. *f* = 0.35 mmol g^–1^ (C, 68.68; H, 4.60; N, 7.57; S: 1.12).

#### 2.2.6 PIM-20-TU

Amine-PIM-20 (0.40 g) was dissolved in chloroform (20 mL) together with an excess of QN-NCS (0.10 g), following the procedure described for *PIM-10-TU.* The polymeric thiourea was obtained as a yellow powder in a quantitative yield. An effective functionalization, *f* = 0.32 mmol g^–1^, was calculated based on sulfur content determined by elemental analysis (C, 69.23; H, 4.92; N, 7.45; S: 1.01). ^1^H NMR (500 MHz, CDCl_3_, δ) 8.75 (br, 1H), 8.03 (br, 1H), 7.75–6.10 (br, 29H), 5.84–4.6 (m, 5H), 4.17–3.66 (m, 3H), 3.55–2.66 (br, 5H), 2.51–1.94 (m, 16H), 1.87–0.77 (m, 59H) ppm. ^13^C NMR CPMAS: 30 (br), 44 (br), 58 (br), 95 (br), 111 (br), 127 (br), 140 (br), 148 (br), 158 (br) ppm. FTIR (ATR, cm^–1^): 2954, 2864, 2323, 2239, 1711, 1608, 1446, 1264, 1108, 1010, 874, 753.

### 2.3 Catalytic Experiments

#### 2.3.1 General Procedure for the Stereoselective Synthesis of Aza-Henry Products Using Homogeneous Model Catalysts

To a solution of the corresponding *N*-Boc ketimine **1a**–**n** (0.1 mmol) and catalyst **C1–C9** (5 mol %) in 1 mL of the selected solvent, nitroalkane (0.6 mmol, 6 equiv) was added. The reaction mixture was stirred at room temperature until complete consumption of the starting ketimine, as monitored by ^1^H NMR. Upon completion, the solvent was evaporated under reduced pressure, and the residue was purified by flash chromatography (hexane/EtOAc, 4:1) to afford the corresponding product. The enantiomeric excess was determined by chiral-phase high-performance liquid chromatography (HPLC) analysis using hexane/isopropyl alcohol mixtures as the eluent.

#### 2.3.2 General Procedure for Heterogeneous Catalysis

To a solution of *N*-Boc ketimine **1a** (23 mg, 0.08 mmol) and the corresponding polymeric thiourea catalyst (20 mol %) in toluene (1 mL), nitromethane (12–48 equiv) was added. The reaction mixture was stirred at room temperature until complete consumption of the starting ketimine, as monitored by ^1^H NMR. After completion, the mixture was centrifuged, and the supernatant (reaction crude) was concentrated and purified following the previously described procedure to afford **2a**.

#### 2.3.3 Recycling Experiments

PIM-10-TU (20 mol %) was preswelled in a mixture of toluene (1 mL) and nitromethane (24 equiv) and stirred at room temperature for 20 min. Then, *N*-Boc ketimine **1a** (23 mg, 0.08 mmol) was added, and the reaction mixture was stirred at room temperature for 6 h. After completion, the mixture was centrifuged, and the supernatant was concentrated and purified, following the previously described procedure to afford **2a**. The polymer was washed with toluene (3 × 10 min), with centrifugation at 4500 rpm after each wash. The recovered PIM-10-TU catalyst was reused directly in subsequent cycles without prior drying.

### 2.4 Characterization Techniques

NMR spectra were recorded at the Laboratory of Instrumental Techniques (LTI), University of Valladolid (UVa), using Bruker Avance400 Ultrashield, Varian400 MR, and Varian500/54 Premium Shielded spectrometers at 25 °C. CDCl_3_ and dimethyl sulfoxide (DMSO)-*d*_6_ were employed as the solvents. Solid-state ^13^C cross-polarization magic-angle spinning NMR (CP/MAS ^13^C NMR) spectra were recorded on a Bruker Advance 500 spectrometer operating at a Larmor frequency of 125.7 MHz using a contact time of 2 ms and a delay time of 4 s. All samples were spun at 20 kHz. ESI mass spectra were acquired on an Agilent 5973 inert GC/MS system at LTI (UVa). Elemental analyses were conducted using a LECO CHNS-932 at the Elemental Analysis Center, Complutense University of Madrid. Infrared (IR) spectra were recorded on a PerkinElmer spectrum One FT-IR spectrometer and are reported as absorption frequencies. Inherent viscosities were measured at the Institute of Polymer Science and Technology of the Spanish National Research Council (ICTP-CSIC) using a Lauda iVisc device and a Ubbelohde viscometer. The viscosities of PIM-10 and PIM-20 were determined at 30 °C in CHCl_3_ at a concentration of 0.5 g/dL. Molecular weights and molecular weight distributions of PIM-10 and PIM-20 were analyzed by GPC using Styragel columns and tetrahydrofuran (THF) as the eluent with a flow rate of 1 mL/min. The measurements were carried out at the ICTP-CSIC (Technical Research Support Unit).

Thermogravimetric analyses (TGA) were performed on a TA Q-550 thermobalance (TA Instruments, Milford, USA). Dynamic ramp scans were run at 10 °C min^–1^ from 50–850 °C in the presence of a N_2_ purge gas flux. Scanning electron microscopy (SEM) images were obtained using a Quanta200FEG microscope (FEI, Hillsboro, OR) on Au-metallized samples, operated at an acceleration voltage of 15–20 kV under high vacuum, using secondary electron detection at LTI (UVa). Atomic force microscopy (AFM) measurements were performed in air at 25 °C using an MFP-3D Bio (Asylum Research, Oxford Instruments) with an AC240 NA cantilever (OPUS by μMasch) at the LTI (UVa). Topography and phase images were acquired with 512 × 512 data points at scan sizes of 2.5 × 2.5 μm^2^. Samples were prepared by drop-casting 50 μL of each polymer suspension in methanol onto freshly cleaved mica substrates, followed by solvent evaporation at room temperature. Data acquisitions and roughness analyses were performed using Asylum Research software (AR 16.33.234).

Low-temperature (77 K) N_2_ adsorption/desorption isotherms were obtained using a Micromeritic surface area analyzer. The powder samples were degassed for 12 h at 120 °C under a high vacuum prior to analysis.

Specific rotations were measured using a PerkinElmer 341 digital polarimeter with a 1 dm path length cell (5 mL capacity) and a sodium lamp as the light source. Concentrations are reported in g/100 mL. Flash chromatography was performed on silica gel (230–240 mesh). Reported chemical yields refer to pure isolated compounds. Thin-layer chromatography analysis was conducted on aluminum-backed plates coated with silica gel 60 and an F254 indicator. Spots were visualized under UV light or by staining with a phosphomolybdic acid solution, followed by heating. Melting points were determined in open capillary tubes and are uncorrected. Chiral HPLC analysis was carried out using a JASCO system equipped with a PU-2089 pump, UV-2075 UV/vis detector, and a quaternary solvent delivery system. Separations were performed on Chiralpak AD-H and Lux Amylose-1 columns (250 × 4.6 mm). Detection was monitored at 254 nm.

## 3 Results and Discussion

### 3.1 Preliminary Studies and Synthesis of Heterogeneous Catalyst for Asymmetric Aza-Henry Reaction

Initially, a series of homogeneous catalysts (**C1**–**C9**),^13,44^ based on a 3,3-diaryl-oxindole scaffold, were evaluated in the asymmetric aza-Henry reaction between the pyrazolinone-derived ketimine **1a** and nitromethane, with the aim of identifying the most efficient chiral catalyst for subsequent immobilization onto a copolymer support. The results of this study, including the evaluation of different catalysts and variations in reaction conditions such as solvent, nitromethane molar ratio, and catalyst loading, are provided in the Supporting Information (Table S1). Scheme 2 summarizes the optimized conditions for the aza-Henry reaction, which involve 5 mol % of quinine-derived thiourea catalyst **C1**, 12 equiv of nitromethane, and toluene as the solvent at room temperature. These conditions were then applied to evaluate the reaction’s scope and limitations with a variety of pyrazolinone-derived ketimines (**1a**–**n**) and nitroalkanes.

**Scheme 2:**
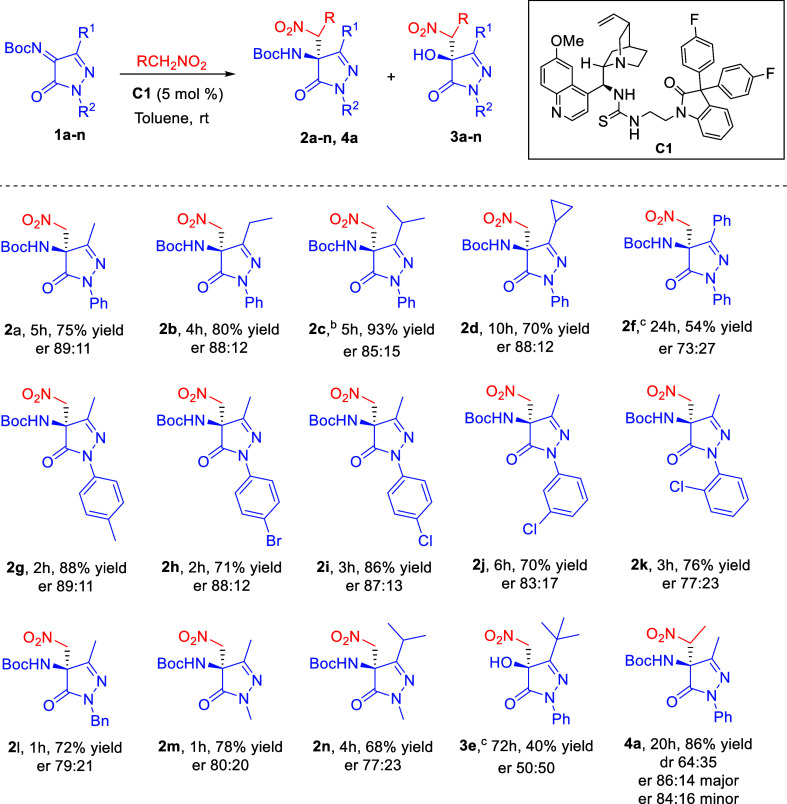
Optimized Conditions and Substrate Scope for the Aza-Henry Reaction[Fn s2fn1]

a
**1a**–**n** (0.1 mmol), nitromethane (1.2 mmol), **C1** (0.05 mmol), toluene (1 mL), room temperature. Yield after column chromatography. Enantiomeric ratio determined by chiral HPLC analysis. ^b^Reaction performed with 10 mol % **C1** and 18 equiv nitromethane. ^c^Reaction performed with 10 mol % **C1** in nitromethane.

Increasing the steric bulk of the C3 substituent slightly decreased the enantiomeric ratio but did not affect yields, except for bulky *tert*-butyl groups, which prevented the reaction. A phenyl group at C3 also reduced yield and enantioselectivity. Ketimines with *para*-substituted aryl groups at N1 showed similar reactivity and enantioselectivity, whereas *ortho*- and *meta*-substituents performed less efficiently. *N*-Alkyl-substituted ketimines reacted faster than *N*-aryl analogues but with lower enantiomeric ratios. Finally, using nitroethane instead of nitromethane gave good yields with moderate diastereoselectivity.

After exploring the scope and limitations of the aza-Henry reaction catalyzed by the homogeneous oxindole-containing thiourea organocatalyst, we proceeded to prepare heterogeneous analogues of the bifunctional thiourea **C1**, immobilized on the PIM copolymer framework. These analogues are designed for use in the same reaction under both batch and continuous flow conditions.

The target monomer **M1** was designed for the synthesis of a PIM copolymer via a condensation reaction between its two catechol groups and 2,3,5,6-tetrafluoroterephthalonitrile (TFTPN), using 5,5′,6,6′-tetrahydroxy-3,3,3′,3′-tetramethylspirobisindane (TTSBI) as a comonomer (Scheme 3). The synthesis of **M1** was accomplished in two steps: initially, isatin underwent an S_N_2 reaction with *N*-(2-bromoethyl)­phthalimide to afford an *N*-ethylphthalimido isatin intermediate. This protected isatin was then efficiently converted into the 3,3-diaryloxindole monomer **M1** via a condensation reaction with catechol, promoted by in situ-generated Lambert salt,^45^ yielding **M1**.

**Scheme 3:**
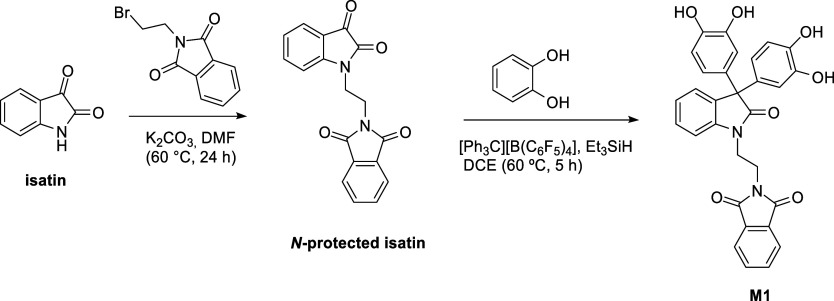
Synthesis of Monomer **M1**

The aim of synthesizing these PIM copolymers was to incorporate a reduced number of isatin groups into the polymer backbone, thereby decreasing the density of the catalytic sites. This strategy not only reduces material costs but also increases the spatial separation between the active centers. Consequently, the resulting heterogeneous catalyst is expected to exhibit enhanced long-term stability while preserving the intrinsic microporosity properties characteristic of PIMs.

Copolymers designated as **PIM-10** and **PIM-20** were synthesized via condensation of the tetrahydroxy monomer **M1** and 5,5′,6,6′-tetrahydroxy-3,3,3′,3′-tetramethylspirobisindane (TTSBI) with 2,3,5,6-tetrafluoroterephthalonitrile (TFTPN) in 1:9 (10%) and 1:4 (20%) molar ratios, respectively. The reaction was catalyzed by K_2_CO_3_, following the well-established polymerization protocol for PIMs,^5^ based on the formation of benzodioxin linkages.

Subsequently, the phthalimide protecting groups were removed by treatment with hydrazine hydrate in methanol at 40 °C,^13^ affording **Amine-PIM-10** and **Amine-PIM-20**, both functionalized with ethylene-linked amino groups. Due to the moderate solubility of these polymers in chloroform, quinine-derived thioureas **PIM-10-TU** and **PIM-20-TU** were synthesized by reacting **Amine-PIM-10** and **Amine-PIM-20** with the isothiocyanate QN-NCS in chloroform at 50 °C (Scheme 4).

**Scheme 4:**
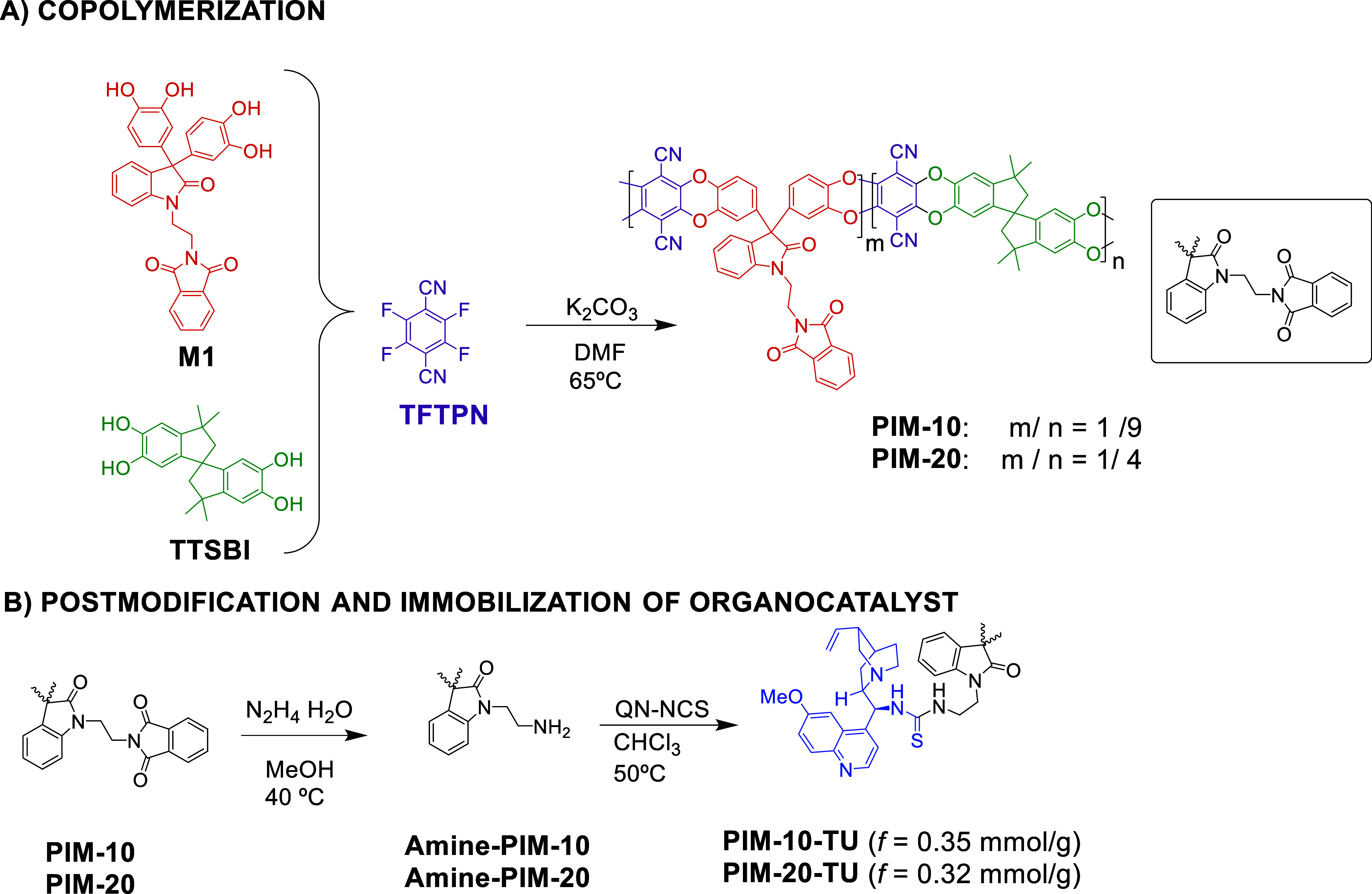
Synthesis of Copolymers **PIM-10** and **PIM-20** (A), Followed by Post-Functionalization and Immobilization of the Chiral Catalyst Onto the PIM Copolymers (B)

The effective functionalization (*f*) of heterogeneous catalysts **PIM-10-TU** and **PIM-20-TU** was determined to be 0.35 and 0.32 mmol g^–1^, respectively, based on sulfur elemental analysis. These results indicate that increasing the number of amino groups did not correspond to a proportional increase in catalytic site density.

### 3.2 Characterization of Polymer Precursors and Heterogeneous Catalysts

The precursor polymers **PIM-10** and **PIM-20** were obtained in nearly quantitative yields. Complete deprotection of the phthalimide groups afforded **Amine-PIM-10** and **Amine-PIM-20**. The composition of the copolymers and successful amine deprotection were confirmed by ^1^H NMR analysis, as evidenced by the disappearance of characteristic phthalimide signals in the spectra of the amine-functionalized polymers. Representative spectra for **PIM-10** and its derivatives are shown in Figure 1; data for **PIM-20** and related polymers are provided in Section S4 of the Supporting Information.

**Figure 1:**
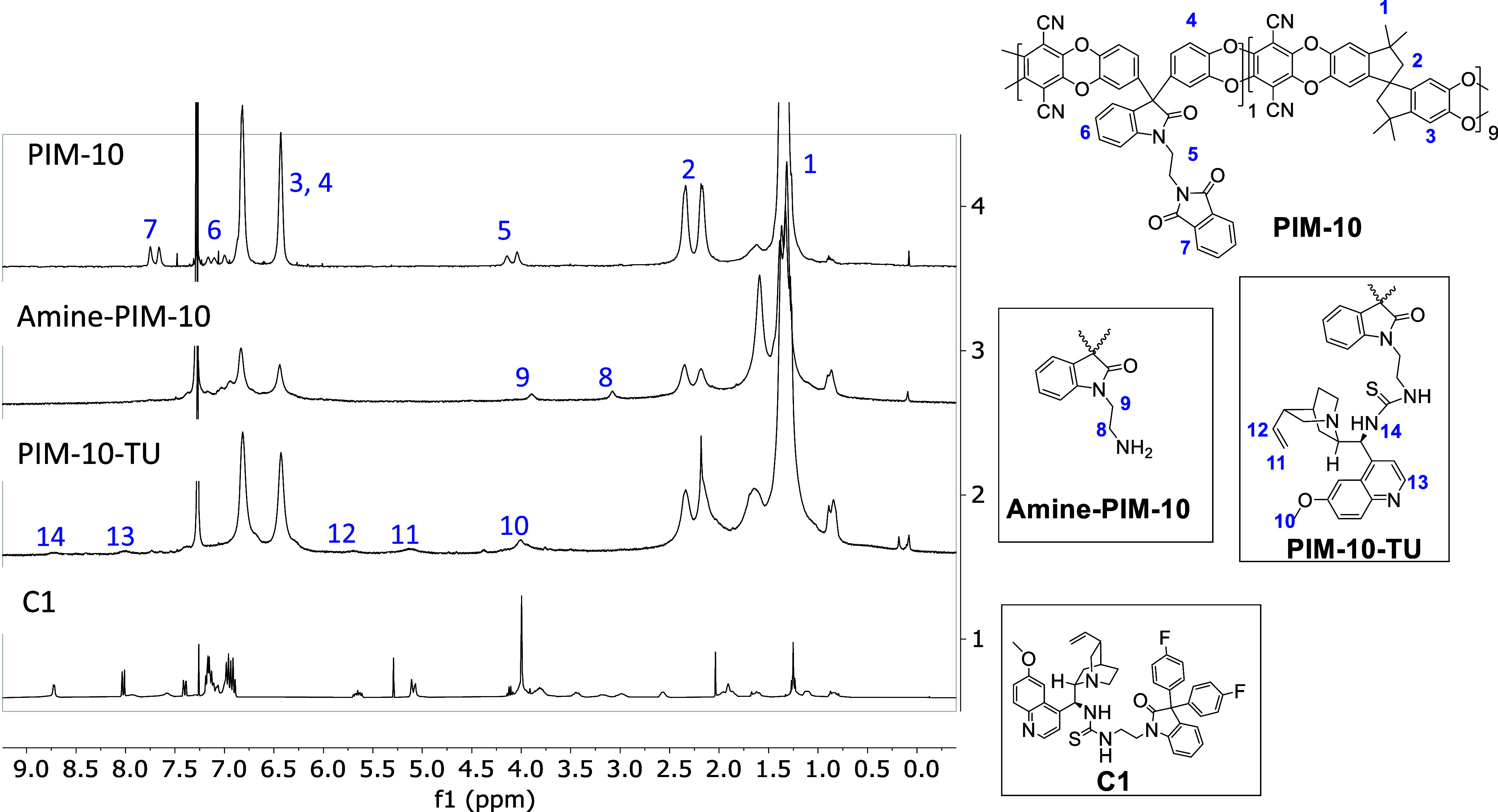
^1^H NMR spectra in CDCl_3_ of precursor **PIM-10**, **amine-PIM-10**, catalyst **PIM-10-TU**, and **C1**.

The ^1^H NMR spectra of the series **PIM-10** clearly display characteristic signals of the PIM-1 backbone structure,^46^ corresponding to the central segments of the spiro monomer residues observed in all spectra: methyl protons (1), methylene protons (2), and aromatic protons (3). Additionally, signals attributed to the ethylenic spacer (5), as well as aromatic protons from catechol (4), isatin (6), and the phthalimide group (7), are also present. In the ^1^H NMR spectrum of **Amine-PIM-10**, the disappearance of signals associated with the phthalimide moiety, along with changes in the ethylenic spacer region (8, 9), attributed to the presence of free amine groups, confirms complete deprotection. The incorporation of quinine-derived thiourea into **PIM-10-TU** results in reduced solubility in CDCl_3_. For comparison, the spectrum of catalyst **C1** is also shown in Figure 1. Signals labeled 10–14, corresponding to the methoxy group (MeO−), the double bond, and the pyridine moiety, are clearly observable and confirm the successful incorporation of the quinine unit into the polymeric catalyst. Additionally, the ^13^C NMR CPMAS spectra of the heterogeneous catalysts are provided in the Supporting Information (Figure S26). Signals in the aliphatic region (70–25 ppm) are attributed to the indane ring and the bicyclic quinine moiety. The CN resonance appears at approximately δ = 111 ppm, while signals in the δ = 160–120 ppm range correspond to aromatic phenyl carbons. The C=S and C=O groups are tentatively assigned around 185 and 160 ppm, respectively.

All copolymers were found to be soluble in chloroform and THF but insoluble in other common organic solvents such as dichloromethane, DMSO, DMF, ethyl acetate, acetone, toluene, 1,4-dioxane, and methanol.

According to the IUPAC classification of N_2_ adsorption isotherms, precursor **PIM-10** exhibits a microporous profile (Figure S30, Supporting Information). The Brunauer–Emmett–Teller-specific surface area was 644 m^2^ g^–1^, slightly lower than that reported for **PIM-1** powder (864 m^2^ g^–1^).^46^

Figure 2 shows that both precursor copolymers, **PIM-10** and **PIM-20**, exhibit high thermal stability with degradation onset temperatures above 350 °C under a nitrogen atmosphere. The thermal degradation profiles show minimal variation, suggesting that the PIM-1 backbone remains largely intact.^47^ Notably, a carbonaceous residue of over 90% remains at approximately 500 °C, further confirming the thermal robustness of these materials. In contrast, the Amine-PIM derivatives and the quinine-derived catalysts display reduced thermal stability, with degradation initiating around 300 °C. This decrease is attributed to thermal decomposition of the amine functionalities and thiourea groups.

**Figure 2:**
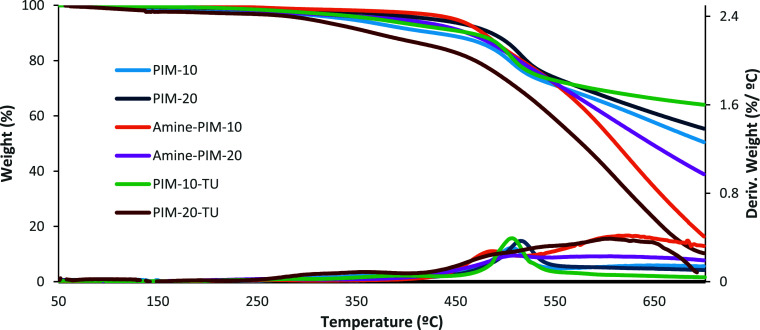
TGA curves recorded under a nitrogen atmosphere for the precursor polymers (**PIM-10** and **PIM-20**), their amine-functionalized derivatives (**Amine-PIM-10** and **Amine-PIM-20**), and the corresponding heterogeneous catalysts (**PIM-10-TU** and **PIM-20-TU**).

The FT-IR analyses were performed (Supporting Information, Section S5); however, no significant differences were observed between the copolymers and the characteristic spectrum of PIM-1.

The surface morphologies of the polymers (precursor and catalyst powders derived from PIM-10) were analyzed by SEM, as shown in Figure 3.

**Figure 3:**
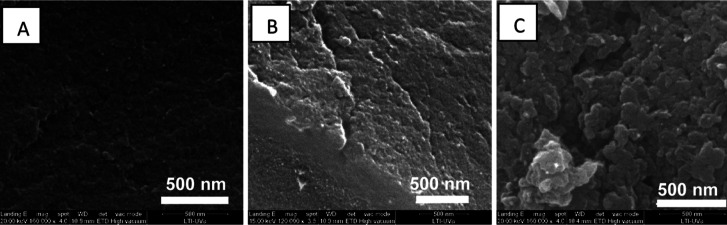
SEM images of the precursor and functionalized polymer samples: (A) **PIM-10**, (B) **Amine-PIM-10**, and (C) **PIM-10-TU**.

SEM provides valuable information about surface morphology, particle size, and material distribution at the micro- and nanoscale. Inspection of the micrographs reveals that the incorporation of quinine moieties increases the surface roughness, showing clusters of nanoaggregates distributed across the surface. In contrast, both precursors, PIM-10 and Amine-PIM-10, exhibit features characteristic of a homogeneous system, forming small concavities at the smoothed surface. SEM micrographs for PIM-20 have been included in the Supporting Information (Section S6). Additionally, AFM images and roughness data have been provided to complement the surface morphology analysis, which is also available in Section S6 of the Supporting Information.

Furthermore, based on the intrinsic microporosity indicated by the type of N_2_ isotherms, we concluded that PIM-10 belongs to the PIM family, which is characterized by a large number of interconnected pores with diameters smaller than 2 nm and a high surface area (e.g., 500–900 m^2^/g). However, this pore size for dry samples may not fully explain their porous behavior when they are swollen in the solvent used for asymmetric reactions.

### 3.3 Catalytic Performance of the Heterogeneous Catalyst in Batch and Flow Systems

The catalytic activity and recyclability of thiourea-functionalized polymers **PIM-10-TU** and **PIM-20-TU** were evaluated in the asymmetric aza-Henry reaction of pyrazolinone ketimine **1a** with nitromethane in toluene at room temperature (Table 1).

**Table 1:**
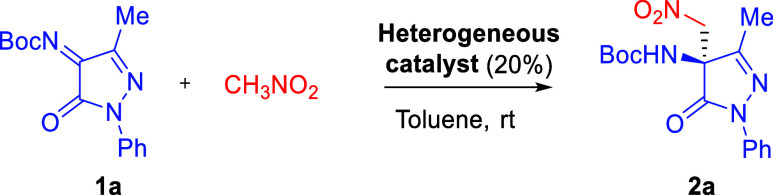
Asymmetric Aza-Henry Reaction of Pyrazolinone Ketimine **1a** Catalyzed by Immobilized Catalysts and Recycling Studies

entry^a^	catalyst	MeNO_2_ (n° equiv)	*t* (h)	conversion (%)^b^	**2a** (yield)^c^	er^d^
1	**PIM-10-TU**	12	8	100	85	84:16
2	**PIM-10-TU**	24	4	100	85	**85:15**
3	**PIM-10-TU**	48	2	100	87	83:17
4	**PIM-10-TU**	neat	4	100	88	72:28
5	**PIM-20-TU**	12	24	100	80	79:21
6	**PIM-20-TU**	24	8	100	83	80:20
7^e^	**PIM-10-TU**	24	4	95	86	85:15
8^e^	**PIM-10-TU**	24	4	78	79	84:16
9^e^	**PIM-10-TU**	24	4	50		-
			24	100	82	84:16
10^e^	**PIM-10-TU**	24	4	35	-	-
			24	100	83	84:16

a Reactions performed with ketimine **1a** (0.1 mmol), nitromethane (12–48 equiv), and the catalyst (20 mol %) in 1 mL of toluene at rt.

b Determined by ^1^H NMR.

c Isolated yield.

d Determined by chiral HPLC.

e Entries 7–10 correspond to recycling experiments (2–5) for entry 2.

Tests carried out with the polymeric thiourea **PIM-10-TU** in toluene, using different proportions of nitromethane, show that increasing the number of nitromethane equivalents significantly reduces the reaction time from 8 to 2 h, albeit with a slight decrease in enantiomeric ratio (from 85:15 to 83:17) (entries 1–3). Notably, performing the reaction in pure nitromethane leads to a further decline in enantioselectivity (er 72:28, entry 4), consistent with observations for the model thiourea catalyst **C1** (Table S1, entry 11, Supporting Information). Furthermore, a higher nitromethane content was found to promote polymer gelation, which hampers its use in flow chemistry due to column clogging.

A similar reduction in reaction time with increasing nitromethane equivalents was observed for the polymeric thiourea **PIM-20-TU** (entries 5–6). However, the enantiomeric ratio obtained with this catalyst was lower than that with **PIM-10-TU**, thereby leading to the selection of the latter for further investigations.

The recyclability of **PIM-10-TU**, owing to its heterogeneous nature, was also evaluated. The immobilized thiourea was recovered by centrifugation, washed with toluene, and reused over five consecutive reaction cycles (entries 2 and 7–10). A decline in catalytic activity was observed after the third cycle; however, the enantioselectivity of the product remained largely unaffected.

The efficiency of the immobilized thiourea catalyst **PIM-10-TU** was subsequently evaluated in a continuous-flow process (Scheme 5). The setup consisted of an Omnifit column (6.6 mm ID.) packed with 1.30 g of supported catalyst (*f* = 0.35 mmol g^–1^), connected to a THALESNano micro HPLC pump. Initially, toluene and a toluene/nitromethane (8:1) mixture were flushed through the system at a flow rate of 0.1 mL min^–1^ for 30 min to swell the catalyst. Subsequently, a solution of ketimine **1a** (518 mg, 1.8 mmol) in 18 mL of toluene/nitromethane (8:1, 0.1 M) was pumped through the column for 3 h at the same flow rate (see the continuous-flow device in the Supporting Information).

**Scheme 5:**
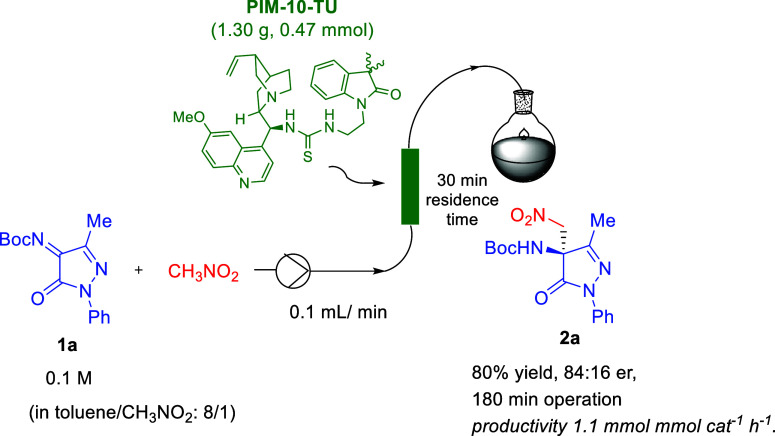
Continuous-Flow Aza-Henry Reaction of Pyrazolinone Ketimine **1a** Catalyzed by Immobilized **PIM-10-TU**

Higher nitromethane proportions induced polymer gelation, resulting in column clogging. The process was monitored by ^1^H NMR, confirming the complete conversion after 3 h. The reaction mixture was concentrated and purified by flash chromatography, affording product **2a** in an 80% isolated yield (502 mg, 1.44 mmol) with a good enantiomeric ratio (84:16 er). These results correspond to an effective catalyst loading of 25 mol %, an accumulated TON of 3.3, and a productivity of 1.1 mmol cat^–1^ h^–1^ for the synthesis of **2a**.

The residence time under flow conditions was 30 min, markedly shorter than the 4 h reaction time required to achieve full conversion under batch conditions (entry 2, Table 1). Additionally, the enantiomeric ratio obtained under flow conditions was only slightly lower than that observed in the batch reaction under similar conditions (85:15 er). Notably, recrystallization of adduct **2a** from hexane-ethyl acetate afforded nearly enantiopure material (98:2 er) in 75% yield from the mother liquor.

### 3.4 Synthetic Utility of Practically Enantiopure 4-Aminopyrazolone Derivatives

The synthetic relevance of the aza-Henry reaction products was exemplified by the transformation of enantioenriched adduct **2a** into 4-aminopyrazolone derivatives **7a**–**10a**, compounds of potential pharmacological interest (Scheme 6).^48^

**Scheme 6:**
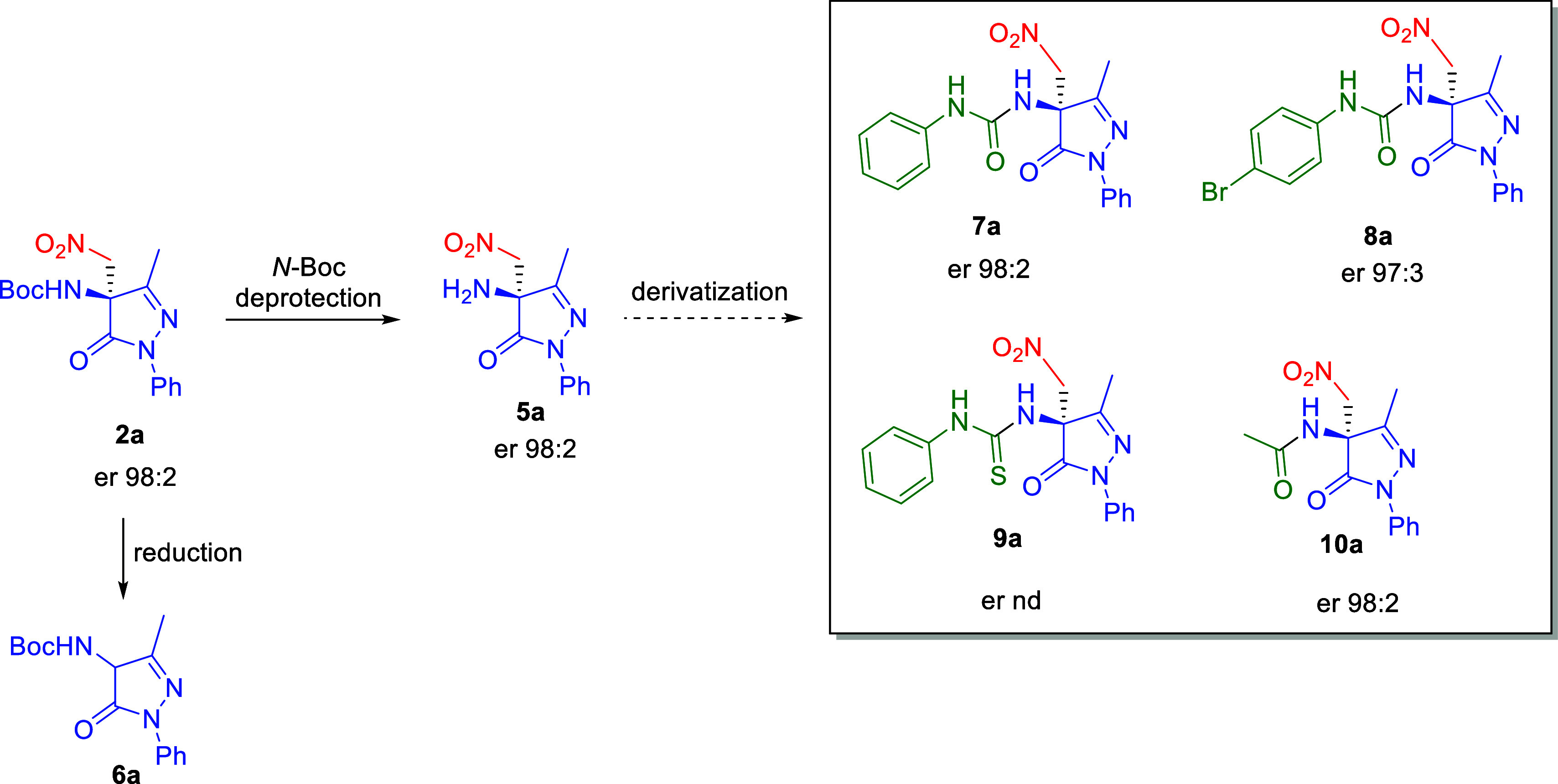
Derivatization of Compound **2a** to Enantioenriched 4-Aminopyrazolone Derivatives

Deprotection of the *N*-Boc group in compound **2a** using 4 M HCl in dioxane at room temperature afforded the free nitro amine **5a** in excellent yield. This intermediate was further derivatized into enantioenriched ureas **7a**–**8a**, thiourea **9a**, and acetamide **10a**. Importantly, chiral HPLC analysis of these compounds confirmed that no erosion of the enantiomeric purity occurred throughout the entire synthetic sequence. However, reduction of the nitro group in **2a** using NaBH_4_/NiCl_2_ ^19^ led to racemic *N*-Boc-protected amine **6a**. This outcome likely results from a methanol-promoted retro aza-Henry reaction,^49^ generating *N*-Boc ketimine **1a**, which is then reduced by NaBH_4_. Notably, this compound was also obtained in quantitative yield by the direct reduction of ketimine **1a** with NaBH_4_.

## 4 Conclusions

In summary, we have developed PIM-based heterogeneous organocatalysts incorporating quinine-derived thioureas for the enantioselective aza-Henry reaction of *N*-Boc pyrazolinone ketimines. The use of flexible isatin-based monomers enabled the synthesis of copolymers with enhanced swelling and porosity suitable for accommodating bulky substrates. Among the two materials prepared, PIM-10-TU exhibited a superior catalytic performance, achieving high yields and enantioselectivities under mild conditions in both batch and continuous-flow setups. Furthermore, the synthetic applicability of the resulting chiral β-nitroamines was demonstrated through derivatization into enantioenriched ureas and thioureas. These results showcase the potential of functionalized PIMs as versatile, efficient, and recyclable platforms for asymmetric synthesis in both academic and industrial contexts.

## Supplementary Material



## Notes

Model catalyst screening, derivatization of compound **2a**, experimental section (synthesis of monomer M1), and characterization of new compounds and polymers (PDF)
