# Supplementary material for: Tailored Catalysts Based on Polymers of Intrinsic Microporosity for Asymmetric Aza-Henry Reaction of Pyrazolinone Ketimines in Batch and Flow
Source: ACS Appl Polym Mater. 2025 Oct 6;7(20):13626–36. doi: 10.1021/acsapm.5c02439 (PMC12560148; doi:10.1021/acsapm.5c02439)
Supplement: Supplementary file 1 [file ap5c02439_si_001.pdf]

SUPPORTING INFORMATION

Tailored Catalysts based on Polymers of Intrinsic Microporosity for Asymmetric Aza-Henry Reaction of Pyrazolinone Ketimines in Batch and Flow

Rodrigo Sánchez-Molpeceres,[a] Paolo Zhan,[a] Laura Martín, *[a], Alicia Maestro,[a] Jesús A. Miguel, [b] Bibiana Comesaña-Gándara, [b] and José M. Andrés[a]*

[a]GIR SintACat, IU CINQUIMA/ Química Orgánica, Facultad de Ciencias, Universidad de Valladolid, Paseo Belén 7, 47011-Valladolid, Spain

E-mail: jmandres@uva.es, laura.martinm@uva.es

[b]IU CINQUIMA/ Química Inorgánica, Facultad de Ciencias, Universidad de Valladolid, Paseo Belén 7, 47011-Valladolid, Spain

Table of Contents

1. Optimization and Screening of Reaction Conditions for Model Catalysts (**C1**–**C9**) S2
2. Transformations of Adduct **2a** into Enantioenriched 4-Aminopyrazolone Derivatives **7a-9a** S5
3. Synthesis Procedures and Characterization of New Compounds and Polymers. Continuous-flow device. S6
4. NMR Spectra of New Compounds. S18
5. FTIR Spectra of Polymers. S41
6. SEM Micrographs of PIM-20 Family. AFM images. S42
7. N_2 Sorption Isotherm of the PIM-10 Powder at 77K. S43
8. HPLC profiles. S44

1. Optimization and Screening of Reaction Conditions for Model Catalysts (C1–C9).

Table S1. Catalyst screening and optimization of reaction conditions.[a]

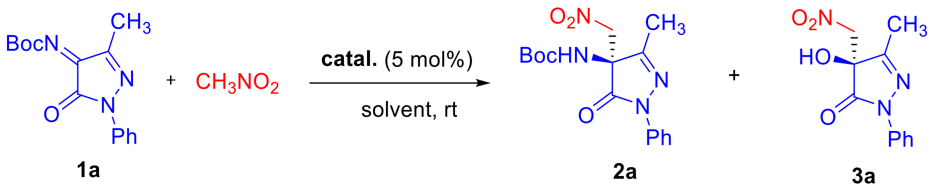

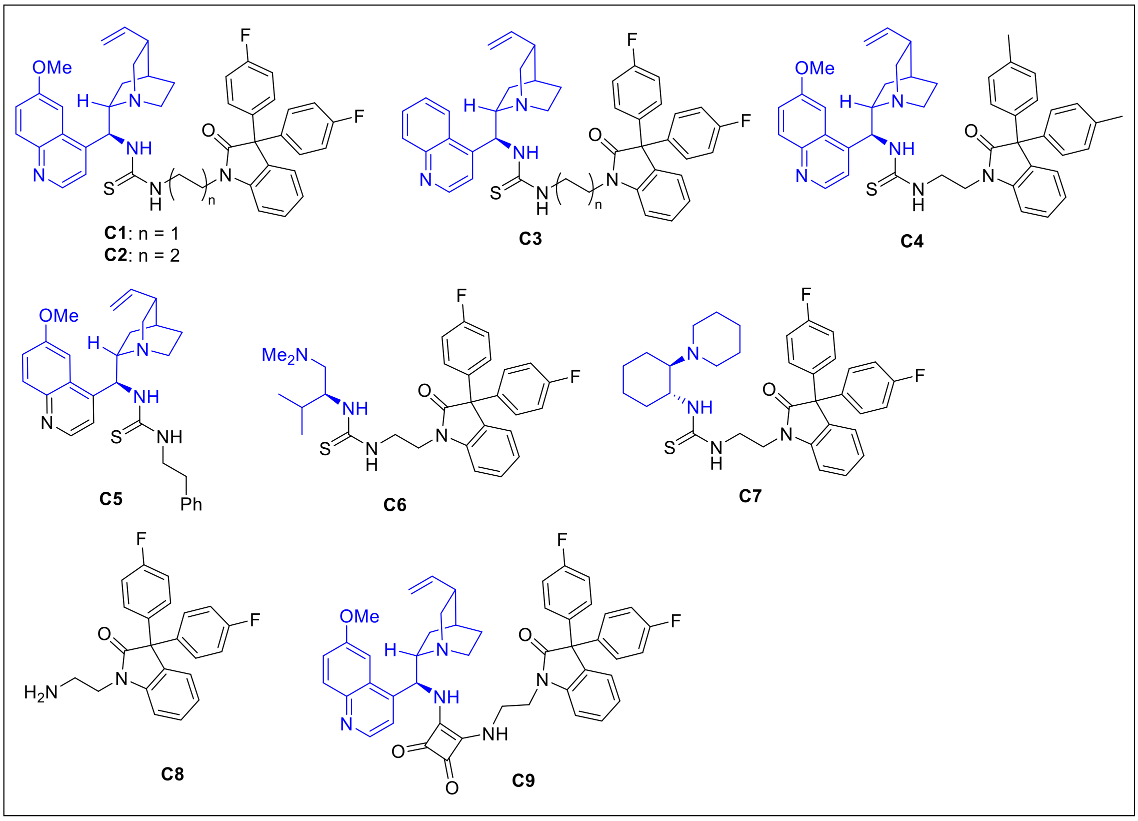

Entry	Catalyst	Solvent	t (h)	1a (%)[b]	2a (%)[b]	3a (%)[b]	2a (Yield)[c]	Er[d]
1	**C1**	THF	6	0	100	0	75	82:18
2	**C2**	THF	5	26	74	0	61	79:21
3	**C3**	THF	6	0	100	0	88	80:20
4	**C4**	THF	6	0	100	0	68	78:22
5	**C5**	THF	7	6	94	0	66	74:26

6	**C6**	THF	2	0	100	0	63	81:19
7	**C7**	THF	8	0	100	0	60	34:66
8	**C8**	THF	24	100	0	0	-	-
9[e]	**C1**	THF	4	0	100	0	85	82:18
10[e]	**C1**	2-MeTHF	6	0	100	0	86	83:17
11[e]	**C1**	MTBE	7	0	100	0	72	86:14
12[e]	**C1**	DCE	8	0	100	0	72	82:18
13[e]	**C1**	$CHCl_3$	8	0	100	0	83	86:14
14[e]	**C1**	MeCN	7	25	75	0	36	83:17
15[e]	**C1**	EtOAc	6	0	100	0	83	85:15
16[e]	**C1**	Acetone	12	0	100	0	88	85:15
17[e]	**C1**	IPA	12	0	100	0	86	83:17
18[e]	**C1**	Toluene	4	4	96	0	75	**89:11**
19	**C1**	$MeNO_2$	1	3	93	4	90	85:15
20[e,f]	**C1**	Toluene	6	0	100	0	89	88:12
21[e,g]	**C1**	Toluene	3	0	100	0	91	88:12
22[e]	**C9**	Toluene	2	0	100	0	77	86:14

[a]Reactions performed with ketimine **1a** (0.1 mmol), nitromethane (0.6 mmol, 6 equiv.), and the catalyst (0.05 mmol, 0.05 equiv.) in 1 mL of solvent at rt.[b] Determined by ^1H NMR. [c]Isolated yield. [d]Determined by chiral HPLC. [e]Reaction performed with 12 equiv. nitromethane. [f]Reaction performed with 2 mol% of catalyst **C1**. [g]Reaction performed with 10 mol % of catalyst **C1**.

First, we investigated the reaction of ketimine **1a** with nitrometane (6 equiv.) in the presence of 5 mol% of quinine-derived bifunctional thiourea **C1** in THF at room temperature. The reaction was completed within 6 h, providing adduct **2a** in 63% yield and an 82:18 enantiomeric ratio (er) (entry 1).

Quinine-derived thiourea **C2**, which contains a four-methylene spacer, delivered the desired product **2a** in good yield but with lower enantioselectivity (79:21 er, entry 2). When the reaction was carried out using thiourea **C3**, derived from cinchonidine, the absence of the methoxy group on the quinoline ring led to a slightly reduced enantiomeric ratio (80:20 er, entry 3). Similarly, quinine-derived thiourea **C4**, bearing a 3,3-di-*p*-tolyloxindole scaffold, did not enhance enantioselectivity, highlighting the beneficial effect of fluorine atoms on the estereoselectivity (78:22 er, entry 4).

The importance of the 3,3-diaryloxindole motif was further illustrated by thiourea **C5**, wich features a phenethyl substituent and showed significantly poorer performance compared to **C1** (compare entries 1 and 5). A slight decrease in enantioselectivity was also observed when employing L-valine-derived thiourea **C6** (81:19 er, entry 6). Interestingly, (1*R*,2*R*)-cyclohexanediamine-derived thiourea **C7** catalyzed the reaction efficiently but provided the opposite enantiomer of **2a** with similar yield and lower selectivity (34:66 er, entry 7). The achiral ethylamino derivative **C8** failed to promote the transformation, and the starting material was fully recovered after 24 h (entry 8). Finally, increasing the amount of nitromethane to 12 equivalents significantly shortened the reaction time, improved the chemical yield, and had no detrimental effect on enantioselectivity (entry 9).

With **C1** identified as the most effective catalyst among those tested, we proceeded to investigate the influence of different solvents on the reaction outcome. A range of solvents was screened, including 2-MeTHF, MTBE, DCE, $CHCl_3$, MeCN, EtOAc, acetone, isopropanol (IPA), and toluene. Notably, the enantiomeric ratio was improved when the reaction was carried out in toluene (compare entry 18 with entries 9–17). In contrast, conducting the reaction in nitromethane resulted in a substantial drop in enantioselectivity, despite affording the highest chemical yield (90%) within just 1 hour (entry 18). We further examined the effect of catalyst loading and found that both increasing and decreasing the amount of catalyst led to slightly diminished results in terms of selectivity (entries 20–21). Lastly, the use of quinine-derived squaramide **C9** in toluene proved less effective than the corresponding thiourea **C1**, both in yield and enantioselectivity (see entries 18 and 22).

On the basis of our results and those previously reported with quinine-derived organocatalysts,[1] we proposed the formation of the ternary complex depicted in Figure S1 to rationalize the (*S*)-configuration of the products **2a-n**. The H-bonding activation of N-Boc ketamine **1** by the thiourea moiety of catalyst **C1** facilitates the nucleophilic attack of the nitronate anion from the *re*-face of the imine group, leading to the formation of adduct **2** with the (*S*) configuration.

[1] (a) P. Chauhan, S. Mahajan, U. Kaya, A. Peuronen, K. Rissanen, D. Enders *J. Org. Chem.* **2017**, *82*, 70508. (b) Y. Zhou, Y. You, Z. -H. Wang, X. -M. Zhang, X. -Y. Xu, W. -C. Yuan *Eur. J. Org. Chem.* **2019**, *2019*, 3112. (c) M. Gil-Ordóñez, C. Aubry, C. Niño, A. Maestro, J. M. Andrés. *Molecules* **2022**, *27*, 6983.

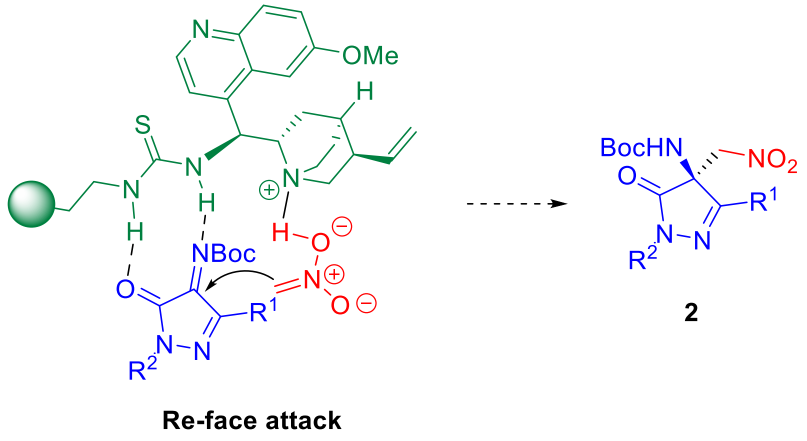

Figure S1.

2. Transformations of adduct 2a into enantioenriched 4-aminopyrazolone derivatives 7a-9a

Scheme S1. Derivatization of compound **2a** into enantioenriched 4-aminopyrazolone derivatives.

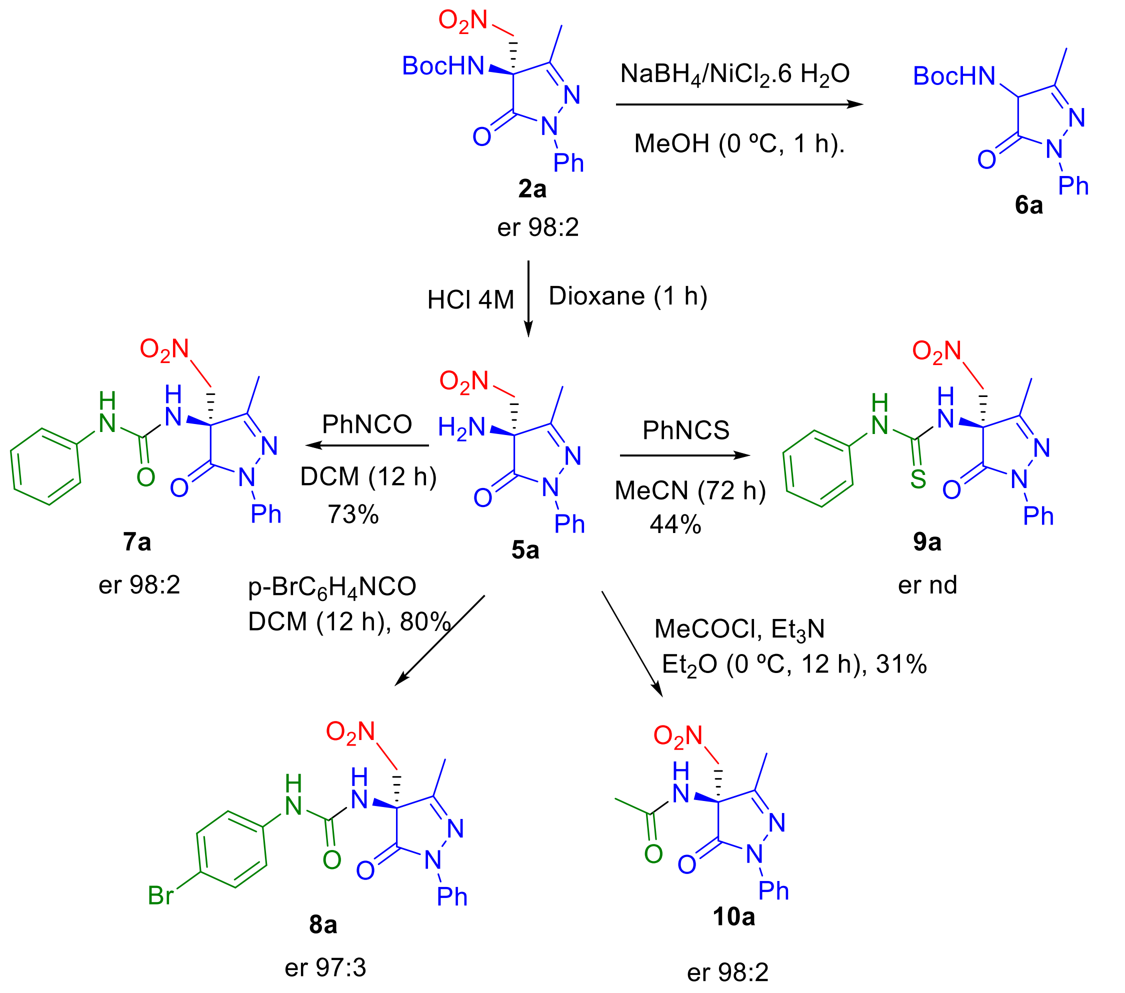

3. Synthesis procedures and characterization of new compounds and polymers

tert-Butyl (S)-(3-methyl-4-(nitromethyl)-5-oxo-1-phenyl-4,5-dihydro-1H-pyrazol-4-yl) carbamate (2a).

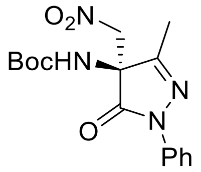

Product **2a** was obtained according to general procedure using 28.7 mg of N-Boc ketimine **1a** (1 mmol). Chromatography on a silica gel using Hexane/EtOAc = 4:1 as an eluent afforded compound **2a** as a colorless solid (21.5 mg, 0.075 mmol, 75% yield). **[α]$_D$** = -27.6 (c = 0.34, $CHCl_3$) for er 89:11. **^{1}H NMR** (500 MHz, $CDCl_3$): δ 7.86 (d, *J* = 8.8 Hz, 2H), 7.41 (t, *J* = 8.1 Hz, 2H), 7.22 (t, *J* = 7.4 Hz, 1H), 6.10 (br, 1H), 4.69 (d, *J* = 12.3 Hz, 1H), 4.60 (d, *J* = 12.3 Hz, 1H), 2.12 (s, 3H), 1.38 (s, 9H) ppm. **^{13}C NMR** (126 MHz, $CDCl_3$): δ 168.7, 157.2, 153.4, 137.5, 129.0 (2C), 125.8, 119.0 (2C), 82.7, 75.9, 64.0, 28.0 (3C), 13.7 ppm. **HPLC:** Chiralpak AD-H column, Hexane/iPrOH 85:15, 1 mL/min, λ = 254 nm, major enantiomer (S) t_r = 8.379 min, minor enantiomer (R) t_r =23.107 min (er **88:12**). **HRMS** (ESI–TOF) m/z: $[M+H]^+$ Calcd. for $C_{16}H_{20}N_4NaO_5$ 371.1334; Found 371.1326.

tert-Butyl (S)-(3-ethyl-4-(nitromethyl)-5-oxo-1-phenyl-4,5-dihydro-1H-pyrazol-4-yl) carbamate (2b)

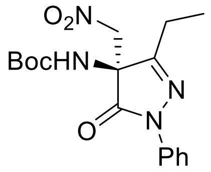

Product **2b** was obtained according to general procedure. Chromatography on a silica gel using Hexane/AcOEt = 4:1 as an eluent afforded compound **2b** as a colorless solid (29 mg, 0.080 mmol, 80% yield). [α]$_D$ = -22.41 (c = 0.58, $CHCl_3$) for er 88:12. **^{1}H NMR** (500 MHz, $CDCl_3$): δ 7.89 (d, J = 7.8 Hz, 2H), 7.41 (t, J = 7.9 Hz, 2H), 7.21 (t, J = 7.8 Hz, 1H), 6.12 (br, 1H), 4.68 (d, J = 12.4 Hz, 1H), 4.62 (d, J = 12.3 Hz, 1H), 2.40 (m, 2H), 1.38 (s, 9H), 1.31 (t, J = 7.3 Hz, 3H) ppm. **^{13}C NMR** (126 MHz, $CDCl_3$): δ 168.5, 160.8, 154.6, 137.6, 129.0 (2C), 125.7, 119.1, 82.5, 76.1, 64.0, 28.2 (3C), 21.4, 9.4 ppm. **HPLC:** Chiralpak AD-H column, Hexane/iPrOH 90:10, 1 mL/min, λ = 254 nm, major enantiomer (S) t_r = 7.950 min, minor enantiomer (R) t_r = 20.792 min. (er: **88:11**). **HRMS** (ESI–TOF) m/z: $[M+H]^+$ Calcd. for $C_{17}H_{22}N_4O_5$ 385.1482; Found 384.1480.

tert-Butyl (S)-(3-isopropyl-4-(nitromethyl)-5-oxo-1-phenyl-4,5-dihydro-1H-pyrazol-4-yl) carbamate (2c).

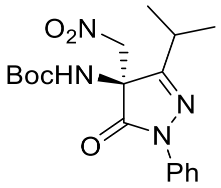

Product **2c** was obtained according to modified general procedure using catalyst **C1** (6 mg, 0.001 mmol, 0.10 equiv) and 18 equiv. of nitromethane. Chromatography on a silica gel using Hexane/AcOEt = 4:1 as an eluent afforded compound **2c** as a colorless solid (35 mg, 0.093 mmol, 93% yield). $[\alpha]_D$ = -19.85 (c = 0.66, $CHCl_3$) for er 85:15. ^{1}**H NMR** (500 MHz, $CDCl_3$): δ 7.90 (d, *J* = 8.0 Hz, 2H), 7.40 (t, *J* = 8.0 Hz, 2H), 7.20 (t, *J* = 7.4 Hz, 1H), 6.19 (br, 1H), 4.66 (d, *J* = 12.4 Hz, 1H), 4.64 (d, *J* = 12.3 Hz, 1H), 2.64 (m, 1H), 1.37 (s, 9H), 1.31(d, *J* = 6.8 Hz, 6H) ppm. ^{13}**C NMR** (126 MHz, $CDCl_3$): δ 168.3, 163.5, 153.2, 137.7, 128.9 (2C), 125.6, 119.0 (2C), 82.1, 76.2, 60.4, 28.5, 28.0 (3C), 21.5, 20.4 ppm. **HPLC:** Chiralpak AD-H column, Hexane/iPrOH 85:15, 1 mL/min, λ = 254 nm, major enantiomer (S) t_r = 7.158 min, minor enantiomer (R) t_r =16.883 min. (er: **85:15**). **HRMS** (ESI–TOF) m/z: $[M+H]^+$ Calcd. for $C_{18}H_{24}N_4O_5$ 399.1639; Found 399.1644.

tert-Butyl (S)-(3-cyclopropyl-4-(nitromethyl)-5-oxo-1-phenyl-4,5-dihydro-1H-pyrazol-4-yl) carbamate (2d).

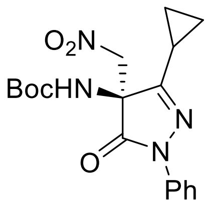

Product **2d** was obtained according to general procedure. Chromatography on a silica gel using Hexane/EtOAc = 4:1 as an eluent afforded compound **2d** as a colorless solid (26 mg, 0.070 mmol, 70% yield). $[\alpha]_D$ = -29.7 (c = 0.8, $CHCl_3$) for er 88:12.^{1}**H NMR** (500 MHz, $CDCl_3$): δ 7.87 (d, *J* = 7.7 Hz, 2H), 7.40 (t, *J* = 7.9 Hz, 2H), 7.21 (m, 1H), 6.22 (br, 1H), 4.73 (d, *J* = 12.5 Hz, 1H), 4.63 (d, *J* = 12.5 Hz, 1H), 1.54 (tt, *J* = 8.1, 4.9 Hz, 1H), 1.39 (s, 9H), 1.15 (m, 2H), 1.01 (m, 2H) ppm. ^{13}**C NMR** (101 MHz, $CDCl_3$): δ 168.2, 161.7, 153.1, 137.7, 128.9 (2C), 125.5, 118.8 (2C), 82.0, 76.2, 64.2, 28.0 (3C), 8.98, 8.28 ppm. **HPLC:** Chiralpak AD-H column, Hexane/iPrOH 85:15, 1 mL/min, λ = 254 nm, major enantiomer (S) t_r = 8.598 min, minor enantiomer (R) t_r =19.288 min. (er **88:12**). **HRMS** (ESI–TOF) m/z: $[M+H]^+$ Calcd. for $C_{18}H_{23}N_4O_5$ 375.1663; Found 375.1666.

tert-Butyl (S)-(4-(nitromethyl)-5-oxo-1,3-diphenyl-4,5-dihydro-1H-pyrazol-4-yl) carbamate (2f).

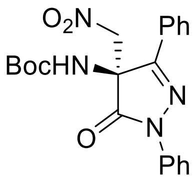

Product **2e** was obtained according to general procedure using catalyst **C1** (6 mg, 0.001 mmol, 0.10 equiv). Chromatography on a silica gel using Hexane/AcOEt = 4:1 as an eluent afforded compound **2e** as a colorless solid (20 mg, 0.054 mmol, 54% yield). **^{1}H NMR** (500 MHz, $CDCl_3$): δ 7.98 (dd, J = 16.2, 7.2 Hz, 5H), 7.54 – 7.42 (m, 6H), 7.27 (d, J = 7.4 Hz, 2H), 6.55 (br, 1H), 4.82 (d, J = 12.2 Hz, 1H), 4.65 (d, J = 12.2 Hz, 1H), 1.46 – 1.30 (s, 9H) ppm. **^{13}C NMR** (126 MHz, $CDCl_3$): δ 168.7, 155.0, 153.3, 137.6, 131.3, 129.2, 129.1 (2C), 129.0 (2C), 126.1 (2C), 126.0 , 119.4 (2C), 76.6, 63.4, 27.9 (3C) ppm. **HPLC:** Chiralpak AD-H column, Hexane/iPrOH 85:15, 1 mL/min, λ = 254 nm, major enantiomer (S) t_r = 15.617 min, minor enantiomer (R) t_r =57.167 min. (er: **73:27**). **HRMS** (ESI–TOF) m/z: $[M+H]^+$ Calcd. for $C_{21}H_{22}N_4NaO_5$ 433.1482; Found 433.1495.

tert-Butyl (S)-(3-methyl-4-(nitromethyl)-5-oxo-1-(p-tolyl)-4,5-dihydro-1H-pyrazol-4-yl) carbamate (2g).

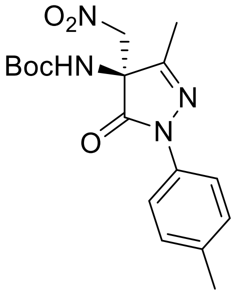

Product **2g** was obtained according to general procedure. Chromatography on a silica gel using Hexane/AcOEt = 4:1 as an eluent afforded compound **2g** as a colorless solid (33 mg, 0.086 mmol, 86% yield). $[\alpha]_D$ = -13.75 (c = 0.64, $CHCl_3$) for er 89:11. **^{1}H NMR** (400 MHz, $CDCl_3$): δ 7.72 (d, J = 8.6 Hz, 2H), 7.20 (t, J = 8.1 Hz, 2H), 6.09 (br, 1H), 4.68 (d, J = 12.2 Hz, 1H), 4.59 (d, J = 12.2 Hz, 1H), 2.35 (s, 3H), 1.37 (s, 9H) ppm. **^{13}C NMR** (101 MHz, $CDCl_3$): δ 168.1, 156.9, 135.5, 135.0, 129.5 (2C), 119.0 (2C), 76.0, 63.8, 28.0 (3C), 20.98, 13.8 ppm. **HPLC:** Chiralpak AD-H column, Hexane/iPrOH 85:15, 1 mL/min, λ = 254 nm, major enantiomer (S) t_r = 11.233 min, minor enantiomer (R) t_r =25.700 min. (er: **89:11**). **HRMS** (ESI–TOF) m/z: $[M+H]^+$ Calcd. for $C_{17}H_{22}N_4O_5$ 385.1482; Found 385.1482.

tert-Butyl (S)-(1-(4-bromophenyl)-3-methyl-4-(nitromethyl)-5-oxo-4,5-dihydro-1H-pyrazol-4-yl) carbamate (2h).

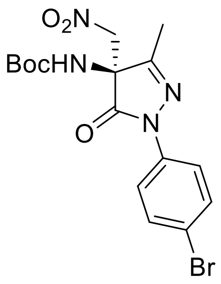

Product **2h** was obtained according to general procedure. Chromatography on a silica gel using Hexane/EtOAc = 4:1 as an eluent afforded compound **2h** as a colorless solid (30 mg, 0.071 mmol, 71% yield). **[α]D** = -18.6 (c = 0.6, $CHCl_3$) for er 88:12. **^{1}H NMR** (500 MHz, $CDCl_3$): δ 7.82 (m, 2H), 7.54 (d, 2H), 6.07 (br, 1H), 4.69 (d, *J* = 12.3 Hz, 1H), 4.62 (d, *J* = 12.3 Hz, 1H), 2.14 (s, 3H), 1.40 (s, 9H) ppm. **^{13}C NMR** (126 MHz, $CDCl_3$): δ 168.3, 157.3, 153.3, 136.6, 132.0 (2C), 120.3 (2C), 118.7, 82.7, 76.0, 28.0 (3C), 13.8 ppm. **HPLC:** Chiralpak AD-H column, Hexane/iPrOH 85:15, 1 mL/min, λ = 254 nm, major enantiomer (S) t_r = 11.357 min, minor enantiomer (R) t_r =15.176 min. (er **88:12**). **HRMS** (ESI–TOF) m/z: $[M+Na]^+$ Calcd. for $C_{16}H_{19}BrN_4NaO_5$ 449.0431; Found 449.0435.

tert-Butyl (S)-(1-(4-chlorophenyl)-3-methyl-4-(nitromethyl)-5-oxo-4,5-dihydro-1H-pyrazol-4-yl) carbamate (2i).

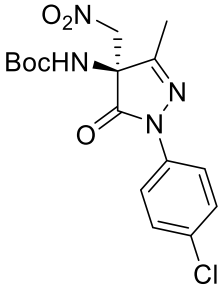

Product **2i** was obtained according to general procedure. Chromatography on a silica gel using Hexane/EtOAc = 4:1 as an eluent afforded compound **2i** as a colorless solid (33 mg, 0.086 mmol, 86% yield). **[α]D** = -13.8 (c = 0.64, $CHCl_3$) for er 87:13. **^{1}H NMR** (400 MHz, $CDCl_3$): δ 7.85 (d, *J* = 9.0 Hz, 2H), 7.37 (t, *J* = 9.0 Hz, 2H), 6.06 (br, 1H), 4.68 (d, *J* = 12.3 Hz, 1H), 4.60 (d, *J* = 12.2 Hz, 1H), 2.12 (s, 3H), 1.38 (s, 9H) ppm. **^{13}C NMR** (126 MHz, $CDCl_3$): δ 168.3, 157.3 (C), 153.3, 136.1, 130.9, 129.0, 120.0 (2C), 82.7, 75.9, 63.8, 28.0 (3C), 13.8 ppm. **HPLC:** Chiralpak AD-H column, Hexane/iPrOH 85:15, 1 mL/min, λ = 254 nm, major enantiomer (S) t_r = 8.858 min, minor enantiomer (R) t_r =18.792 min. (er **87:13**). **HRMS** (ESI–TOF) m/z: $[M+H]^+$ Calcd. for $C_{16}H_{19}ClN_4O_5$ 405.0936; Found 405.0939.

tert-Butyl (S)-(1-(3-chlorophenyl)-3-methyl-4-(nitromethyl)-5-oxo-4,5-dihydro-1H-pyrazol-4-yl) carbamate (2j).

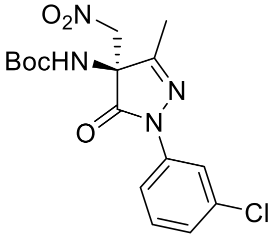

Product **2j** was obtained according to general procedure. Chromatography on a silica gel using Hexane/EtOAc = 6:1 to 4:1 as an eluent afforded compound **2j** as a colorless oil (27 mg, 0.07 mmol, 70% yield). **[α]D** = -22.0 (c = 0.64, $CHCl_3$) for er 83:17. **^{1}H NMR** (500 MHz, $CDCl_3$): δ 7.95 (s, 1H), 7.84 (m, 1H), 7.34 (t, *J* = 8.2 Hz, 1H), 7.20 (m, 1H), 6.09 (br, 1H), 4.69 (d, *J* = 12.3 Hz, 1H), 4.62 (d, *J* = 12.3 Hz, 1H), 2.14 (s, 3H), 1.39 (s, 9H) ppm. **^{13}C NMR** (126 MHz, $CDCl_3$): δ 168.3, 157.3, 138.5, 134.8, 130.0, 125.7, 118.8, 116.6, 75.9, 63.9, 28.0 (3C), 13.8 ppm. **HPLC:** Chiralpak AD-H column, Hexane/iPrOH 85:15, 1 mL/min, λ = 254 nm, major enantiomer (S) t_r = 6.56 min, minor enantiomer (R) t_r =15.033 min. (er **83:17**). **HRMS** (ESI–TOF) m/z: $[M+Na]^+$ Calcd. for $C_{16}H_{19}ClN_4NaO_5$ 405.0936; Found 405.0942.

tert-butyl (S)-(1-(2-chlorophenyl)-3-methyl-4-(nitromethyl)-5-oxo-4,5-dihydro-1H-pyrazol-4-yl) carbamate (2k).

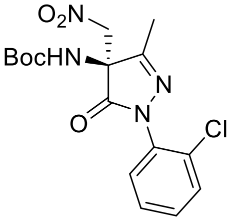

Product **2k** was obtained according to general procedure. Chromatography on a silica gel using Hexane/EtOAc = 6:1 to 4:1 as an eluent afforded compound **2k** as a colorless oil (29 mg, 0.076 mmol, 76% yield). **[α]D** = -48.3 (c = 0.46, $CHCl_3$) for er 78:22. **^{1}H NMR** (500 MHz, CDCl3): δ 7.52 (m, 2H), 7.36 (m, 2H), 6.09 (br, 1H), 4.75 (d, J = 12.4 Hz, 1H), 4.68 (d, J = 12.3 Hz, 1H), 2.10 (s, 3H), 1.46 (s, 9H) ppm. **^{13}C NMR** (126 MHz, $CDCl_3$): δ 169.2, 157.1, 153.5, 134.0, 131.6, 130.3, 130.2, 128.7, 127.7, 82.3, 75.9, 62.7, 28.1 (3C), 13.9 ppm. **HPLC:** Chiralpak AD-H column, Hexane/iPrOH 85:15, 1 mL/min, λ = 254 nm, major enantiomer (S) t_r = 7.121 min, minor enantiomer (R) t_r =11.265 min. (er **77:23**). **HRMS** (ESI–TOF) m/z: $[M+Na]^+$ Calcd. for $C_{16}H_{19}ClN_4NaO_5$ 405.0936; Found 405.0943.

tert-Butyl (S)-(1-benzyl-3-methyl-4-(nitromethyl)-5-oxo-4,5-dihydro-1H-pyrazol-4-yl) carbamate (2l).

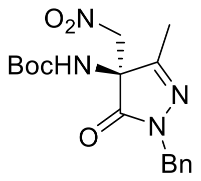

Product **2l** was obtained according to general procedure. Chromatography on a silica gel using Hexane/EtOAc = 4:1 as an eluent afforded compound **2l** as a colorless oil (26 mg, 0.072 mmol, 72% yield). **[α]D** = -25.5 (c = 0.60, $CHCl_3$) for er 79:21. **^{1}H NMR** (500 MHz, $CDCl_3$): δ 7.35 (d, J = 4.4 Hz, 4H), 7.31 (m, 1H), 6.01 (br, 1H), 4.95 (d, J = 15.2 Hz, 1H), 4.77 (d, J = 15.2 Hz, 1H), 4.60 (d, J = 12.2 Hz, 1H), 4.47 (d, J = 12.2 Hz, 1H), 2.00 (s, 3H), 1.43 (s, 9H) ppm. **^{13}C NMR** (126 MHz, $CDCl_3$): δ 170.0, 156.6, 153.5, 135.7, 128.7, 128.1, 127.9, 82.2, 75.9, 48.8, 28.1 (3C), 13.9 ppm. **HPLC:** Chiralpak AD-H column, Hexane/iPrOH 85:15, 1 mL/min, λ = 254 nm, major enantiomer (S) t_r = 7.687 min, minor enantiomer (R) t_r =21.741 min. (er **79:21**). **HRMS** (ESI–TOF) m/z: $[M+H]^+$ Calcd. for $C_{17}H_{23}N_4O_5$ 363.1663; Found 363.1667.

tert-Butyl (S)-(1,3-dimethyl-4-(nitromethyl)-5-oxo-4,5-dihydro-1H-pyrazol-4-yl) carbamate (2m).

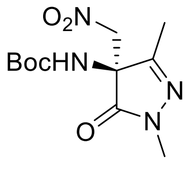

Product **2m** was obtained according to general procedure. Chromatography on a silica gel using Hexane/EtOAc = 4:1 as an eluent afforded compound **2m** as a colorless oil (22 mg, 0.078 mmol, 78% yield). **[α]D** = -25.8 (c = 0.30, $CHCl_3$) for er 80:20. **^{1}H NMR** (500 MHz, $CDCl_3$): δ 5.97 (br, 1H), 4.60 (d, *J* = 12.3 Hz, 1H), 4.48 (d, *J* = 12.2 Hz, 1H), 3.34 (s, 3H), 2.01 (s, 3H), 1.42 (s, 9H) ppm. **^{13}C NMR** (126 MHz, $CDCl_3$): δ 170.2, 156.3, 153.3, 82.2, 75.9, 62.4, 32.0, 28.0 (3C), 13.7 ppm. **HPLC:** Chiralpak AD-H column, Hexane/iPrOH 85:15, 1 mL/min, λ = 254 nm, major enantiomer (S) t_r = 7.018 min, minor enantiomer (R) t_r =13.502 min. (er **80:20**). **HRMS** (ESI–TOF) m/z: $[M+H]^+$ Calcd. for $C_{11}H_{19}N_4O_5$ 287.1350; Found 287.1349.

tert-Butyl (S)-(3-isopropyl-1-methyl-4-(nitromethyl)-5-oxo-4,5-dihydro-1H-pyrazol-4-yl) carbamate (2n).

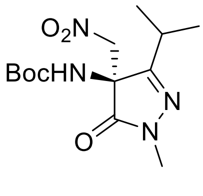

Product **2n** was obtained according to general procedure. Chromatography on a silica gel using Hexane/EtOAc = 4:1 as an eluent afforded compound **2n** as a colorless solid (21 mg, 0.068 mmol, 68% yield). **[α]$_D$** = -22.0 (c = 0.6, $CHCl_3$) for er 77:23. **^{1}H NMR** (500 MHz, $CDCl_3$): δ 6.08 (br, 1H), 4.54 (d, *J* = 12.3 Hz, 1H), 4.47 (d, *J* = 12.3 Hz, 1H), 3.33 (s, 3H), 2.49 (hept, *J* = 6.8 Hz, 1H), 1.39 (s, 9H), 1.20 (d, *J* = 6.8 Hz, 3H), 1.17 (d, *J* = 6.8 Hz, 3H) ppm. **^{13}C NMR** (126 MHz, $CDCl_3$): δ 170.1, 162.9, 153.4, 81.9, 76.2, 62.5, 32.1, 28.3, 28.1 (3C), 21.4, 20.4 ppm. **HPLC:** Chiralpak AD-H column, Hexane/iPrOH 85:15, 1 mL/min, λ = 254 nm, major enantiomer (*S*) t_r = 5.868 min, minor enantiomer (R) t_r =10.047 min. (er **77:23**). **HRMS** (ESI–TOF) m/z: $[M+H]^+$ Calcd. for $C_{13}H_{23}N_4O_5$ 315.1663; Found 315.1665.

(S)-5-(tert-Butyl)-4-hydroxy-4-(nitromethyl)-2-phenyl-2,4-dihydro-3H-pyrazol-3-one (3e).

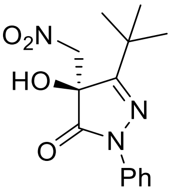

After following the general procedure using catalyst **C1** (6 mg, 0.001 mmol, 0.10 equiv), no aza-Henry product (**2e**) was observed after three days of reaction, presumably due to increased steric hindrance. Instead, racemic product **3e** was isolated as a result of partial hydrolysis of the imine followed by a Henry reaction as a colorless solid (12 mg, 0.040 mmol, 40% yield). **^{1}H NMR** (500 MHz, $CDCl_3$) δ 7.84 (d, *J* = 7.6 Hz, 2H), 7.45 – 7.40 (m, 2H), 7.25 (t, *J* = 7.4 Hz, 1H), 4.99 (d, *J* = 14.0 Hz, 1H), 4.91 (d, *J* = 14.0 Hz, 1H), 3.94 (br, 1H), 1.39 (s, 9H) ppm. **^{13}C NMR** (126 MHz, $CDCl_3$) δ 170.4, 165.4, 137.1, 129.0 (2C), 126.0, 119.1 (2C), 77.3, 77.0, 36.4, 28.6 (3C) ppm. **HRMS** (ESI–TOF) m/z: $[M+Na]^+$ Calcd. for $C_{14}H_{17}N_3NaO_4$ 314.1111; Found 314.1119.

tert-butyl ((S)-3-methyl-4-((S)-1-nitroethyl)-5-oxo-1-phenyl-4,5-dihydro-1H-pyrazol-4-yl) carbamate (4a).

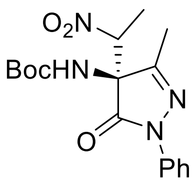

Product **4a** was obtained according to general procedure. Chromatography on a silica gel using Hexane/AcOEt = 4:1 as an eluent afforded compound **4a** (20 mg, 0.054 mmol, 54% yield) as a mixture 64:36 of diastereomers. Colorless solid. **^{1}H NMR** (500 MHz, $CDCl_3$) (diastereomeric mixture) δ 7.86 (d, *J* = 7.8 Hz, 2H), 7.44 – 7.37 (m, 2H), 7.22 (t, *J* = 6.8 Hz, 1H), 6.15-5.99 (br, 1H), 4.80-4.70 (q, *J* = 6.7 Hz, 1H), 2.06 (s, 3H), 1.68-1.48 (d, *J* = 6.8 Hz, 3H), 1.37 (s, 9H) ppm. **^{13}C NMR** (126 MHz, $CDCl_3$): *Mayor diastereomer*: δ 168.9, 161.52, 156.3, 137.4, 129.0 (2C), 125.7, 118.9 (2C), 82.9, 77.2, 67.1, 28.0, 14.0, 12.4 ppm. *Minor diastereomer:* δ 167.9, 161.46, 153.1, 137.6, 128.9 (2C), 125.6, 119.0 (2C), 82.2, 77.2, 65.9, 28.2 (3C), 13.5, 12.2 ppm. **IR (ATR):** 3414, 2974, 2923, 2853, 1710, 1597, 1556, 1487, 1395, 1366, 1252, 1157, 754, 692 cm^{-1}. **HPLC:** Chiralpak AD-H column, Hexane/iPrOH 85:15, 1 mL/min, λ = 254 nm, major enantiomer (S) t_r = 13,667 and 22,408 min, minor enantiomer (R) t_r =57.167 min. (er: **$86:14_{major}$, $85:15_{minor}$**). **HRMS** (ESI–TOF) m/z: $[M+Na]^+$ Calcd. for $C_{17}H_{22}N_4NaO_5$ 385.1482; Found 385.1486.

Synthesis of monomer derived from isatin (M1)

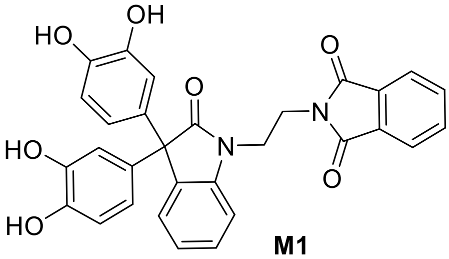

M1

In a dry Schlenk tube, ~~the catalyst~~ $[CPh_3][B(C_6F_5)_4]$ (3.0 mol %) was introduced and purged with N_2. Dried 1,2-dichloroethane (0.2 mL) was then added, followed by Et_3SiH (5 mol%) to generate $[Et_3Si][B(C_6F_5)_4]$ *in situ*. Then, dried 1,2-dichloroethane (0.2 mL) solvent was added to the tube. Further, Et_3SiH (5 mol %) was added to this solution for in-situ generation of $[Et_3Si][B(C_6F_5)_4]$. Upon addition of Et_3SiH, the reaction mixture changed color from yellow to colorless, indicating formation of the active catalyst. Subsequently, N-protected isatin[2] (1.0 g, 3.1 mmol) and catechol (0.75 g, 6.9 mmol, 2.2 equiv.) dissolved in DCE (5 mL) were sequentially added to the mixture. The resulting solution was stirred at 60 °C in an oil bath for 20 h (TLC). Upon completion, a solid precipitate was formed. The solid was collected by filtration and washed with DCM. The crude product was purified by column chromatography (Hexane/EtOAc = 1:2), affording a red solid (1.42 g, 2.8 mmol, 90 % yield). **^{1}H NMR** (400 MHz, DMSO-d6) δ 7.78 – 7.70 (m, 4H), 7.29 (t,

[2] Diaz, P.; Xu, J.; Astruc-Diaz, F.; Hao-Min, P.; Brown, D. L.; Naguib, M. Design and Synthesis of a Novel Series of N-Alkyl Isatin Acylhydrazone Derivatives that Act as Selective Cannabinoid Receptor 2 Agonists for the Treatment of Neuropathic Pain *J. Med. Chem.* **2008**, *51*, 4932-4947.

J = 7.6 Hz, 1H), 7.19 (d, *J* = 7.8 Hz, 1H), 7.12 (d, *J* = 7.3 Hz, 1H), 7.05 (t, *J* = 7.7 Hz, 1H), 6.47 (d, *J* = 2.3 Hz, 2H), 6.44 (d, *J* = 8.3 Hz, 2H), 6.14 (dd, *J* = 8.3, 2.3 Hz, 2H), 4.01 (t, *J* = 5.8 Hz, 2H), 3.85 (t, *J* = 5.6 Hz, 2H) ppm. **^{13}C NMR** (101 MHz, DMSO-d6) δ 177.9, 167.9, 145.2, 144.8, 141.9, 134.6, 133.9, 132.9, 131.8, 128.3, 126.1, 123.4, 122.8, 119.1, 116.4, 115.2, 109.2, 60.7, 60.2, 21.2, 14.5 ppm. IR (ATR): 3489, 3202, 1771, 1711, 1692, 1663, 1597, 1518, 1389, 1241, 1187, 1115, 872, 758, 724 cm^{-1}. **HRMS** (ESI-QTOF) m/z: [M+Na]$^+$ Calcd. for $C_{30}H_{22}N_2NaO_7$ 545.1319; Found 545.1331.

Transformations of adduct 2a in several enantiopure compounds 5a-10a

(S)-4-Amino-5-methyl-4-(nitromethyl)-2-phenyl-2,4-dihydro-3H-pyrazol-3-one (5a).

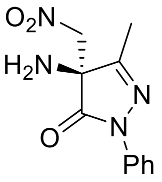

To the compound **2a** (170 mg, 0.49 mmol), HCl in dioxane (2 mL, 4.0 M) was added. The reaction mixture was stirred at room temperature for 1h. After completion, the solvent was evaporated under reduced pressure and the residue redissolved in DCM (5 mL). The solution was basified with saturated aqueous $NaHCO_3$. The organic layer was separated, dried over anhydrous $MgSO_4$, filtered, and concentrated under reduced pressure to afford compound **5a** as a colorless oil (110 mg, 0.44 mmol, 91 %). **[α]$_D$** = 45.2 (c = 0.5, $CHCl_3$) for er 98:2. **^{1}H NMR** (500 MHz, $CDCl_3$): δ 7.93 (m, 2H), 7.49 (m, 2H), 7.26 (m, 1H), 4.80 (d, *J* = 13.5 Hz, 1H), 4.69 (d, *J* = 13.6 Hz, 1H), 2.26 (s, 3H), 1.82 (br, 2H) ppm. **^{13}C NMR** (126 MHz, $CDCl_3$): δ 171.8, 158.7, 137.4, 129.0 (2C), 125.7, 119.0 (2C), 76.8, 63.1, 13.4 ppm. **HPLC:** Chiralpak AD-H column, Hexane/iPrOH 85:15, 1 mL/min, λ = 254 nm, major enantiomer (S) t_r = 12.56 min, minor enantiomer (R) t_r =15.112 min. (er **98:2**). **HRMS** (ESI–TOF) m/z: [M+Na]$^+$ Calcd. for $C_{11}H_{12}N_4NaO_3$ 271.0802; Found 271.0800.

tert-butyl (3-methyl-5-oxo-1-phenyl-4,5-dihydro-1H-pyrazol-4-yl)carbamate (6a).

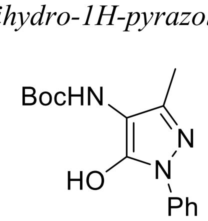

To a cooled solution of compound **5a** (360 mg, 1.03 mmol) in methanol (3 mL), maintained at 0 °C, were successively added $NiCl_2·6H_2O$ (26 mg, 0.103 mmol) and $NaBH_4$ (19.5 mg, 0.919 mmol). The resulting mixture was stirred at 0 °C for 30-40 minutes to ensure complete reduction.

Subsequently, the solvent evaporated, and the crude product was purified by flash chromatography using hexane/EtOAc 4:1 to afford to **6a** as a yellowish solid (28 mg, 0.098 mmol, 95 % yield). ^{1}**H NMR** (400 MHz, $CDCl_3$) δ 10.25 (br, 1H), 7.73 (d, *J* = 7.5 Hz, 2H), 7.40 (t, *J* = 7.7 Hz, 2H), 7.22 (t, *J* = 7.4 Hz, 1H), 6.07 (br, 1H), 2.19 (s, 3H), 1.52 (s, 9H) ppm.

(S)-1-(3-Methyl-4-(nitromethyl)-5-oxo-1-phenyl-4,5-dihydro-1H-pyrazol-4-yl)-3-phenylurea (7a).

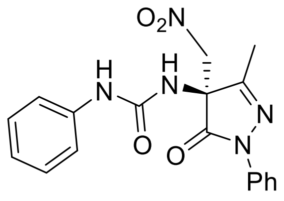

A solution of phenyl isocyanate (11 µL, 0.1 mmol, 1.0 equiv) and **5a** (25 mg, 0.1 mmol) in DCM (2 mL) was stirred at room temperature for 12h. Upon completion, the solvent was removed under reduced pressure and the residue was purified by silica gel flash chromatography (DCM/Et_2O = 50:1) to afford **7a** as a white solid (27 mg, 0.073 mmol, 73% yield). **[α]$_D$** = -80.3 (c = 0.3, $CHCl_3$) for er 98:2. ^{1}**H NMR** (500 MHz, Acetone-d6): δ 8.64 (br, 1H), 7.92 (dq, *J* = 7.7, 1.1 Hz, 2H), 7.44 (m, 4H), 7.23 (m, 3H), 7.11 (br, 1H), 6.98 (tt, *J* = 7.3, 1.2 Hz, 1H), 5.21 (d, *J* = 12.6 Hz, 1H), 5.04 (dd, *J* = 12.5, 0.6 Hz, 1H), 2.18 (s, 3H) ppm. ^{13}**C NMR** (126 MHz, Acetone-d6): δ 169.5, 157.2, 153.3, 139.3, 138.5, 128.71 (2C), 128.70 (2C), 124.9, 122.5, 118.43 (2C), 118.41 (2C), 76.0, 63.9, 12.9 ppm. **HPLC:** Chiralpak AD-H column, Hexane/iPrOH 85:15, 1 mL/min, λ = 254 nm, major enantiomer (S) t_r = 15.778 min, minor enantiomer (R) t_r =9.989 min. (er **98:2**). **HRMS** (ESI–TOF) m/z: $[M+H]^+$ Calcd. for $C_{18}H_{18}N_5O_4$ 368.1353; Found 368.1363.

(S)-1-(4-bromophenyl)-3-(3-methyl-4-(nitromethyl)-5-oxo-1-phenyl-4,5-dihydro-1H-pyrazol-4-yl)urea (8a).

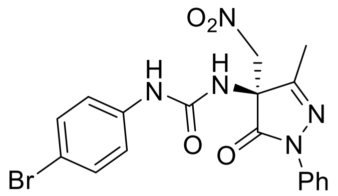

A mixture of p-bromo phenyl isocyanate (21 µL, 0.11 mmol, 1.1 equiv) and **5a** (25 mg, 0.1 mmol) in DCM (2 mL) was stirred for 12h at room temperature. After that, the solvent was removed under reduced pressure and the residue was purified by silica gel flash chromatography (Hexane/AcOEt = 3:1) to afford **8a** as a white solid (33 mg, 0.068 mmol, 68% yield). **[α]$_D$** = -58.3 (c = 0.54, $CHCl_3$) for er 97:3. ^{1}**H NMR** (500 MHz, Acetone-d6): δ 8.78 (br, 1H), 7.91 (d, *J* = 7.6 Hz, 2H), 7.47 – 7.43 (m, 2H), 7.40 (m, 4H), 7.23 (t, *J* = 7.5 Hz, 1H), 7.18 (br, 1H), 5.21 (d, *J* =

12.5 Hz, 1H), 5.05 (d, *J* = 12.7 Hz, 1H), 2.18 (s, 3H) ppm. **^{13}C NMR** (126 MHz, Acetone-d6): δ 169.3, 157.0, 153.2, 138.7, 138.5, 131.6 (2C), 128.7 (2C), 124.9, 120.2 (2C), 118.4 (2C), 114.2, 75.9, 63.8, 12.9 ppm. **HPLC:** Chiralpak AD-H column, Hexane/iPrOH 85:15, 1 mL/min, λ = 254 nm, major enantiomer (S) t_r = 26.070 min, minor enantiomer (R) t_r =15.420 min. (er **97:3**). **HRMS** (ESI–TOF) m/z: $[M+H]^+$ Calcd. for $C_{18}H_{16}BrN_5NaO_4$ 468.0278; Found 468.0290.

(S)-1-(3-Methyl-4-(nitromethyl)-5-oxo-1-phenyl-4,5-dihydro-1H-pyrazol-4-yl)-3-phenylthiourea (9a).

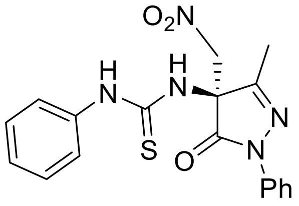

A mixture of phenyl isothiocyanate (36 μL, 0.3 mmol, 3.0 equiv) and **5a** (25 mg, 0.1 mmol) in acetonitrile (1 mL) was stirred at room temperature for 3 days. After completion, the solvent was removed under reduced pressure and the residue was purified by silica gel flash chromatography (hexane/EtOAc = 3:1) to afford **9a** as a brown solid (17 mg, 0.044 mmol, 44% yield). **[α]D** = -157.4 (c = 0.38, $CHCl_3$) for er nd. **^{1}H NMR** (500 MHz, Acetone-d6): δ 8.42 (br, 1H), 8.03 (m, 2H), 7.49 (m, 5H), 7.37 (m, 2H), 7.25 (m, 1H), 6.46 (br, 1H), 5.49 (d, J = 15.8 Hz, 1H), 5.43 (d, J = 15.8 Hz, 1H), 1.72 (s, 3H) ppm. **^{13}C NMR** (126 MHz, Acetone-d6): δ 181.3, 166.8, 138.2, 136.1, 131.1 (2C), 128.9 (2C), 128.5 (2C), 128.4, 125.1 (2C), 118.3, 72.6, 68.5, 17.1 ppm. **HRMS** (ESI–TOF) m/z: $[M+H]^+$ Calcd. for $C_{18}H_{18}N_5O_3S$ 384.1125; Found 384.1127.

(S)-N-(3-methyl-4-(nitromethyl)-5-oxo-1-phenyl-4,5-dihydro-1H-pyrazol-4-yl) acetamide (10a)

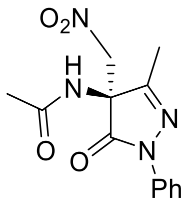

Acetyl chloride (7 μL, 0.1 mmol, 1.0 equiv) was added to a mixture of triethylamine (14 μL, 0.1 mmol, 1.0 equiv) and **5a** (25 mg, 0.1 mmol) in diethyl ether (1 mL). The reaction mixture was stirred at room temperature for 12h. After completion, the solvent was removed under reduced pressure. The residue was redissolved in DCM (5 mL) and washed with water (5 mL). The aqueous phase was extracted three times with dichloromethane, and the combined organic layers were dried over anhydrous $MgSO_4$, filtered and concentrated under reduced pressure. The crude product was purified by silica gel flash chromatography (Hexane/EtOAc = 1:1) to afford **10a** as a brown solid (9 mg, 0.031 mmol, 31% yield). **[α]D** = -28.1 (c = 0.16, $CHCl_3$) for er 98:2. **^{1}H**

NMR (500 MHz, $CDCl_3$): δ 7.83 (m, 2H), 7.42 (m, 2H), 7.24 (m, 1H), 7.06 (br, 1H), 4.73 (d, J = 12.0 Hz, 1H), 4.61 (d, J = 12.0 Hz, 1H), 2.11 (s, 3H), 2.07 (s, 3H) ppm. **^{13}C NMR** (126 MHz, $CDCl_3$): δ 169.7, 167.8, 155.6, 137.4, 129.0 (2C), 126.0, 119.4 (2C), 75.8, 63.6, 22.3, 13.9 ppm. **HPLC:** Chiralpak AD-H column, Hexane/iPrOH 85:15, 1 mL/min, λ = 254 nm, major enantiomer (S) t_r = 12.734 min, minor enantiomer (R) t_r =6.379 min. (er **98:2**). **HRMS** (ESI–TOF) m/z: $[M+H]^+$ Calcd. for $C_{13}H_{15}N_4O_4$ 291.1088; Found 291.1082.

Continuous-flow device used in this work

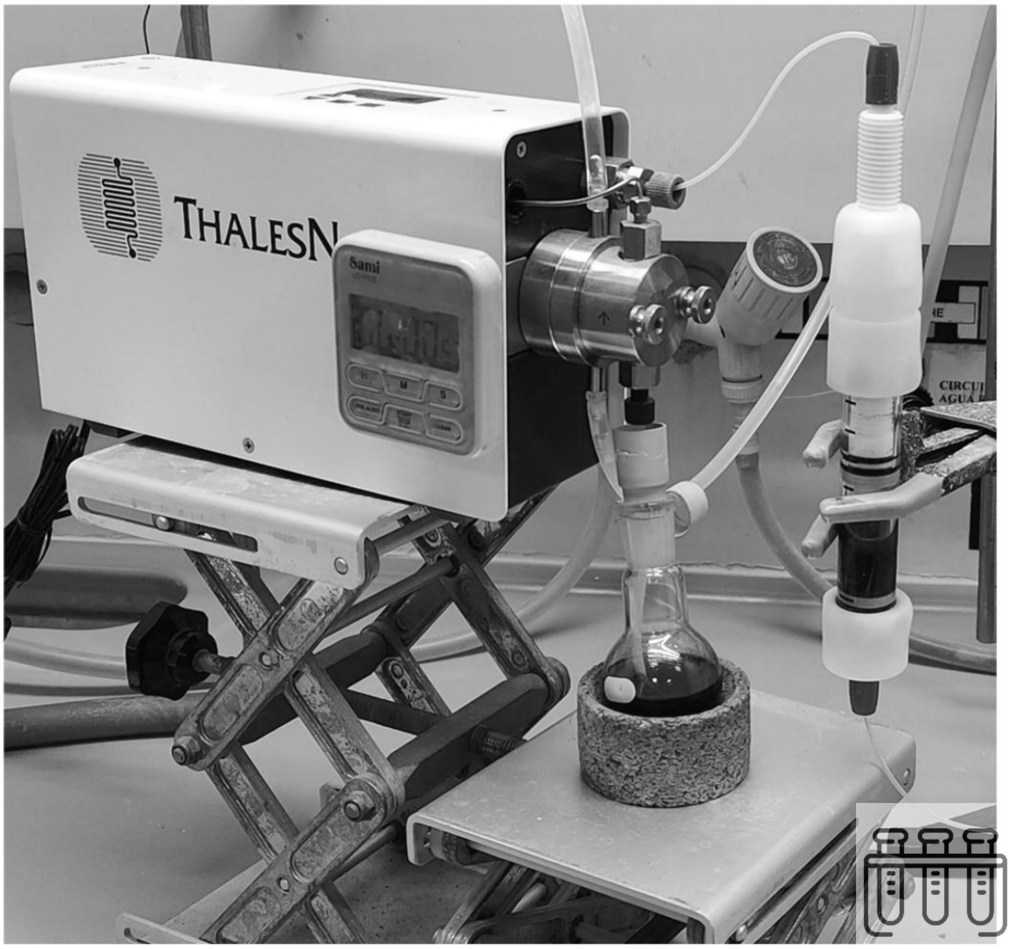

4. NMR Spectra of New Compounds

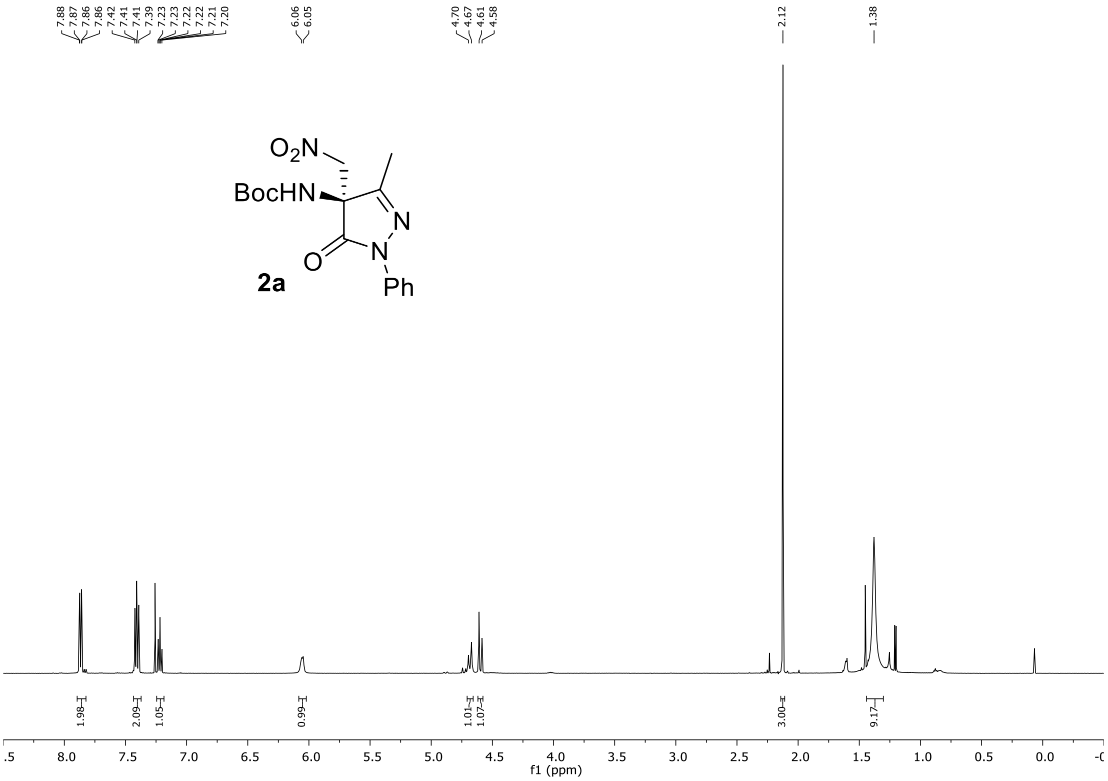

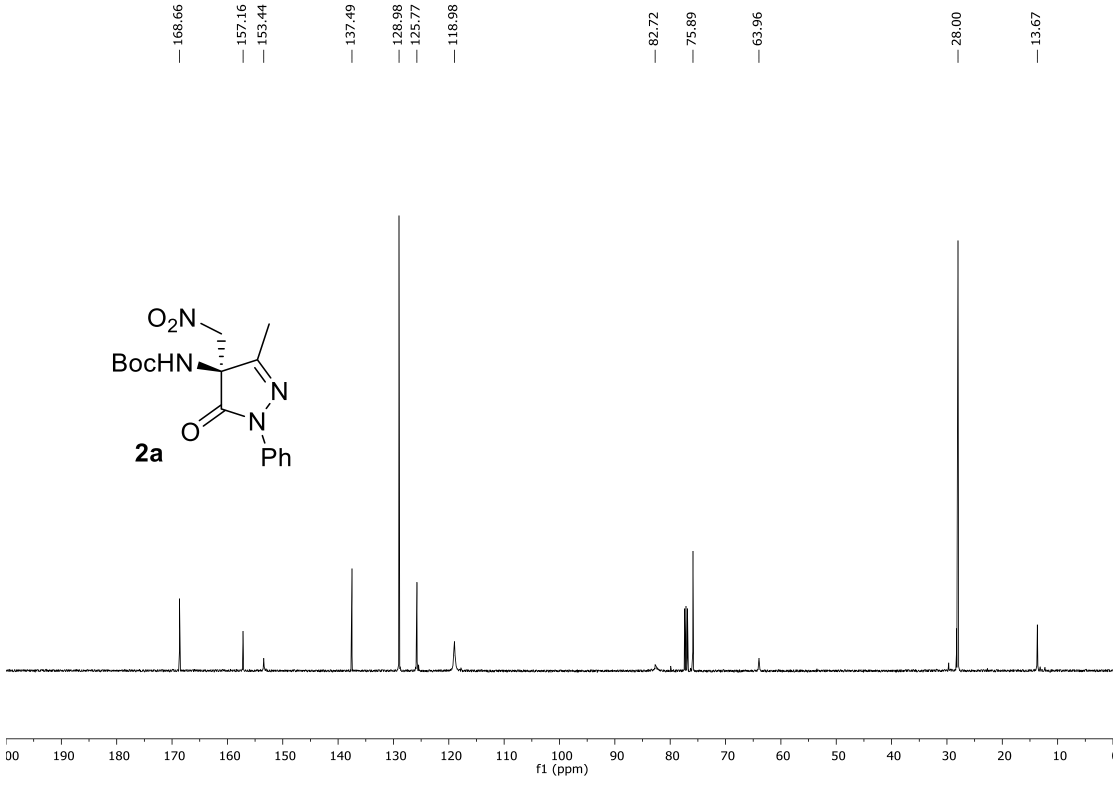

Figure S2. ^{1}H and ^{13}C NMR of **2a** ($CDCl_3$)

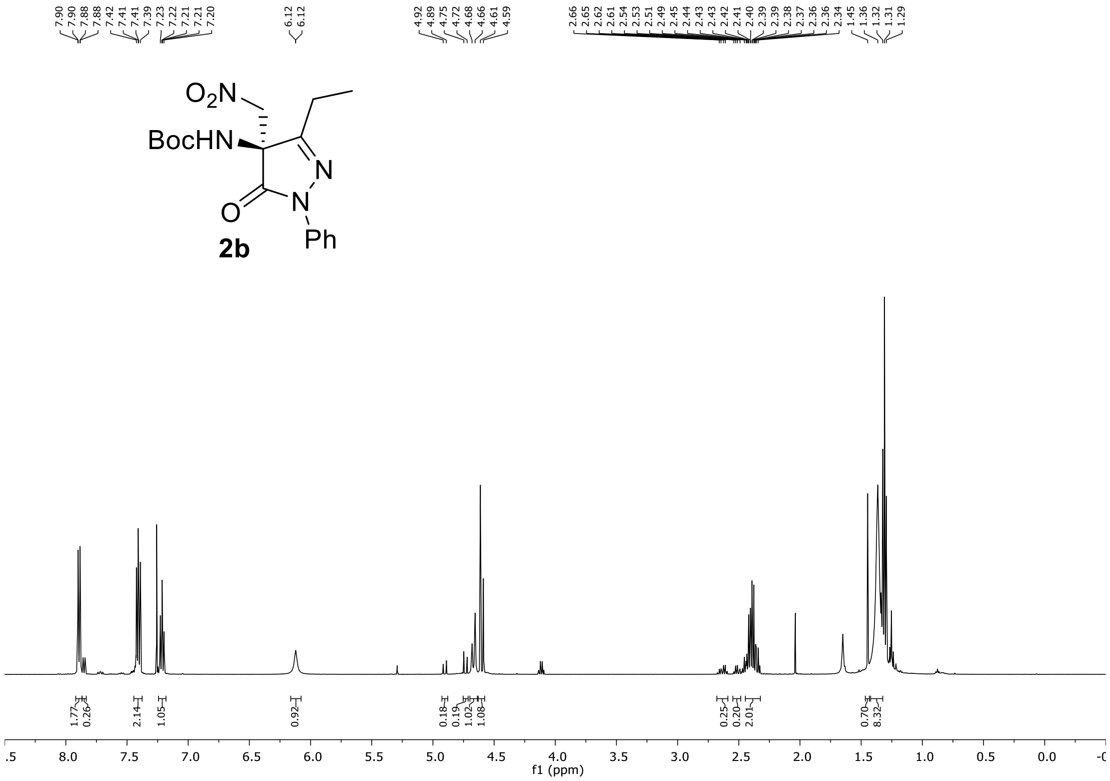

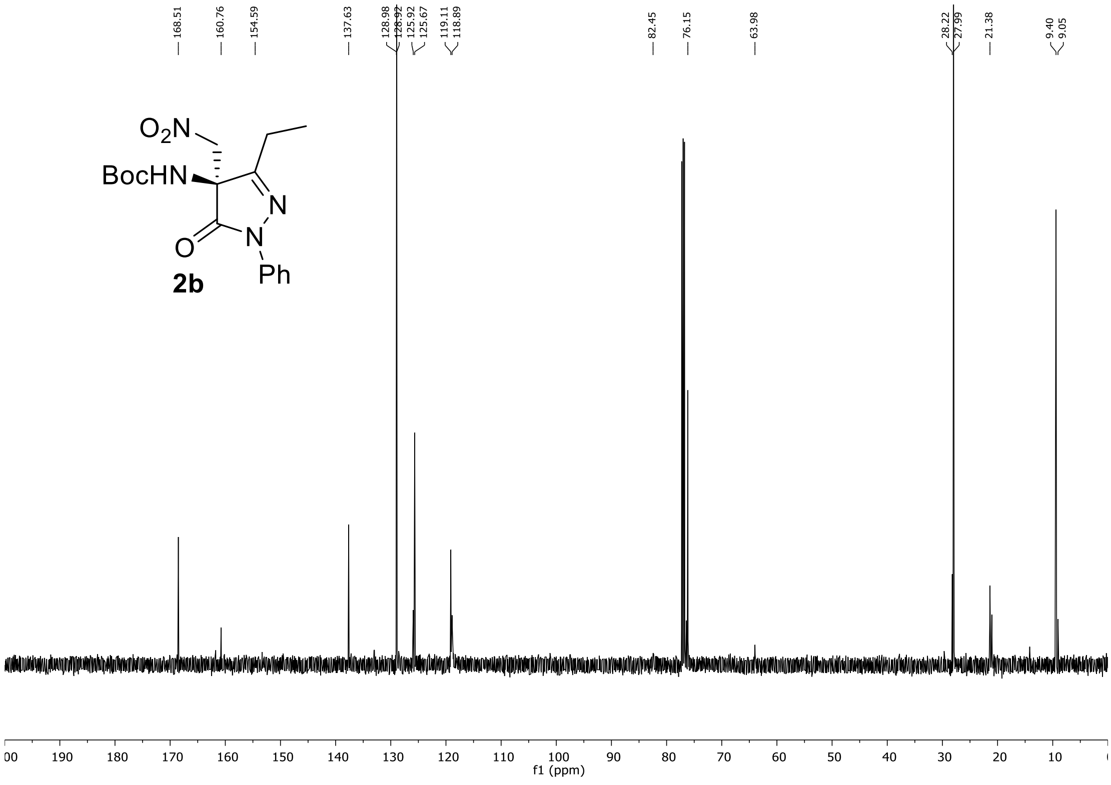

Figure S3. ^{1}H and ^{13}C NMR of **2b** ($CDCl_3$)

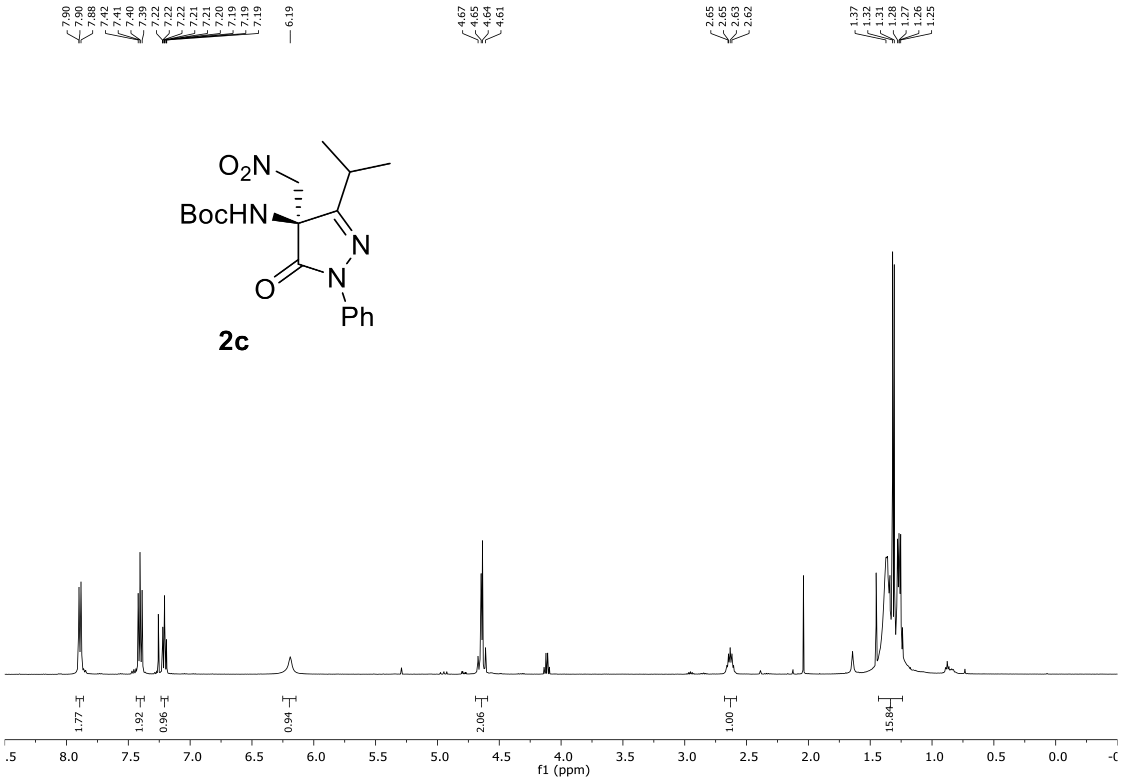

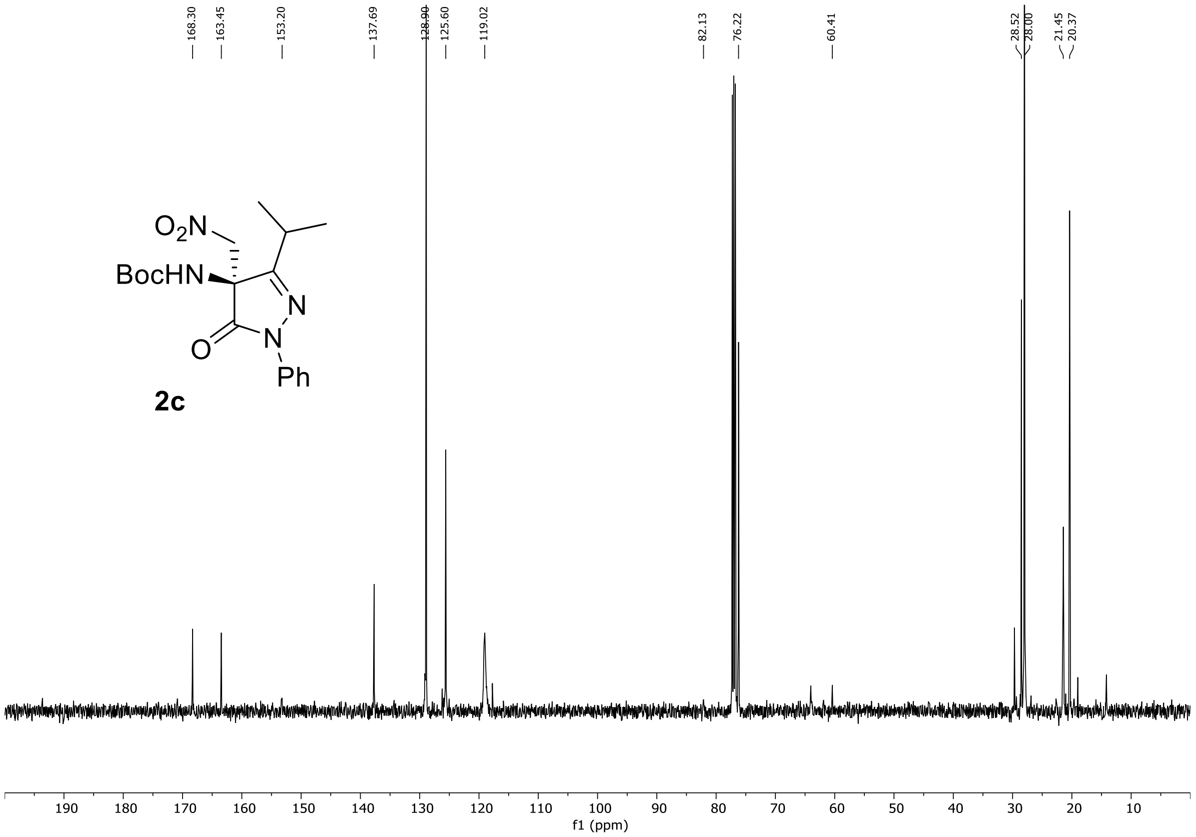

Figure S4. ^{1}H and ^{13}C NMR of **2c** ($CDCl_3$)

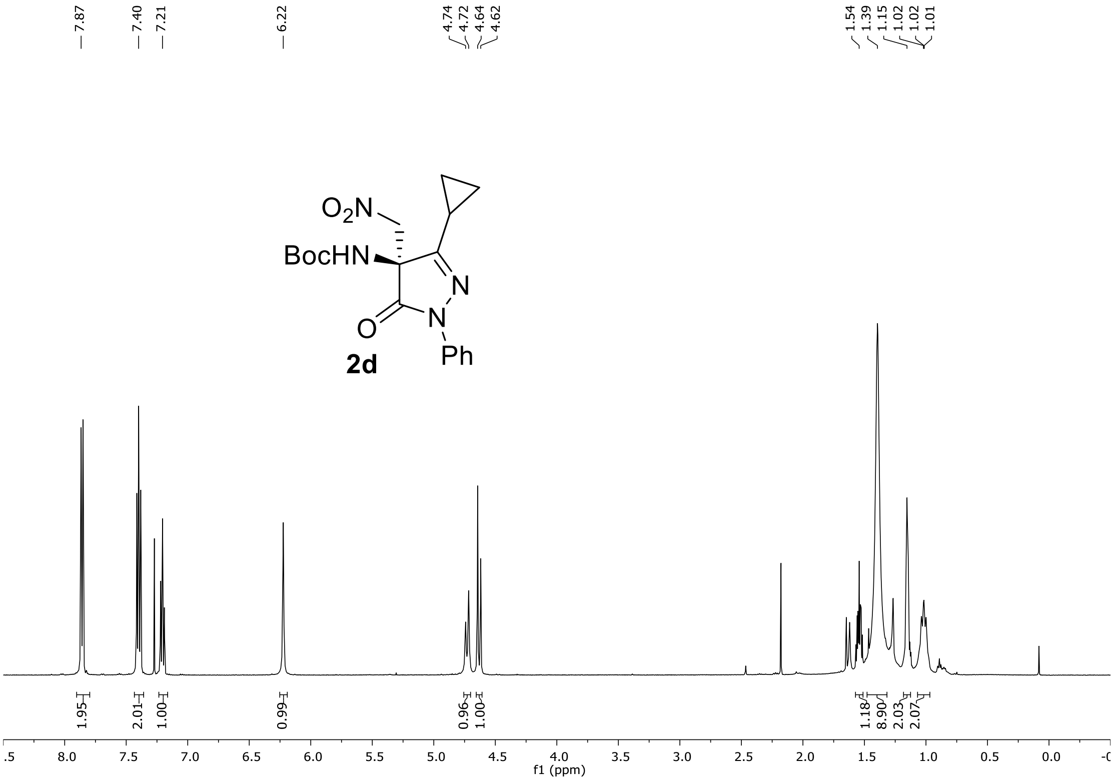

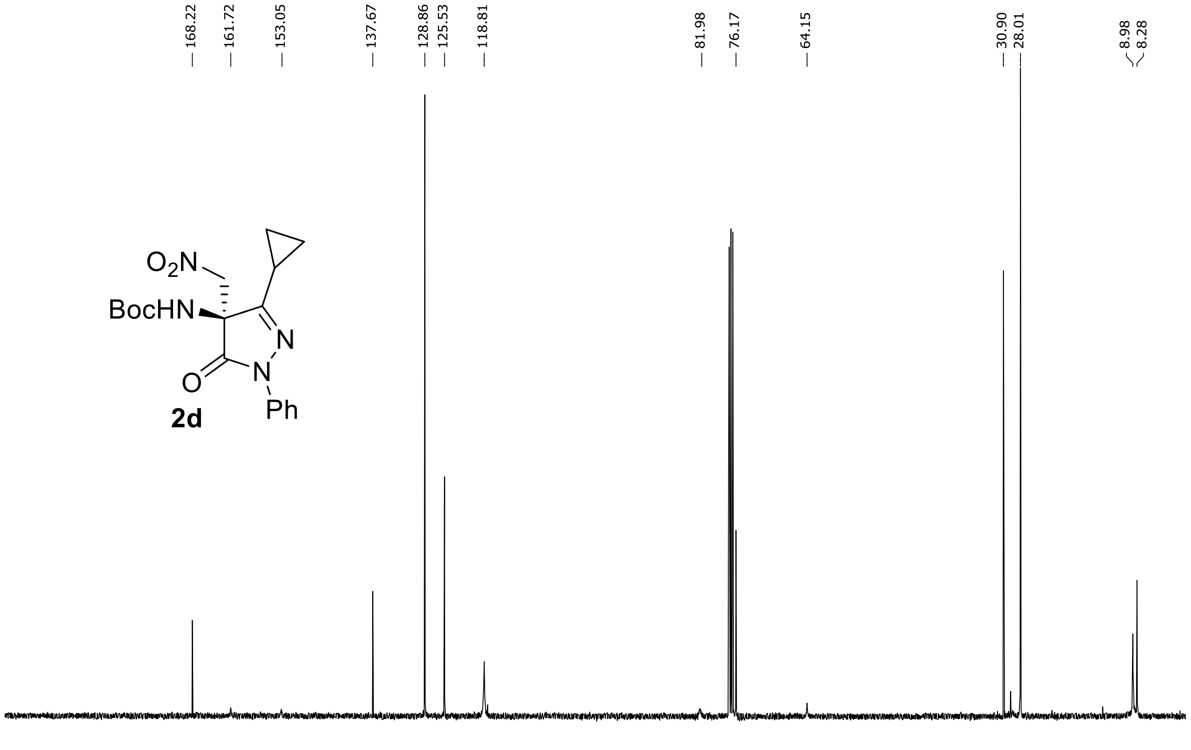

Figure S5. ^{1}H and ^{13}C NMR of **2d** ($CDCl_3$)

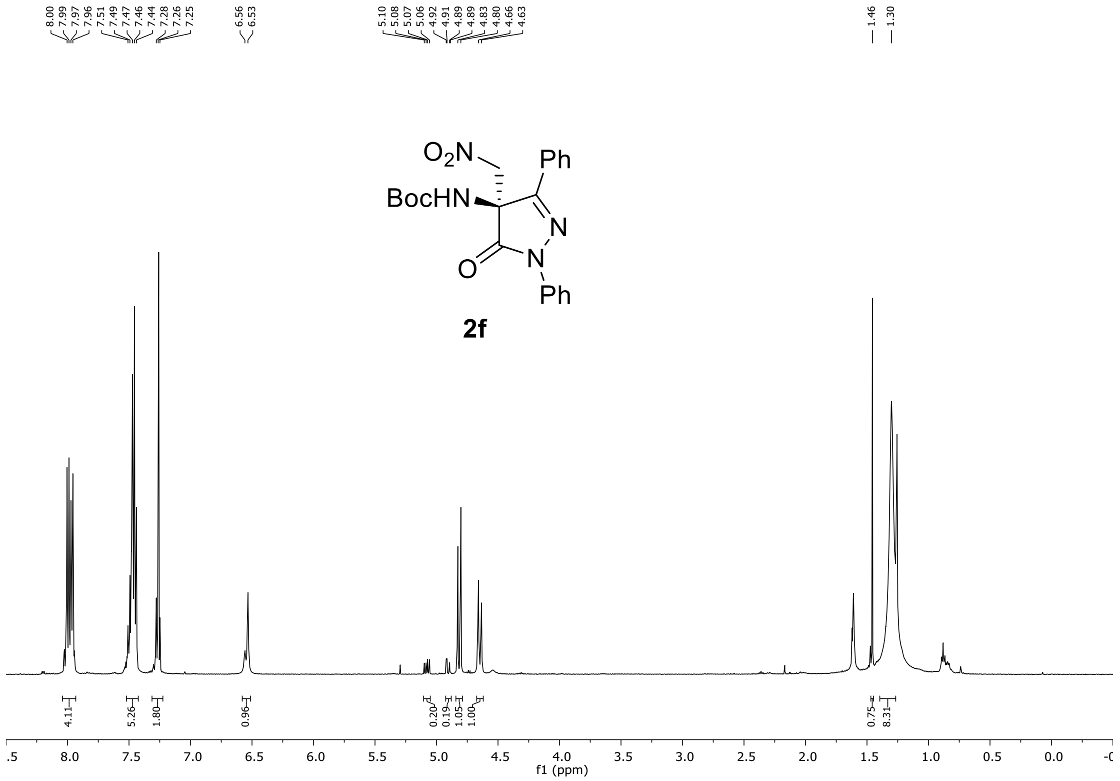

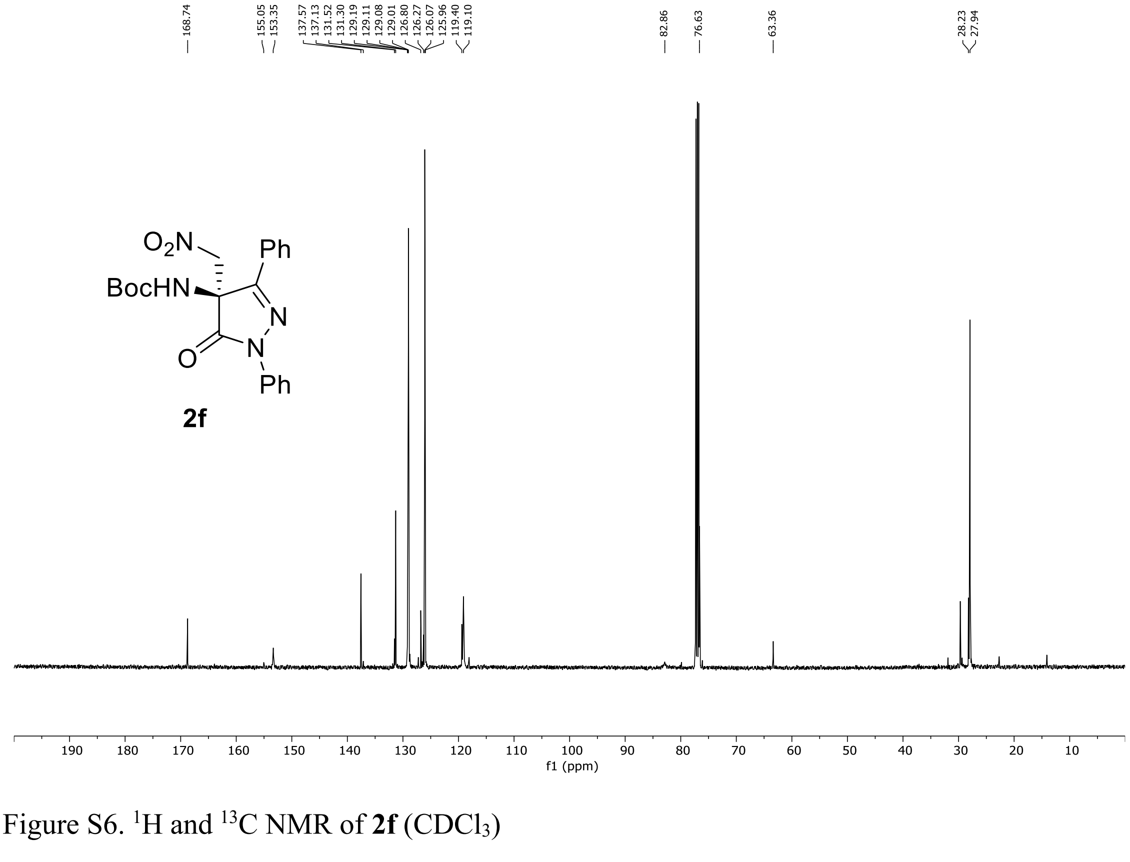

Figure S6. ^{1}H and ^{13}C NMR of **2f** ($CDCl_3$)

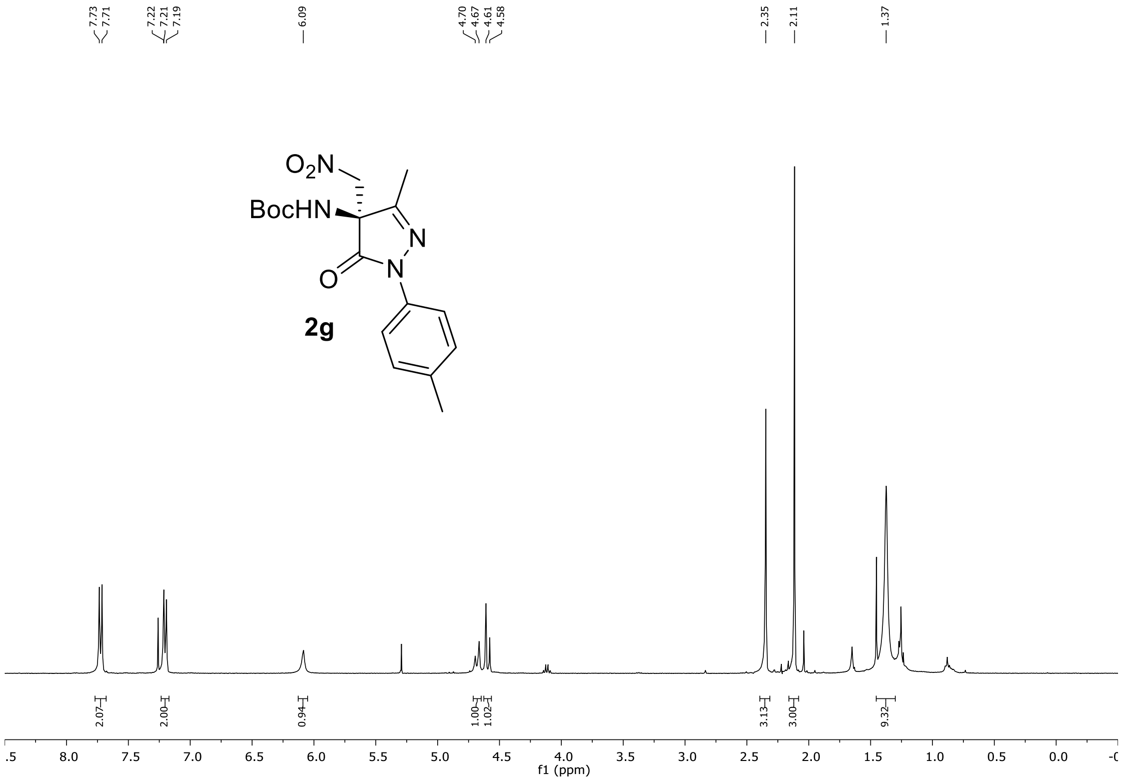

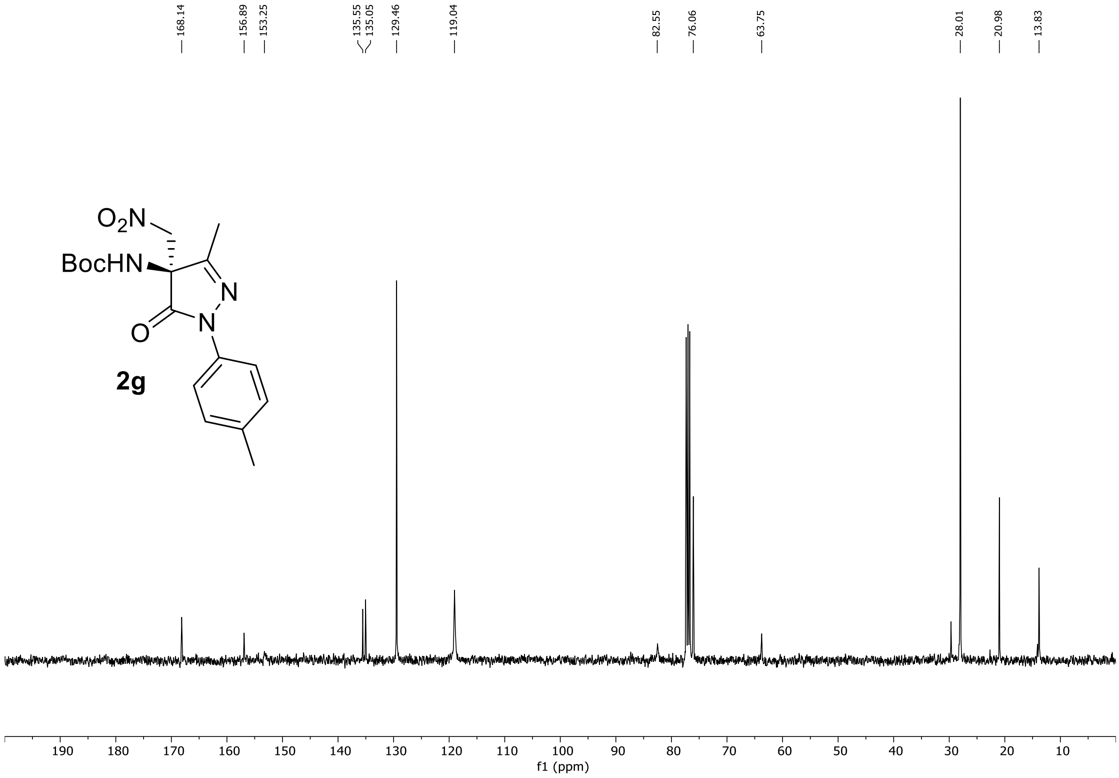

Figure S7. ^{1}H and ^{13}C NMR of **2g** ($CDCl_3$)

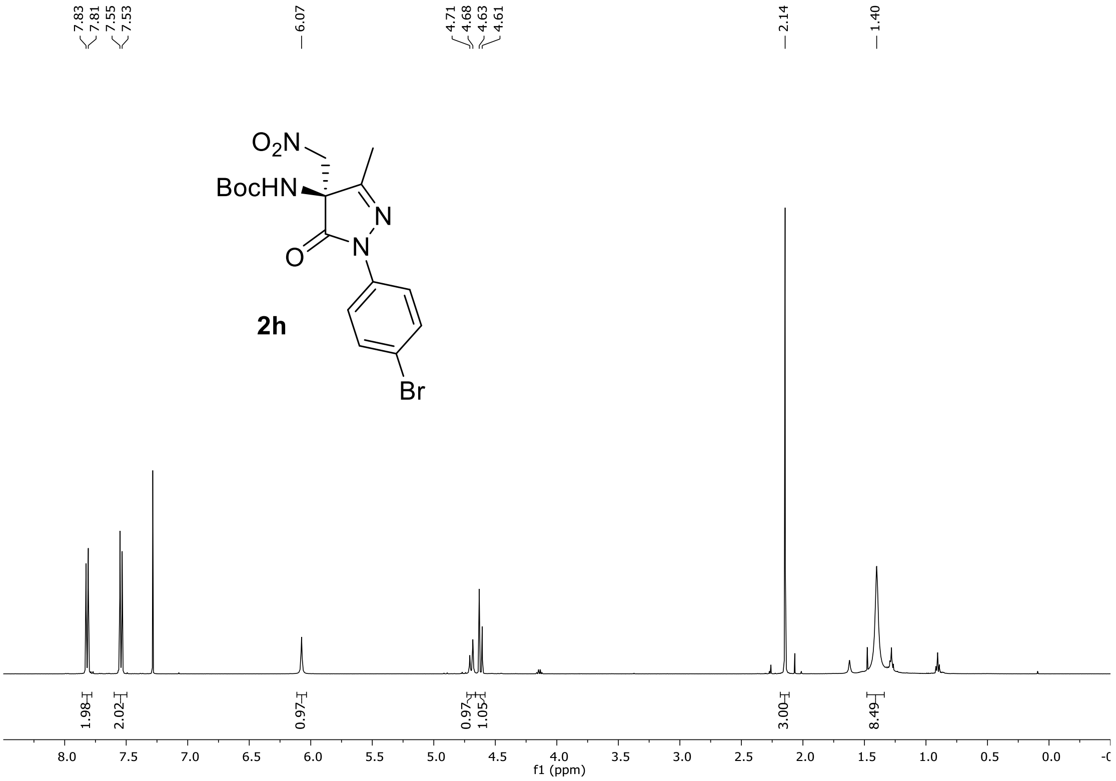

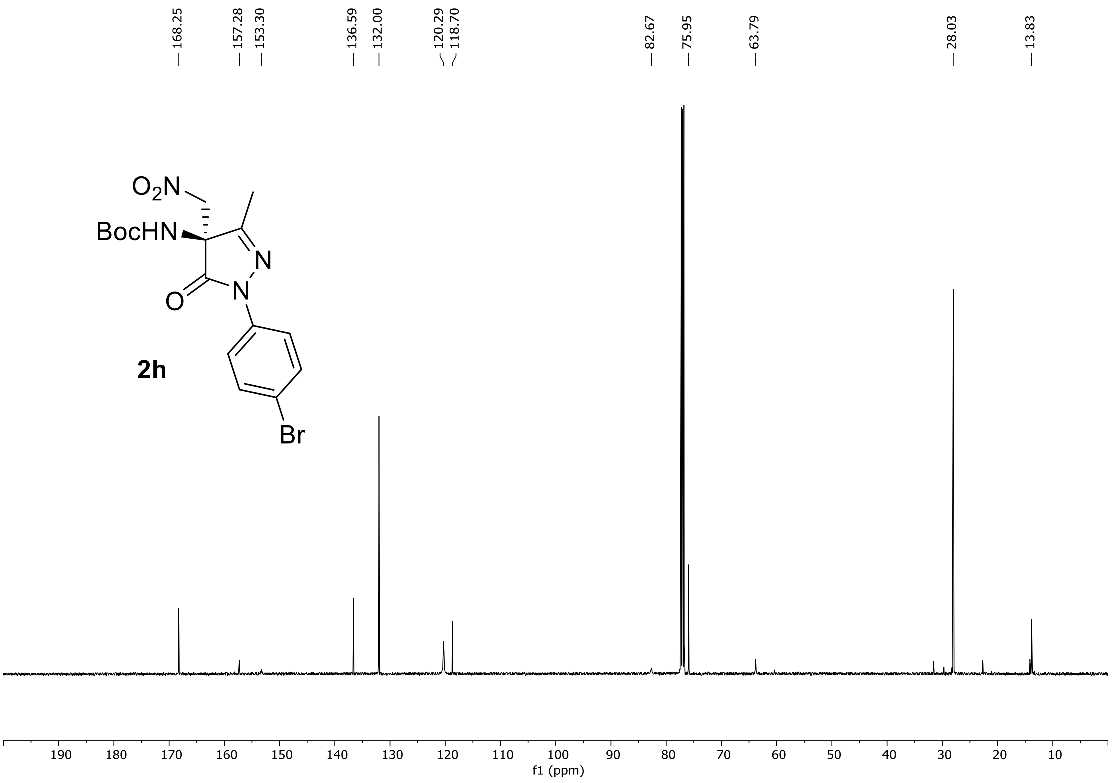

Figure S8. ^{1}H and ^{13}C NMR of **2h** ($CDCl_3$)

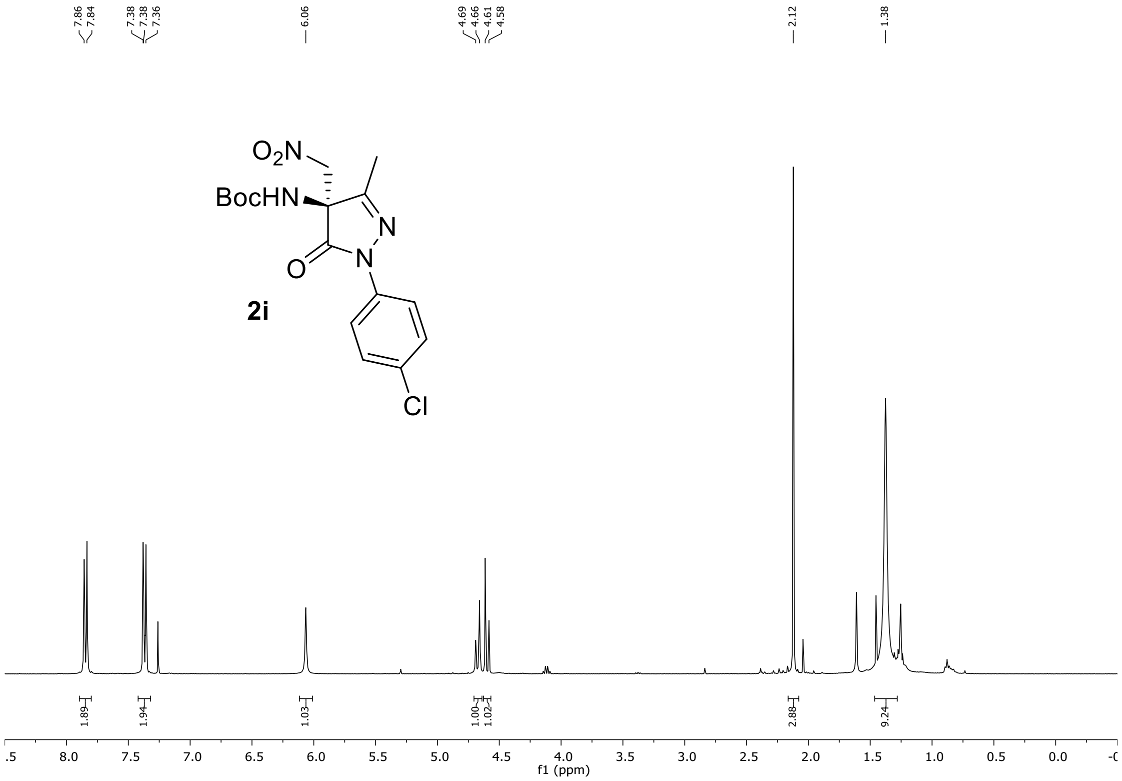

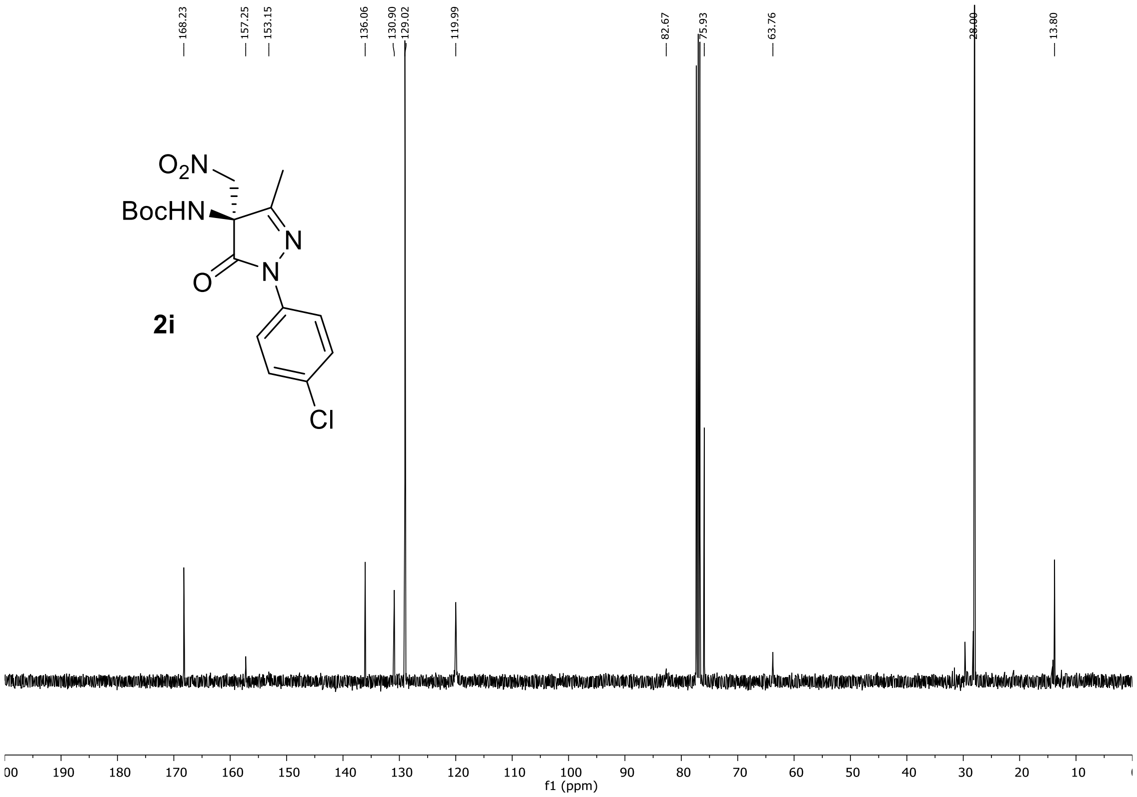

Figure S9. ^{1}H and ^{13}C NMR of **2i** ($CDCl_3$)

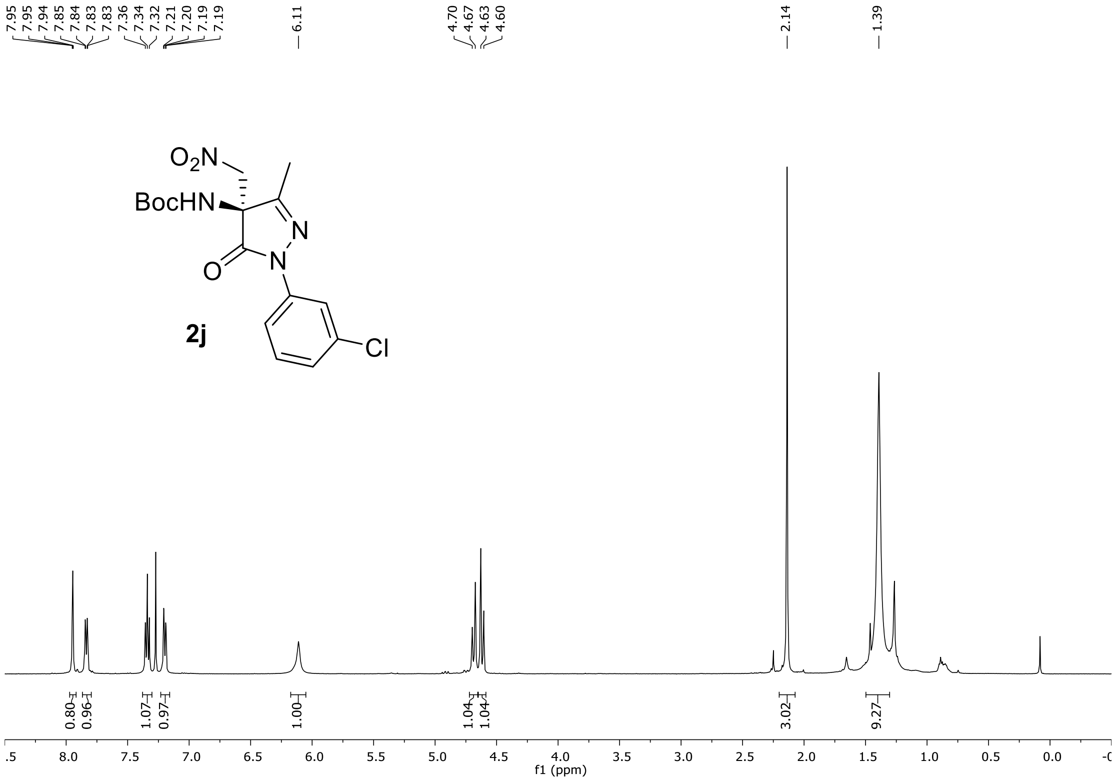

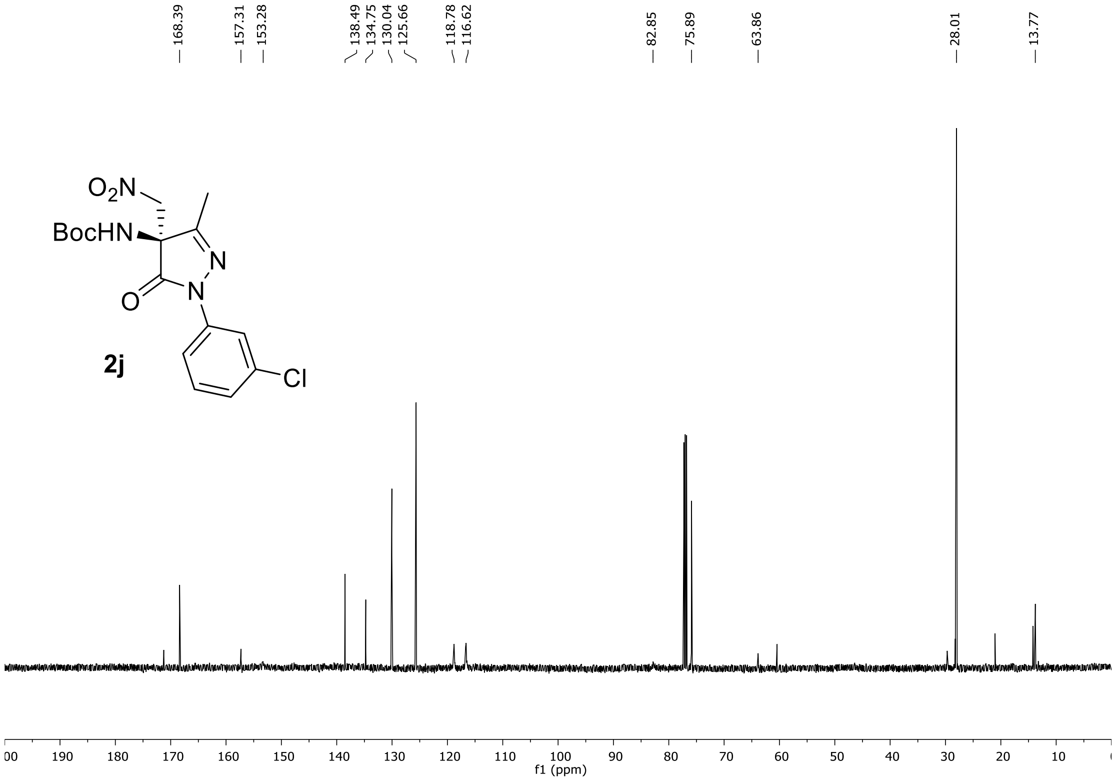

Figure S10. ^{1}H and ^{13}C NMR of **2j** ($CDCl_3$)

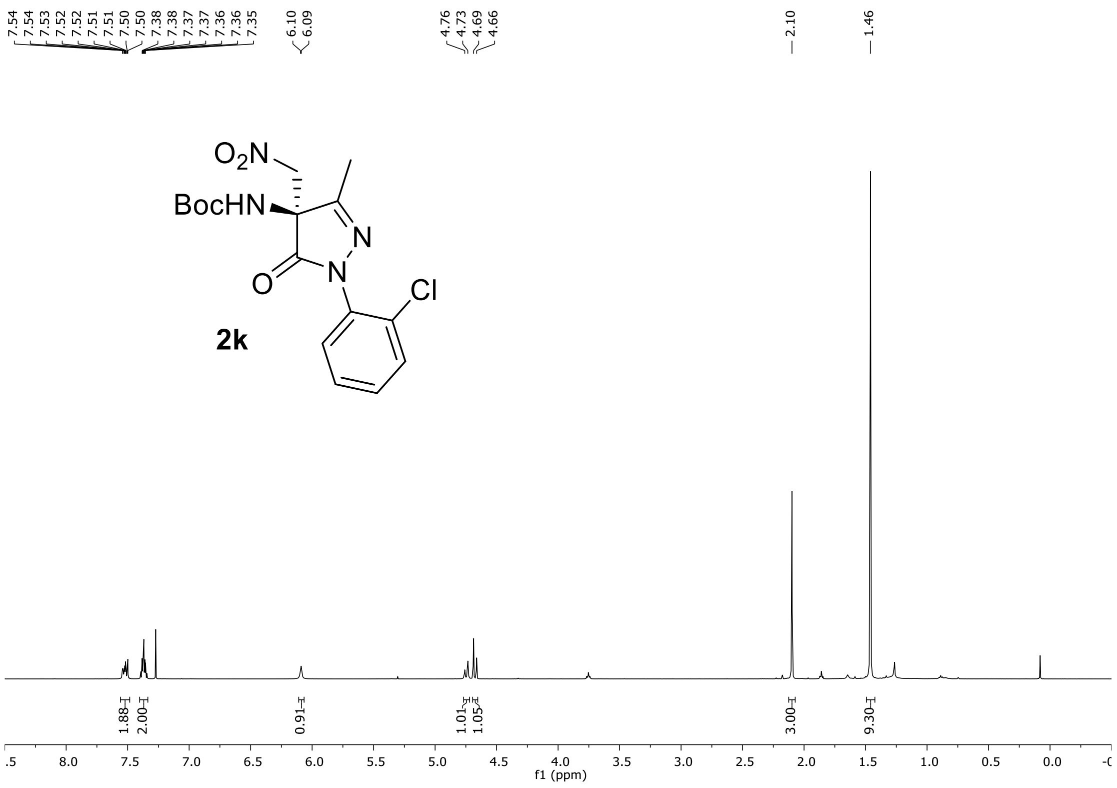

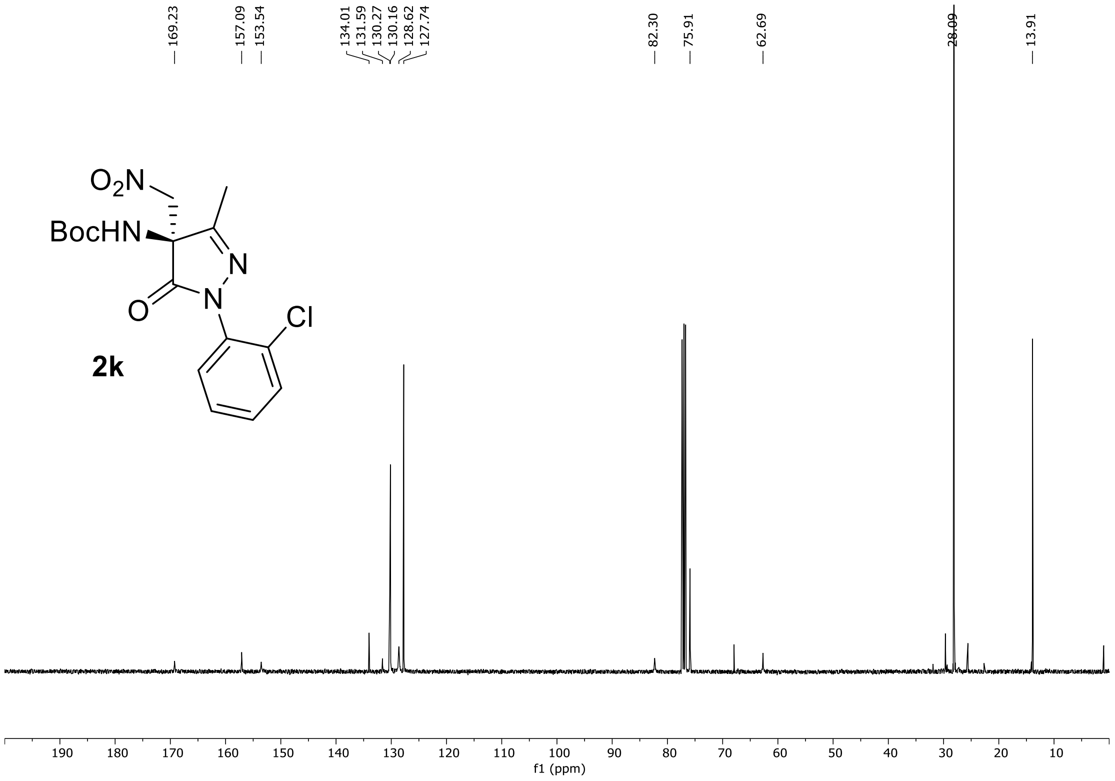

Figure S11. ^{1}H and ^{13}C NMR of **2k** ($CDCl_3$)

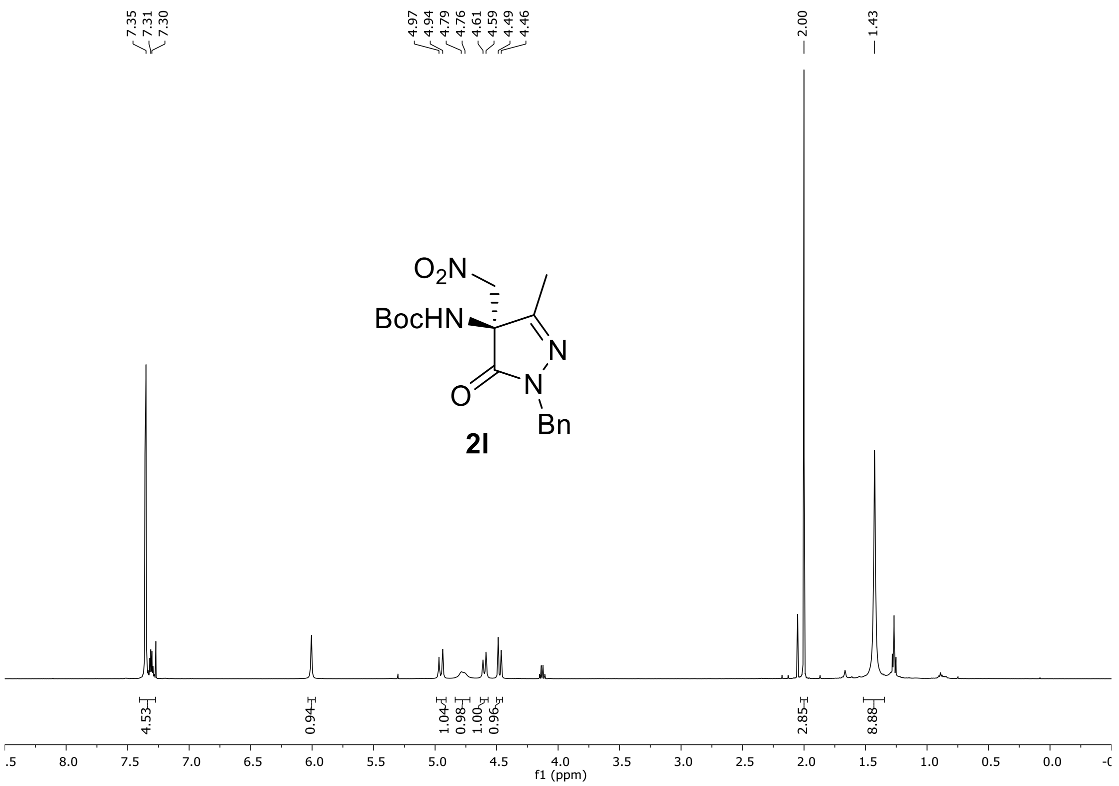

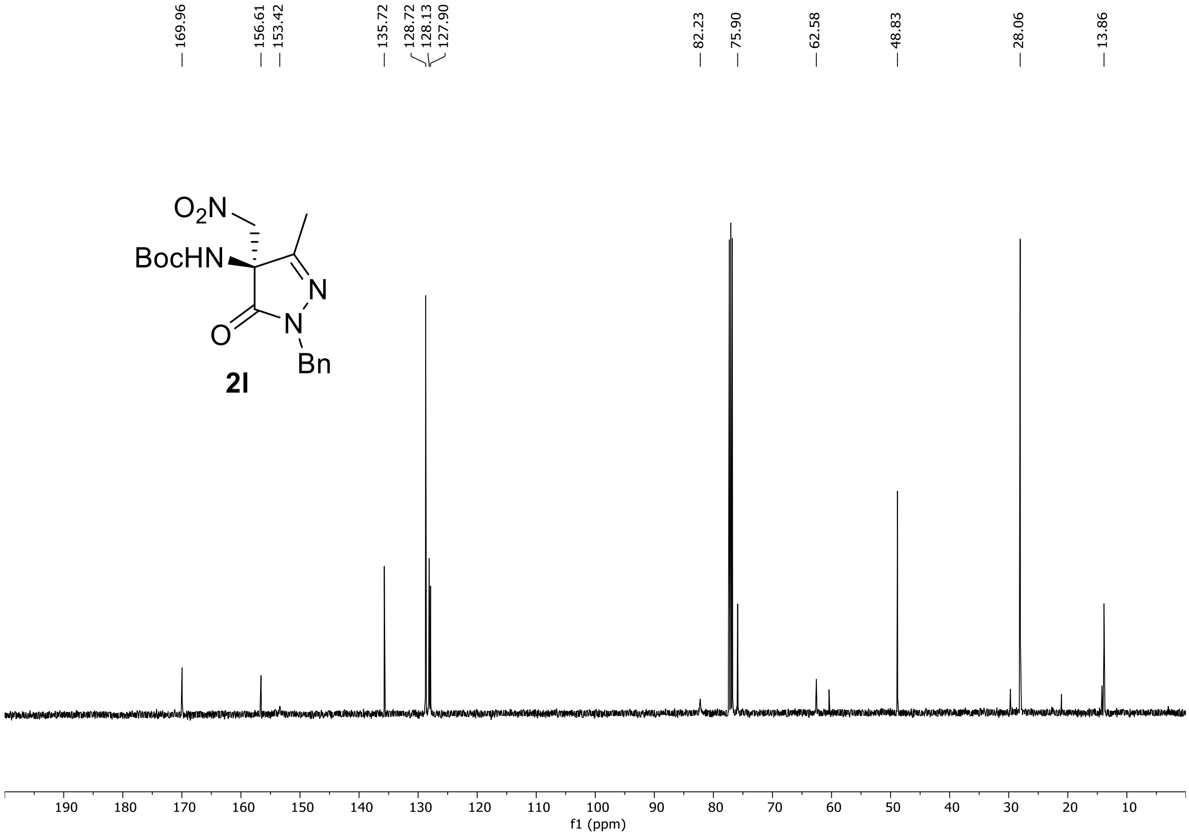

Figure S12. ^{1}H and ^{13}C NMR of **2l** ($CDCl_3$)

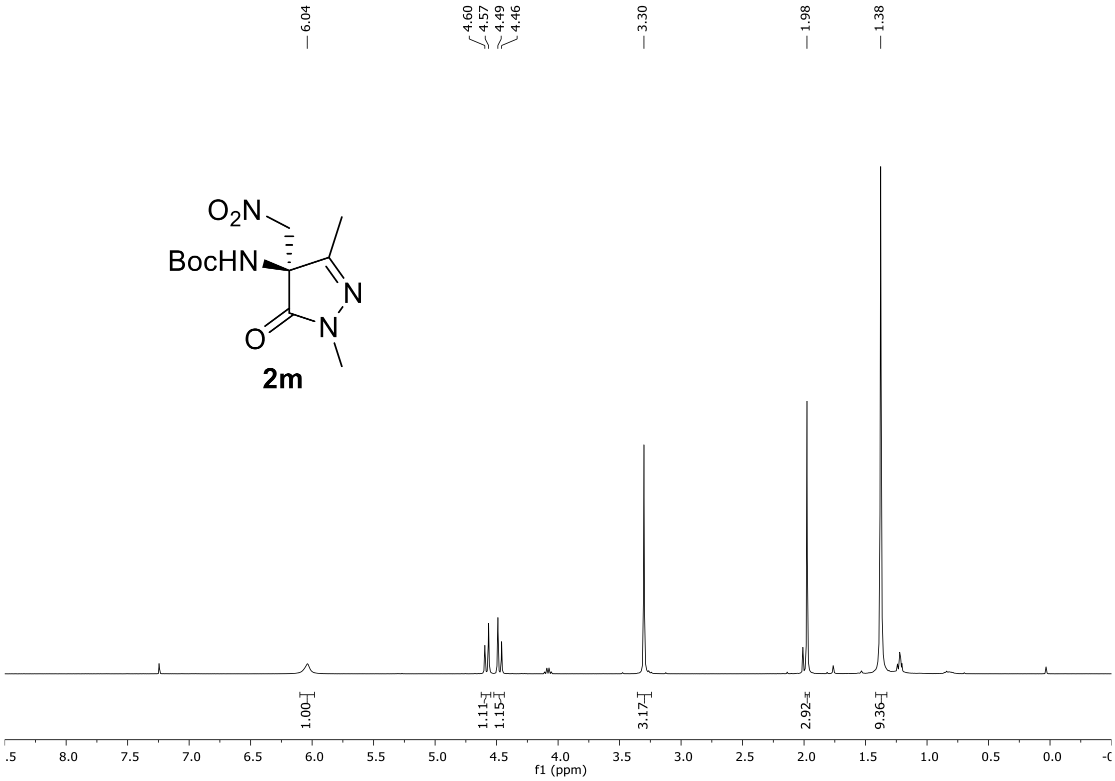

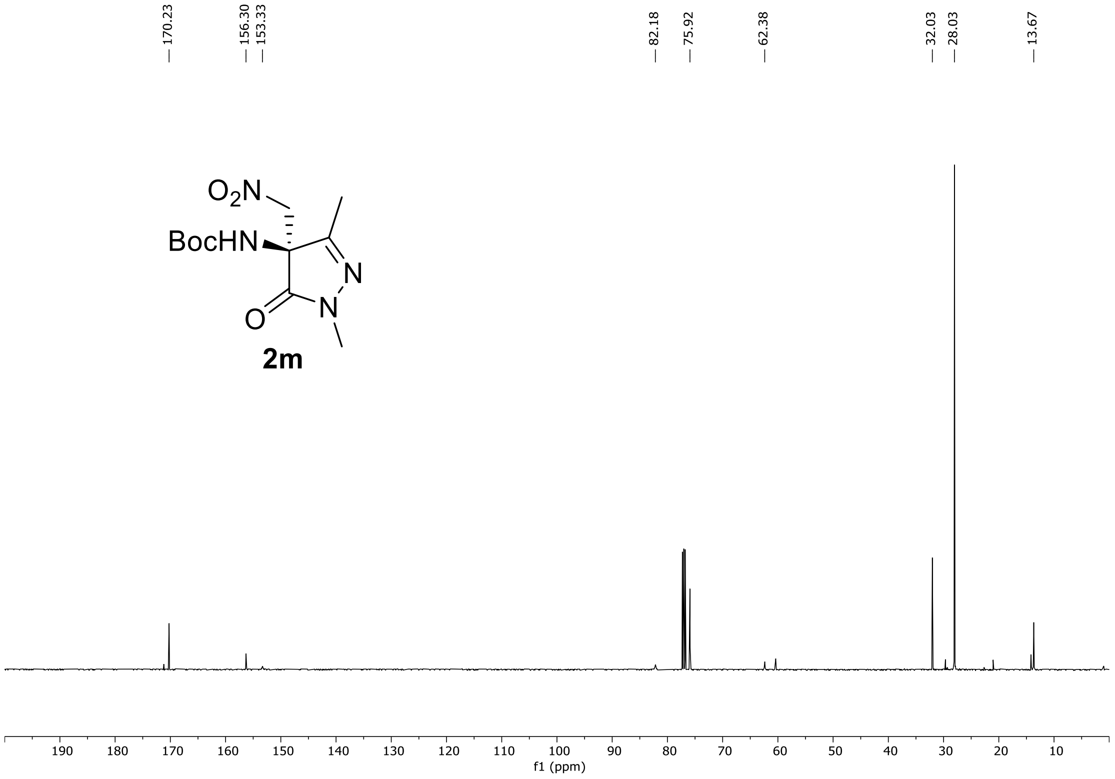

Figure S13. ^{1}H and ^{13}C NMR of **2m** ($CDCl_3$)

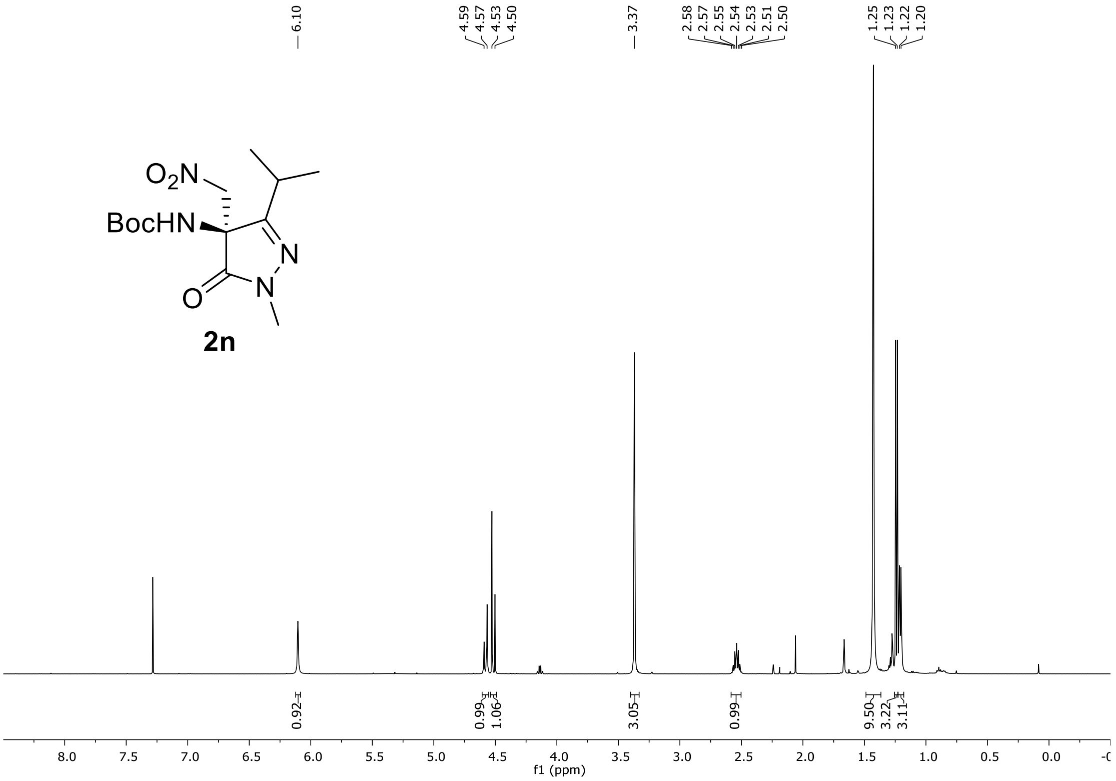

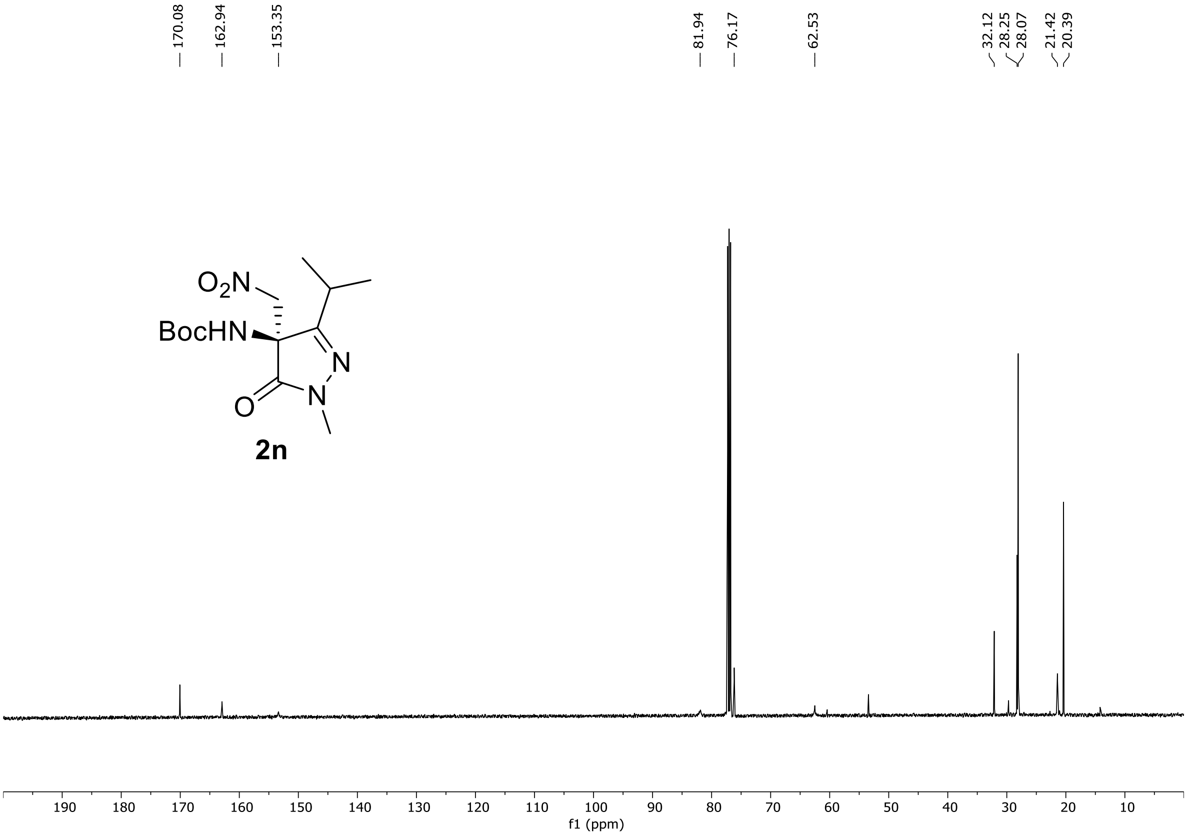

Figure S14. ^{1}H and ^{13}C NMR of **2n** ($CDCl_3$)

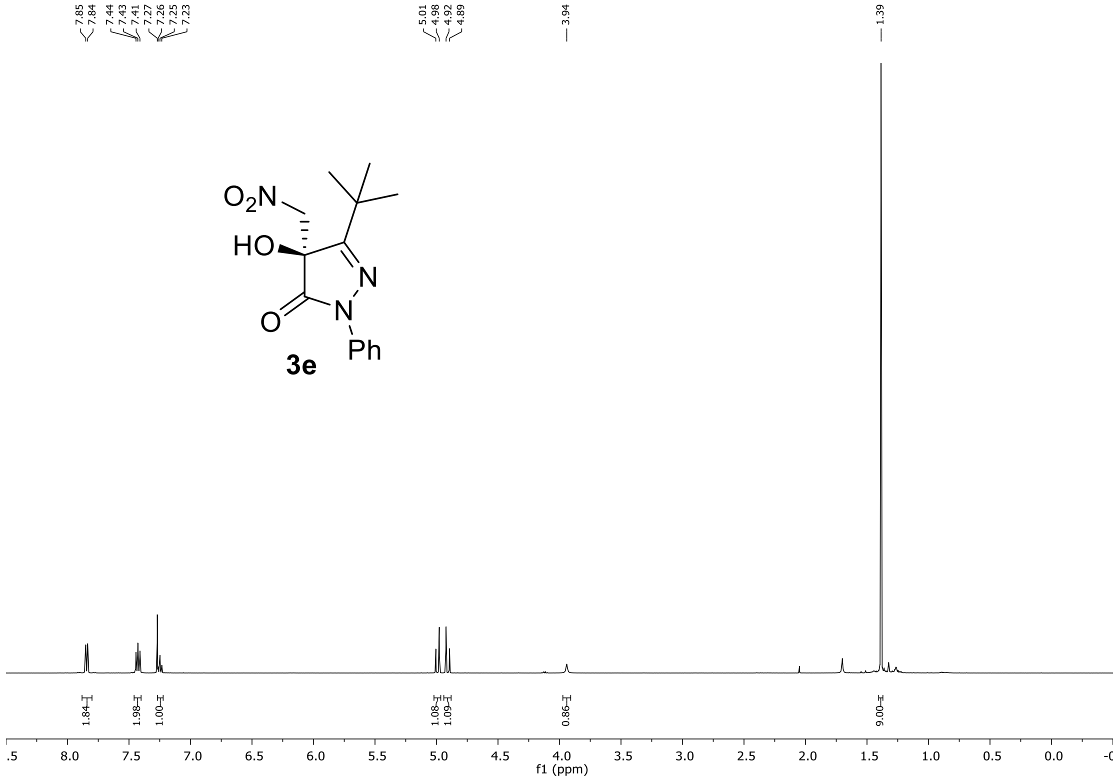

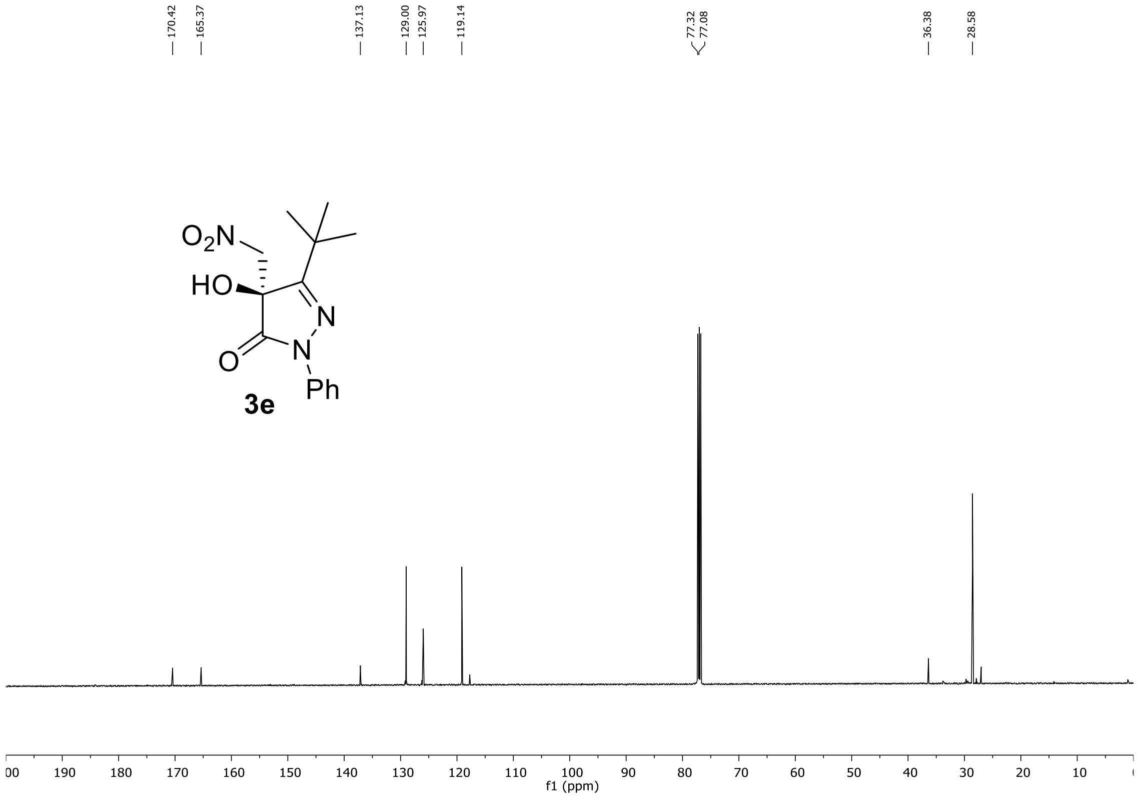

Figure S15. ^{1}H and ^{13}C NMR of **3a** ($CDCl_3$)

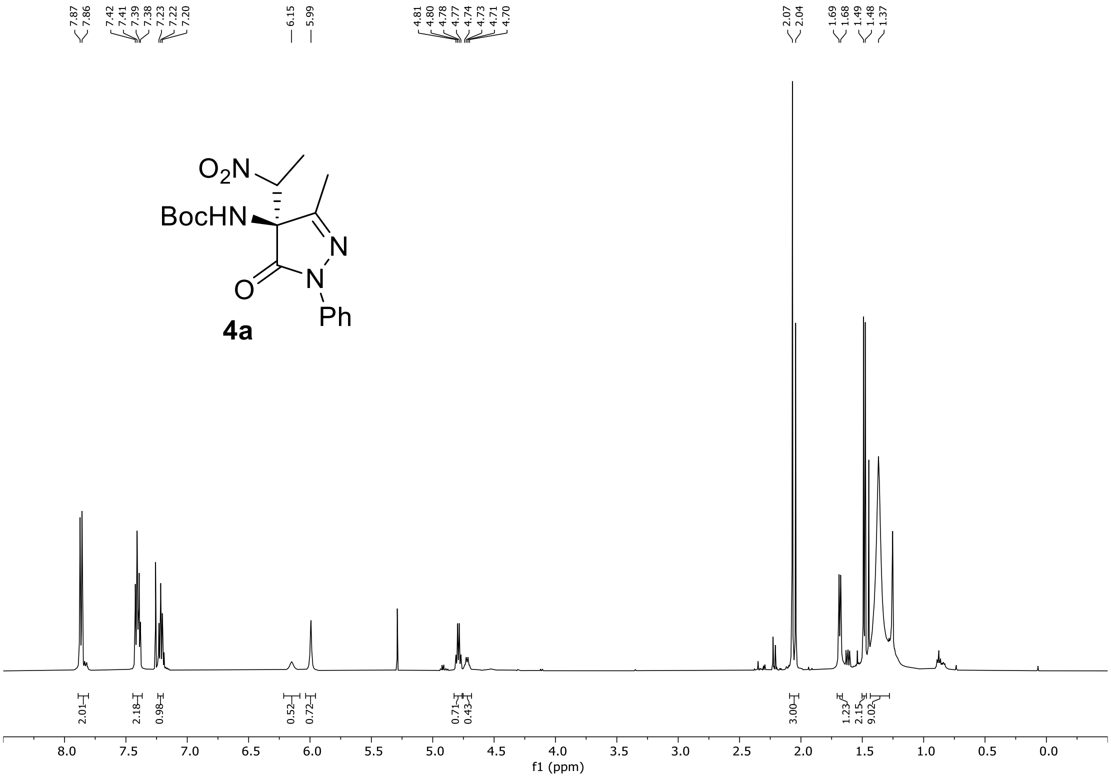

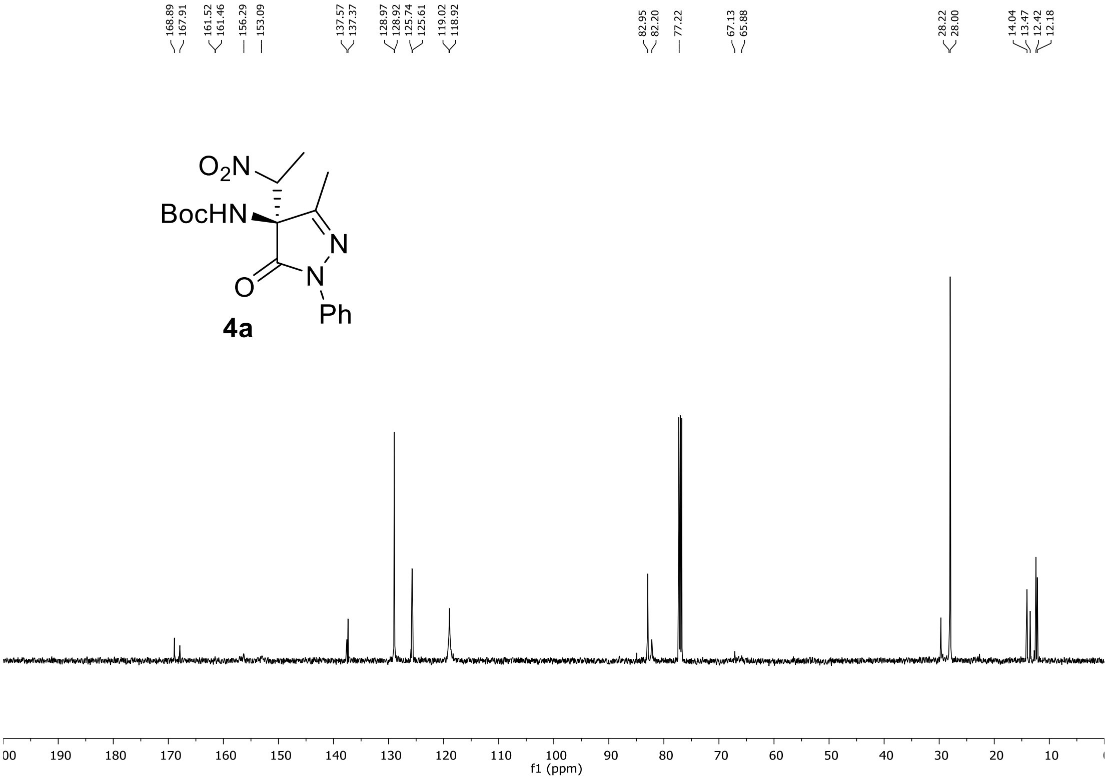

Figure S16. ^{1}H and ^{13}C NMR of **4a** ($CDCl_3$)

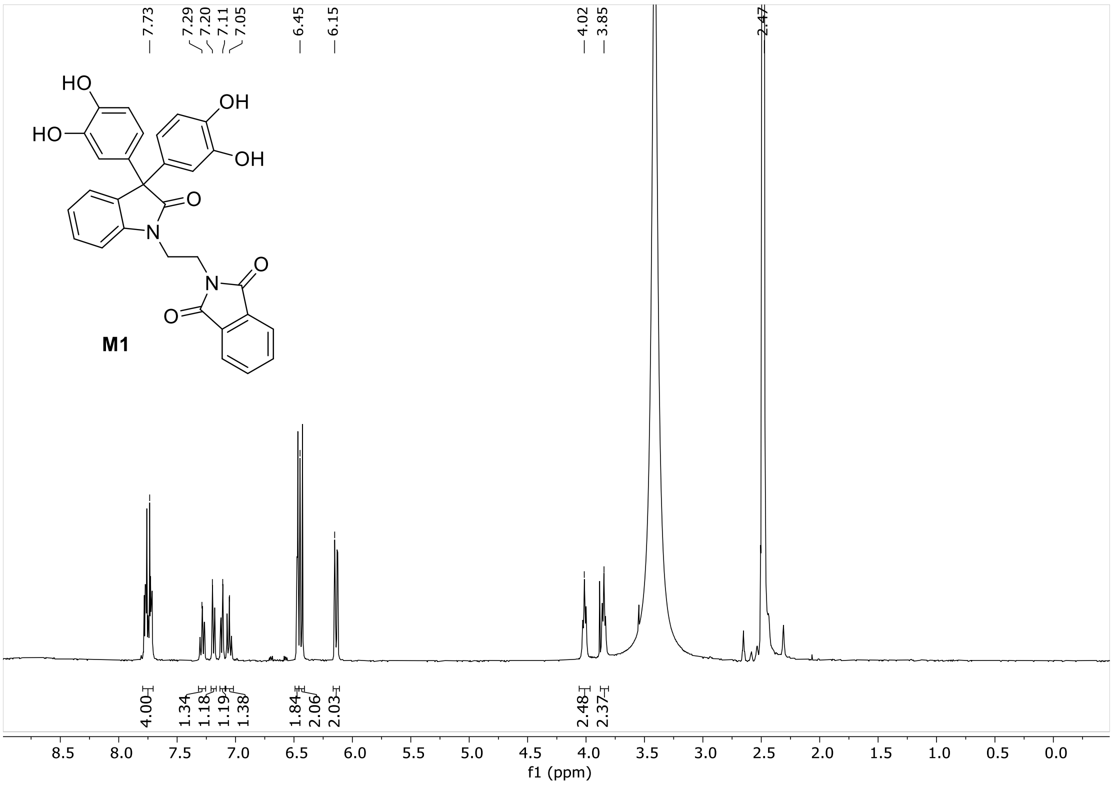

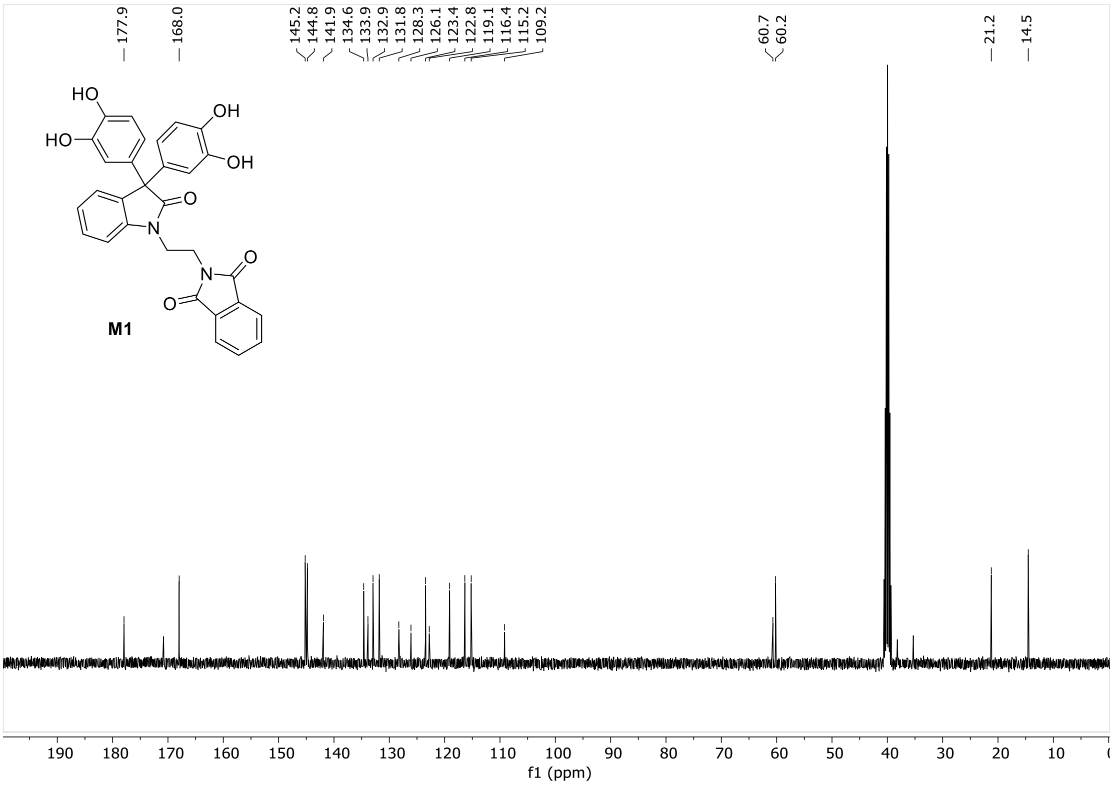

Figure S17. ^{1}H and ^{13}C NMR of **M1** (DMSO-d^6)

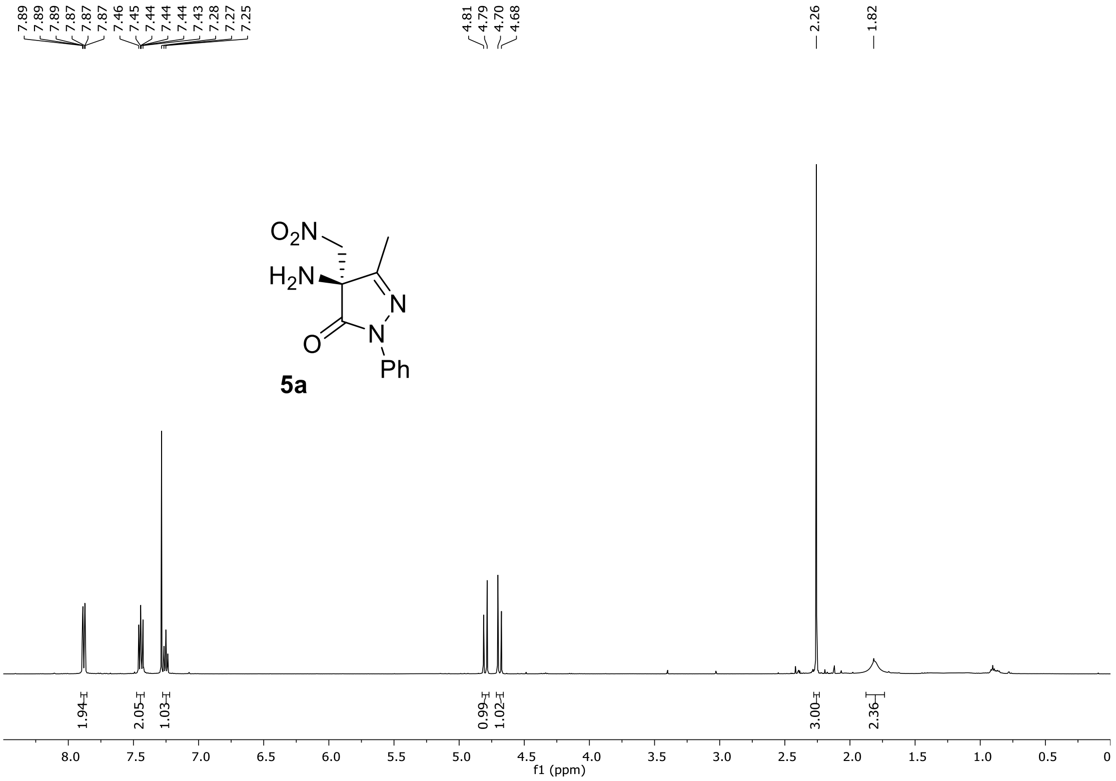

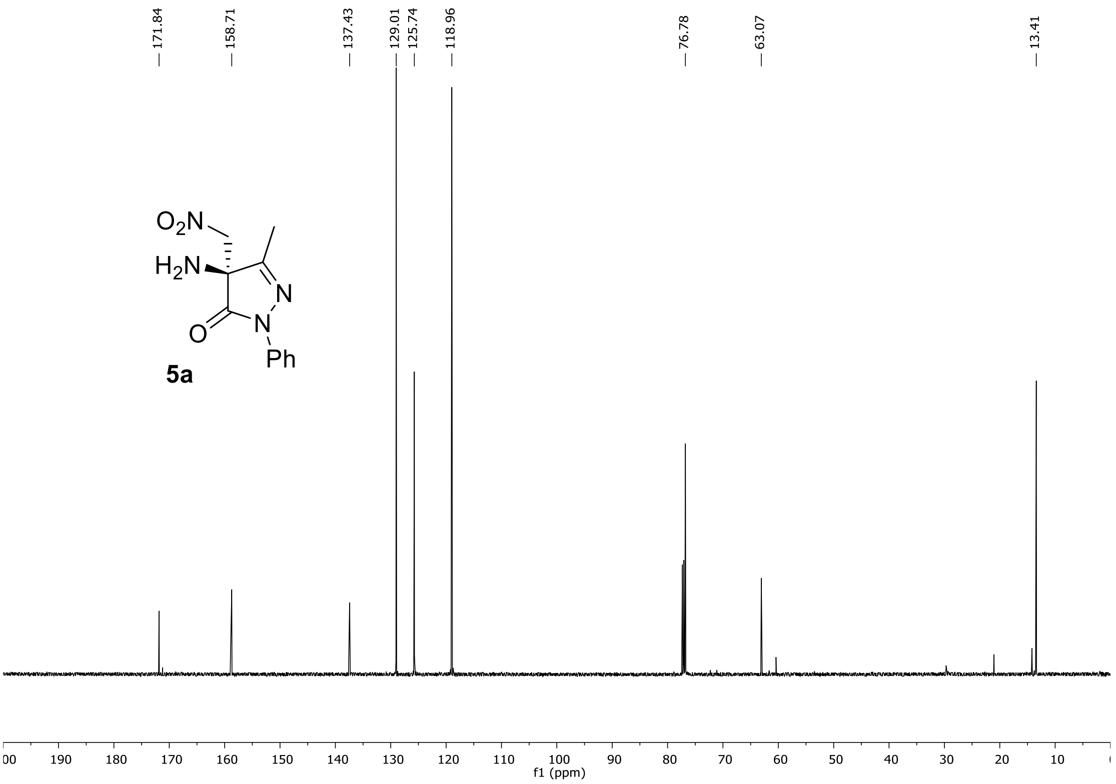

Figure S18. ^{1}H and ^{13}C NMR of **5a** ($CDCl_3$)

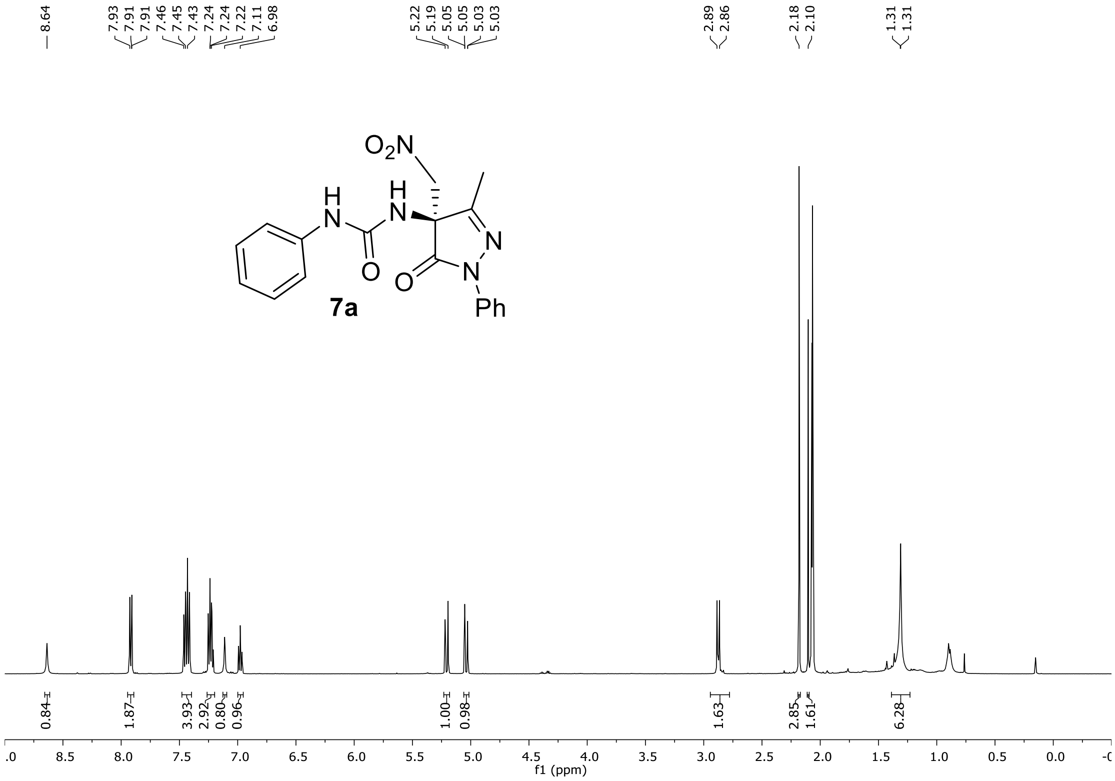

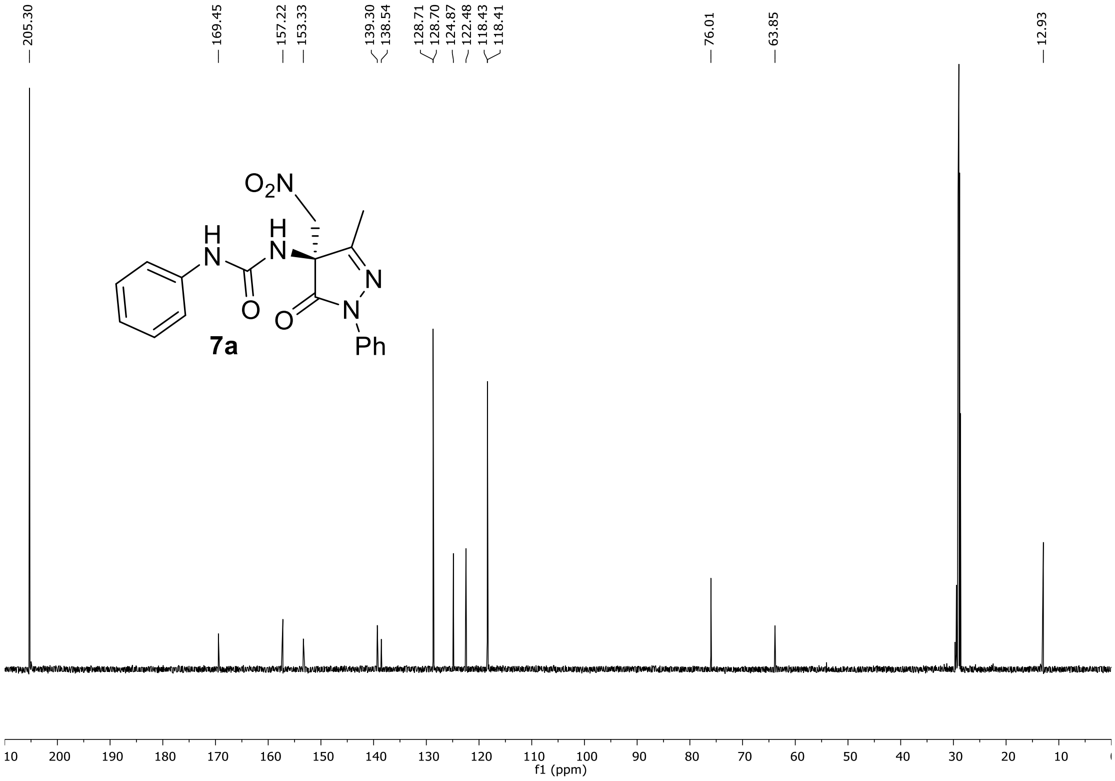

Figure S19. ^{1}H and ^{13}C NMR of **7a** (Acetone-d6)

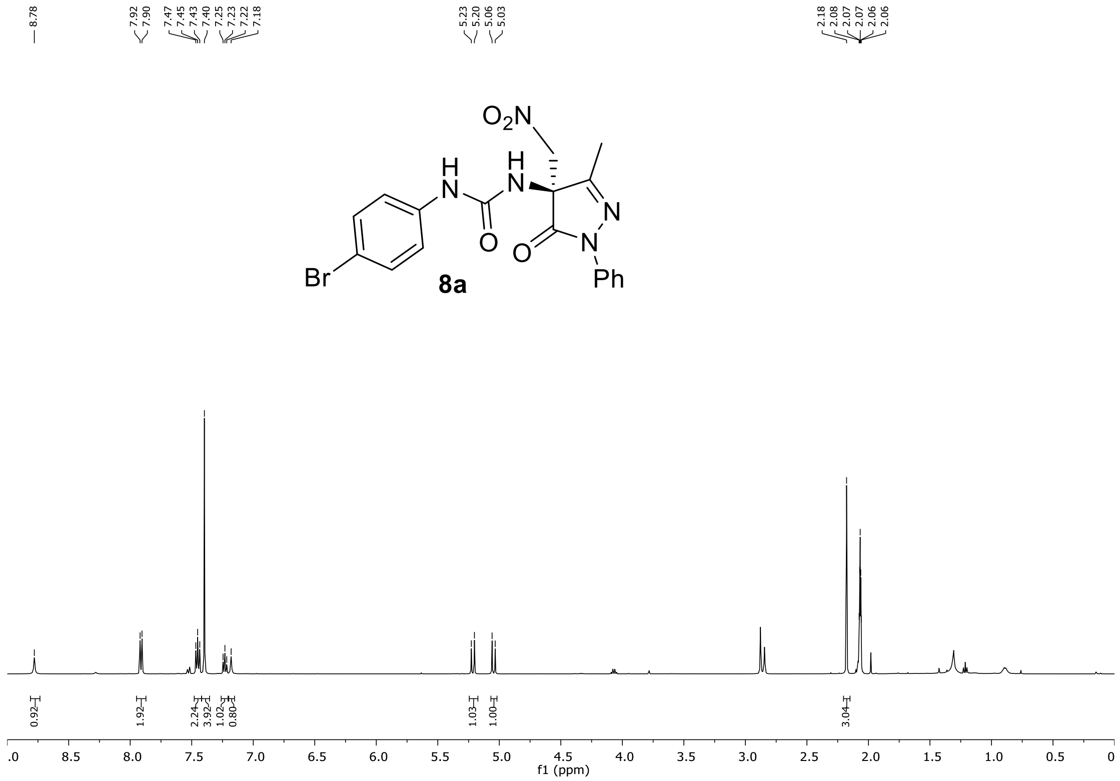

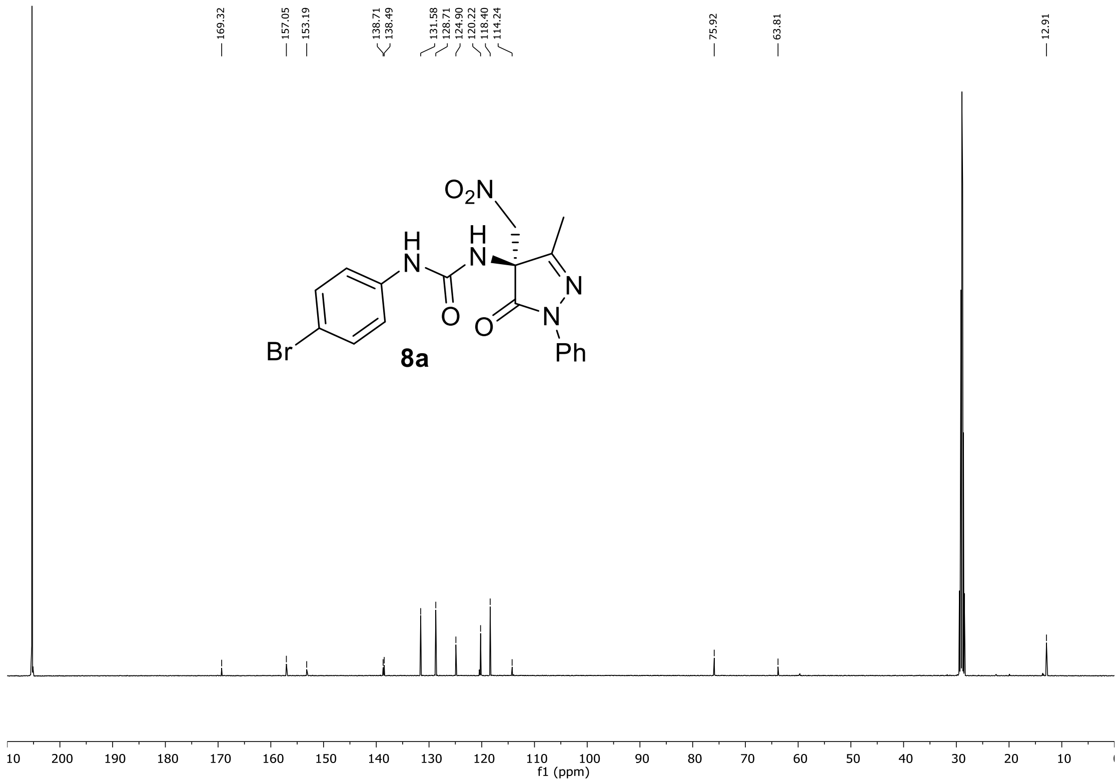

Figure S20. ^{1}H and ^{13}C NMR of **8a** (Acetone-d6)

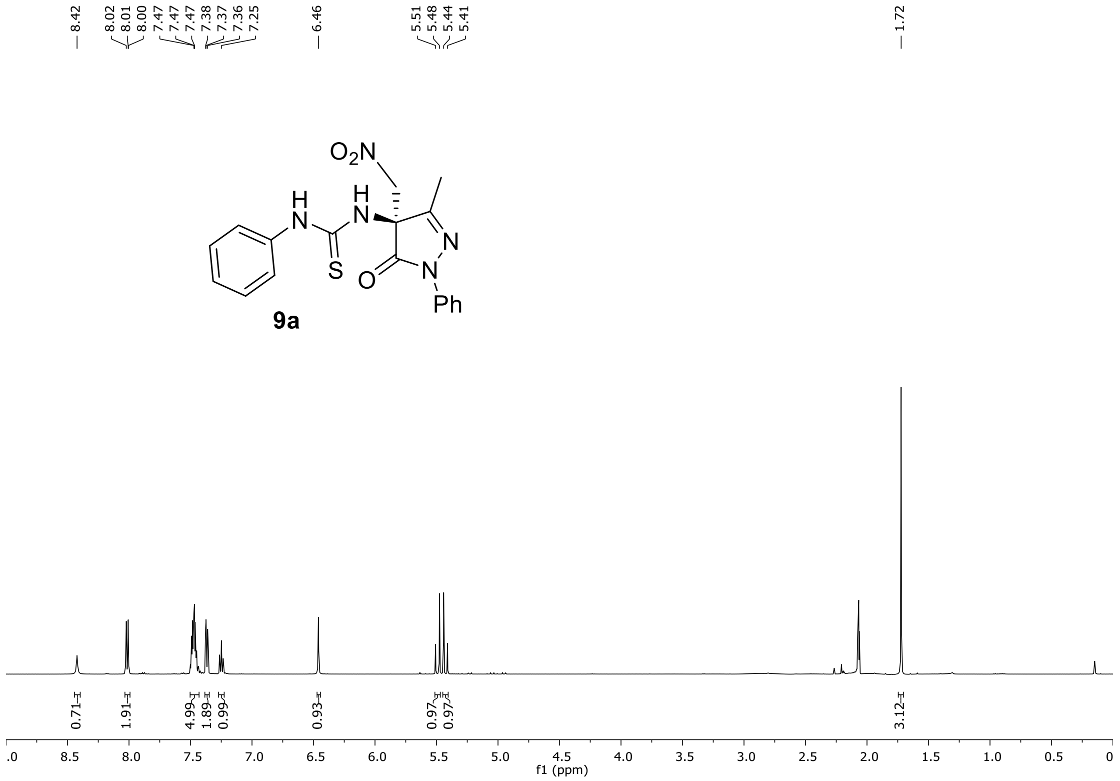

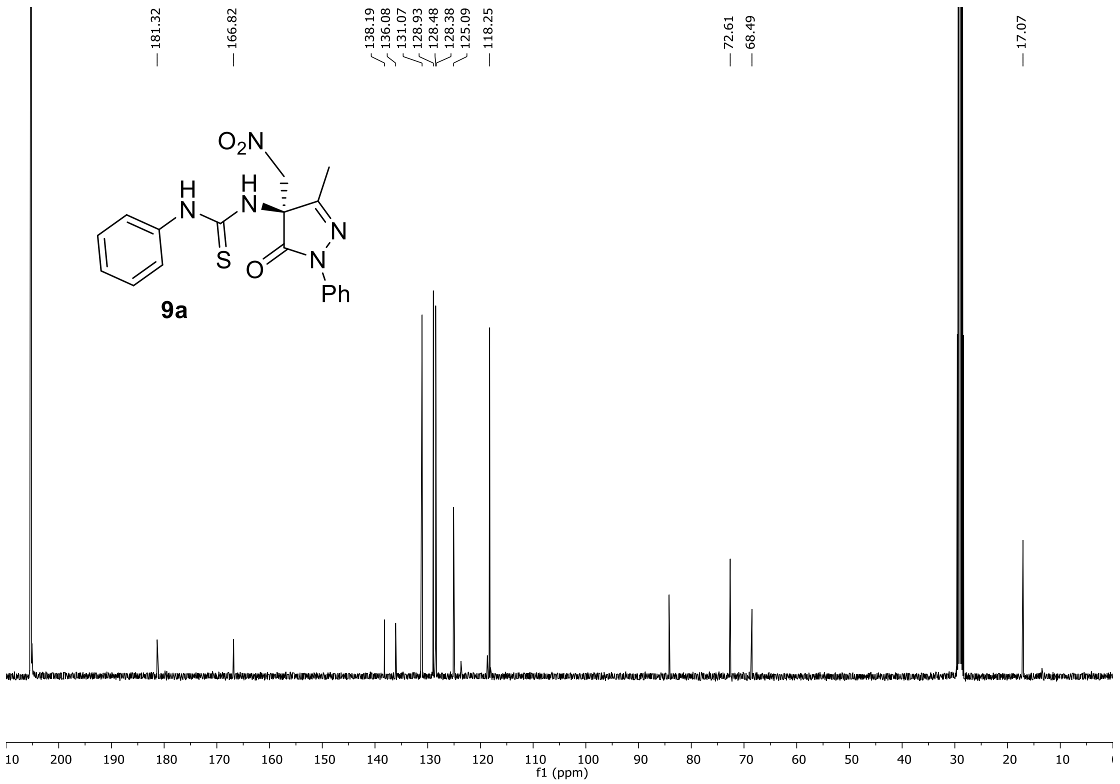

Figure S21. ^{1}H and ^{13}C NMR of **9a** (Acetone-d6)

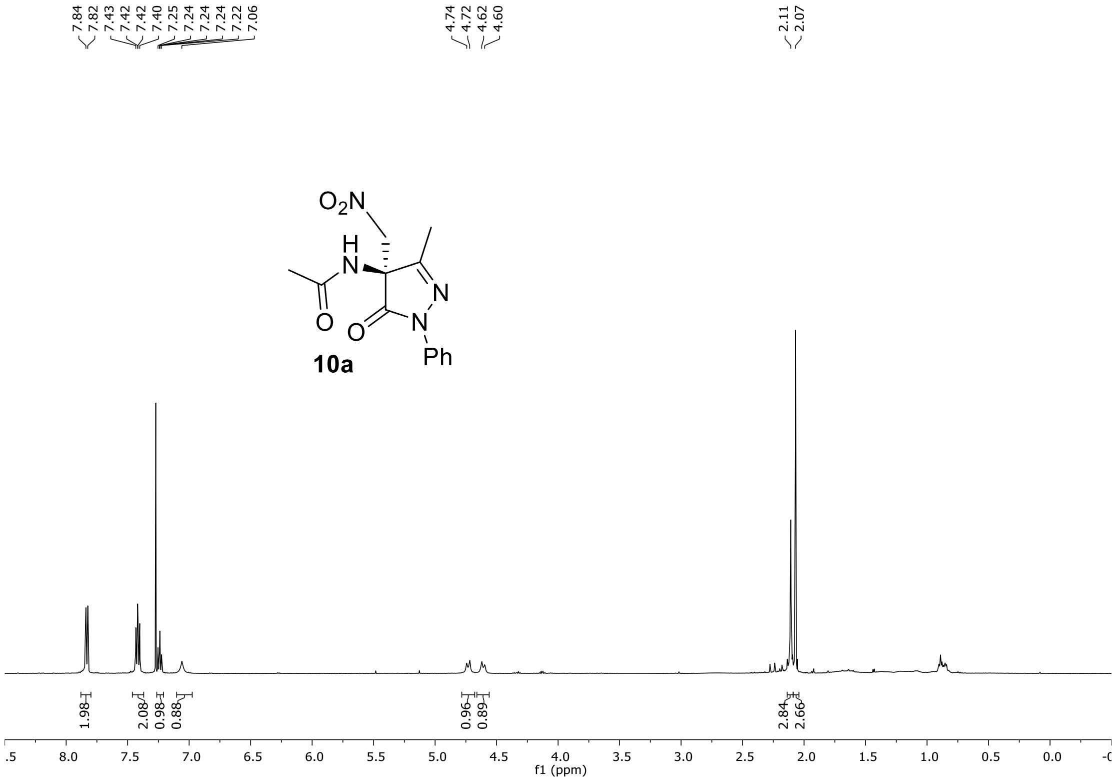

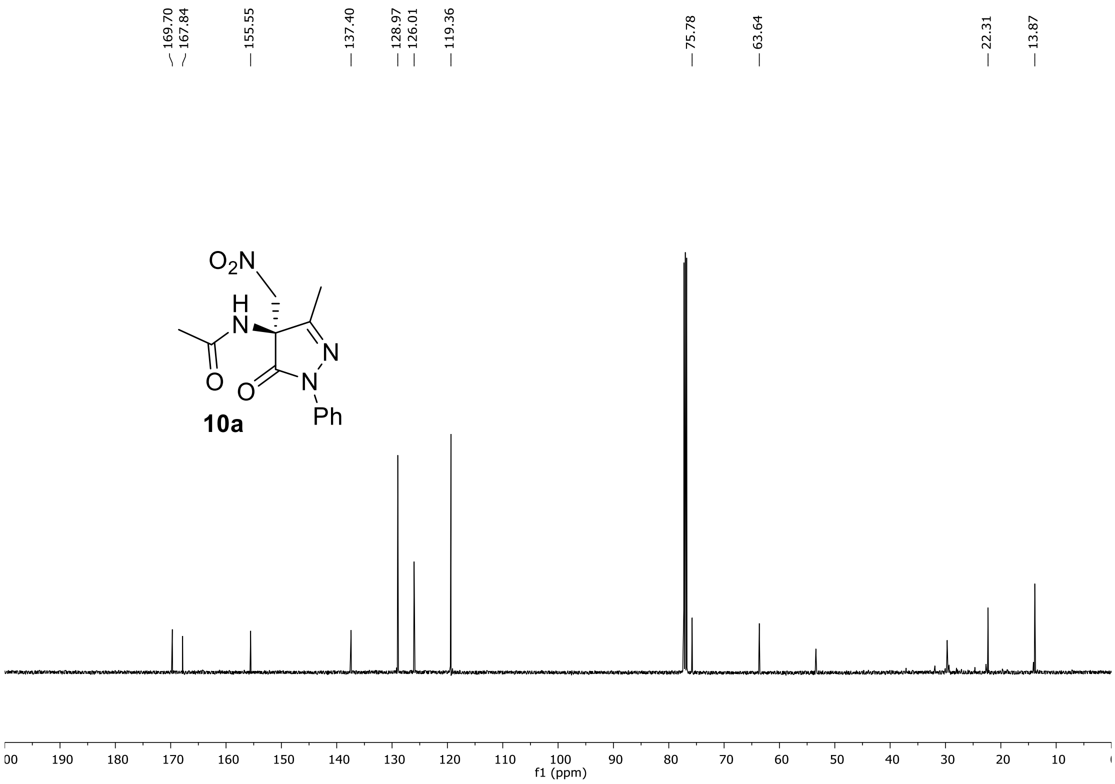

Figure S22. ^{1}H and ^{13}C NMR of **10a** ($CDCl_3$)

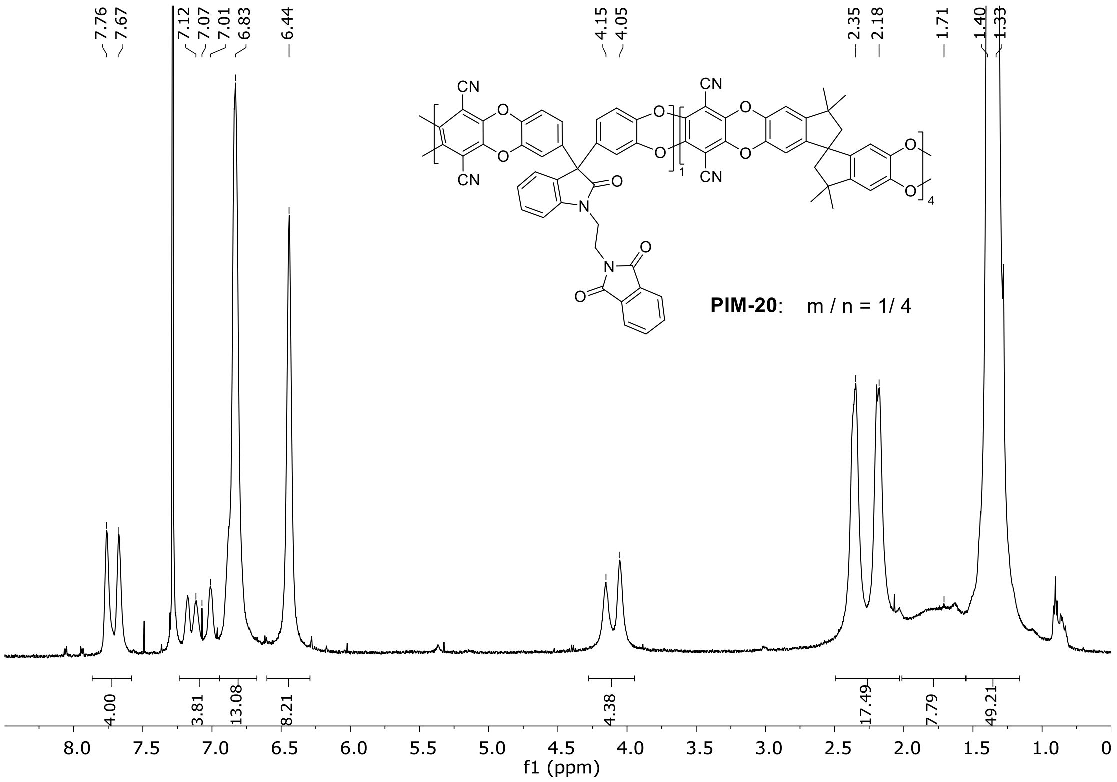

Figure S23. ^{1}H NMR of **PIM-20** ($CDCl_3$)

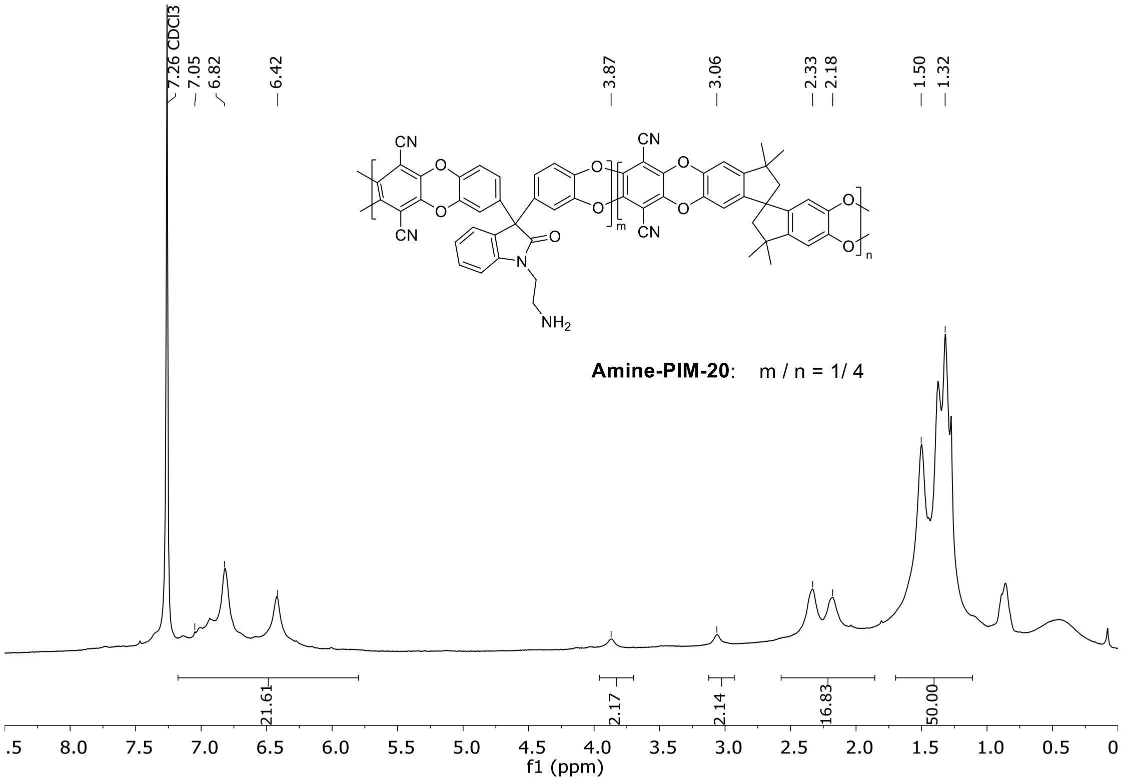

Figure S24. ^{1}H NMR of **PIM-20** ($CDCl_3$)

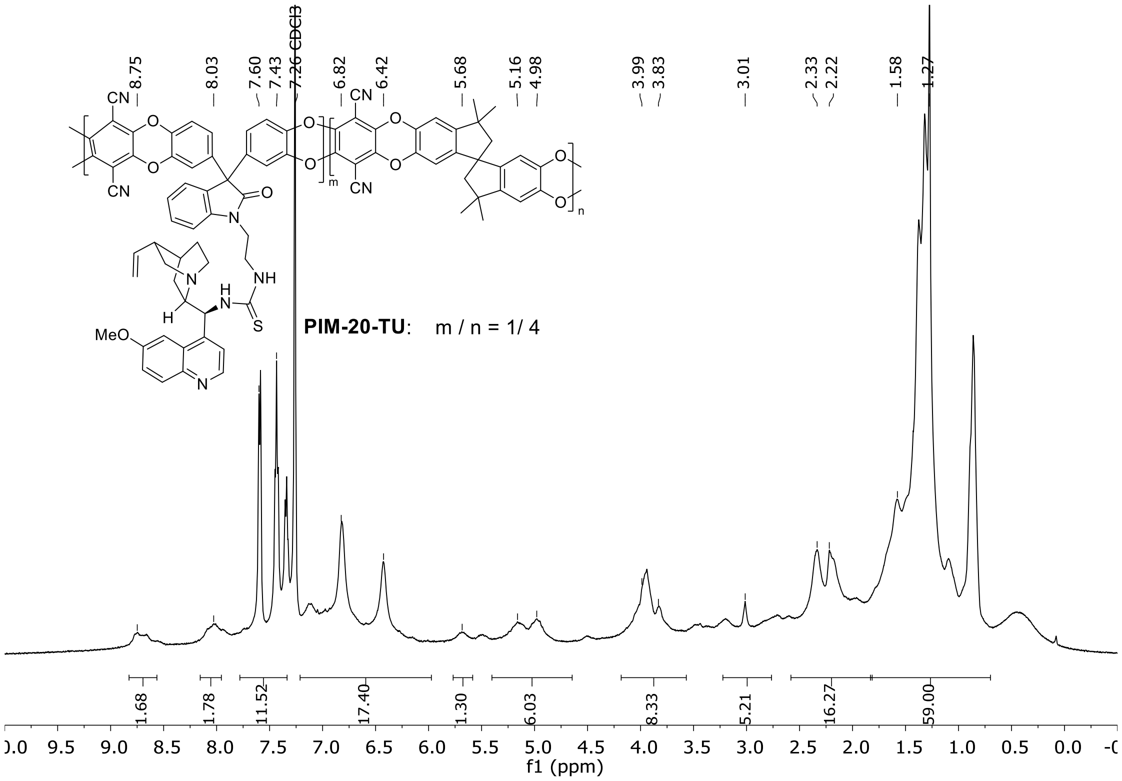

Figure S25. ^{1}H NMR of **PIM-20-TU** ($CDCl_3$)

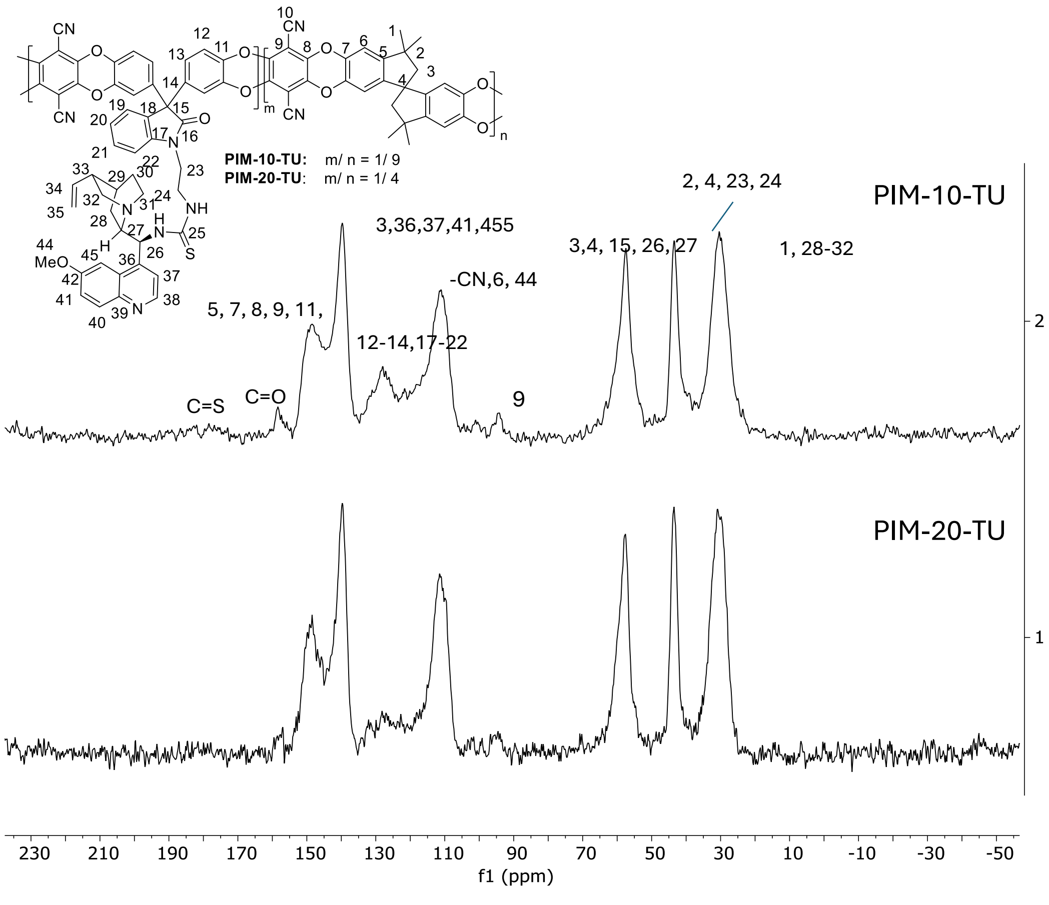

Figure S26. ^{13}C NMR CPMAS of **PIM-10-TU** and **PIM-20-TU**

5. FTIR Spectra of Polymers

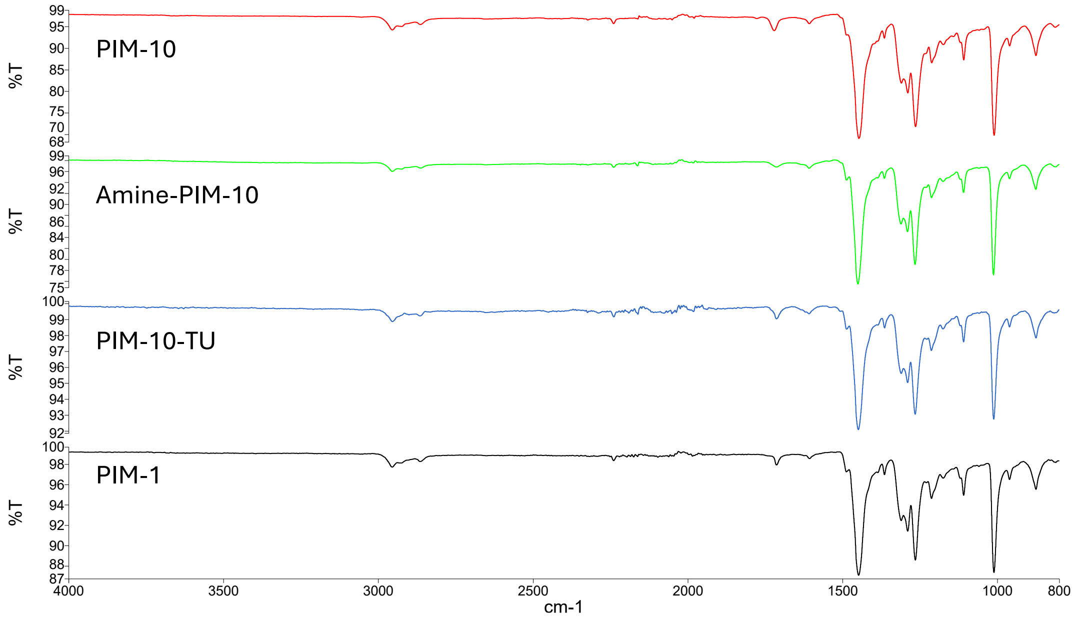

Figure S27. Comparison of FTIR spectra of PIM-10, amine-PIM-10, PIM-10-TU and PIM-1.

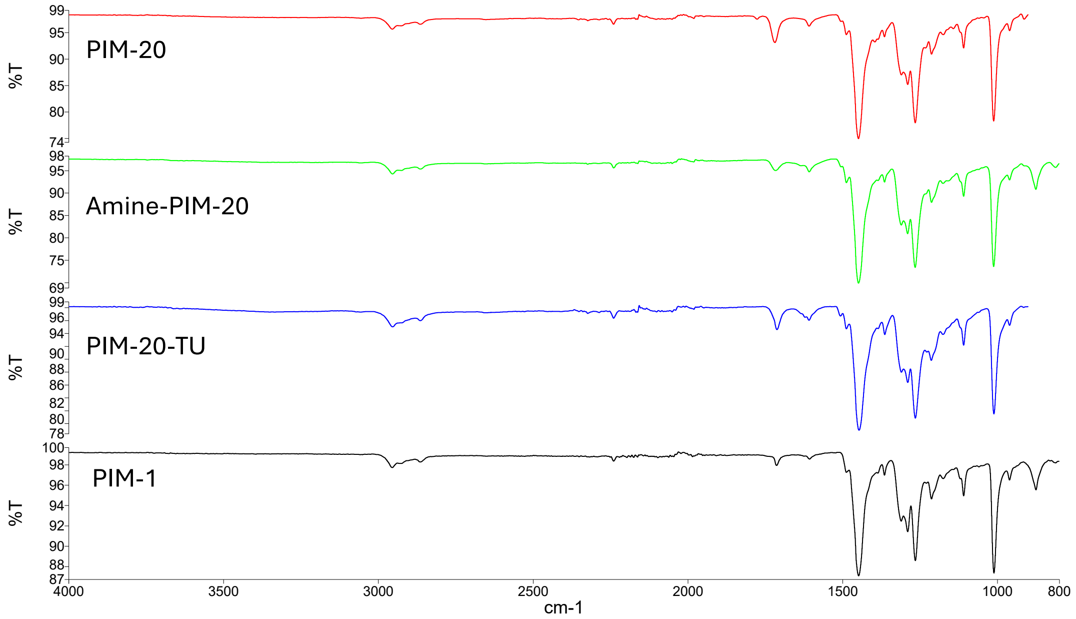

Figure S28. Comparison of FTIR spectra of PIM-20, amine-PIM-20, PIM-20-TU and PIM-1.

6. SEM Micrographs of the PIM-20 Family

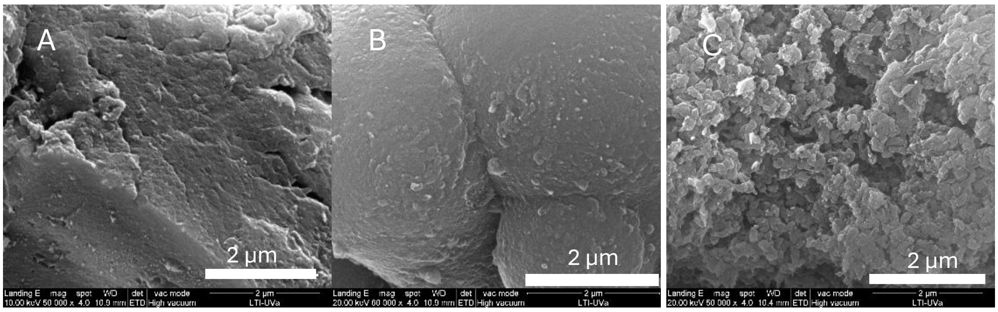

Figure S29.1. SEM images of the precursor and functionalized polymer samples: (A) PIM-20, (B) Amine-PIM-20, and (C) PIM-20-TU.

7. AFM Images

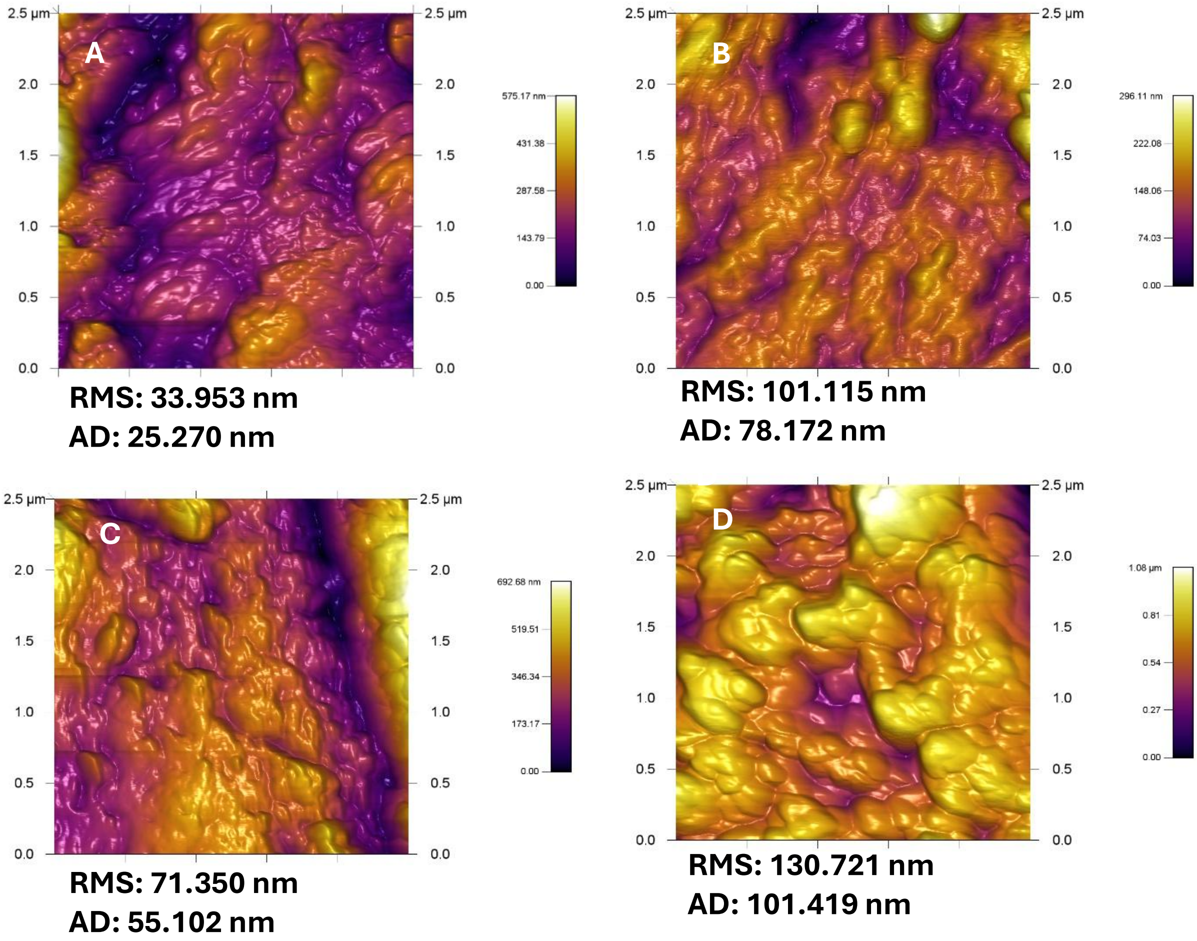

Figure S29.2. Tapping-mode AFM topographic images of the precursor and quinine-functionalized polymer powder: (A) PIM-10, (B) PIM-20, (C) PIM-10-TU and (D) PIM-20-TU. Roughness data (RMS =Root Mean Square, AD = Average deviation).

8. N_2 Sorption Isotherm of PIM-10 Powder at 77K.

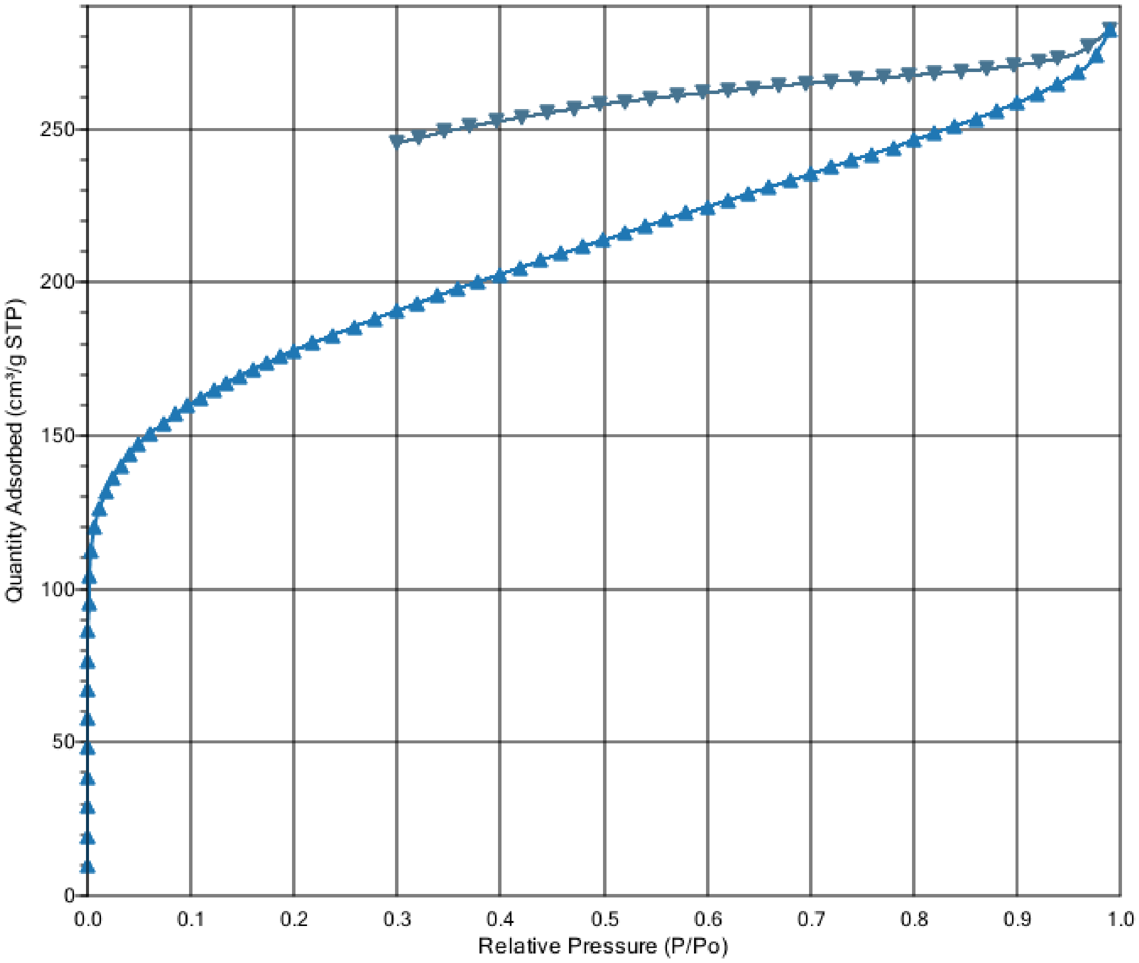

Figure S30. N_2 adsorption (dark blue symbols) − desorption (light blue symbols) isotherms measured at 77K for the PIM-10.

9. HPLC Profiles

tert-butyl (3-methyl-4-(nitromethyl)-5-oxo-1-phenyl-4,5-dihydro-1H-pyrazol-4-yl)carbamate (2a).

Racemic compound

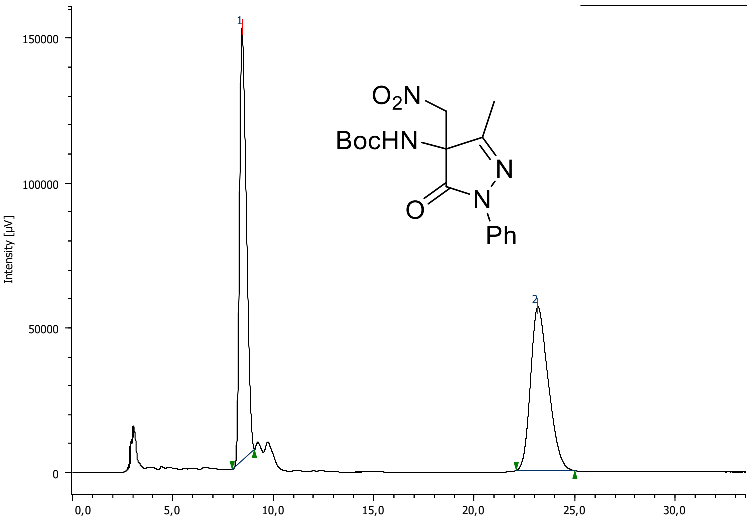

Peak Number	tR (min)	Area (u.a.)	Height (µV)	Area (%)	Symmetry Factor
1	**8,425**	3530801	149188	**50,207**	1,423
2	**23,133**	3501742	56452	**49,793**	1,298

HPLC profile for **2a** compound, er **89:11**

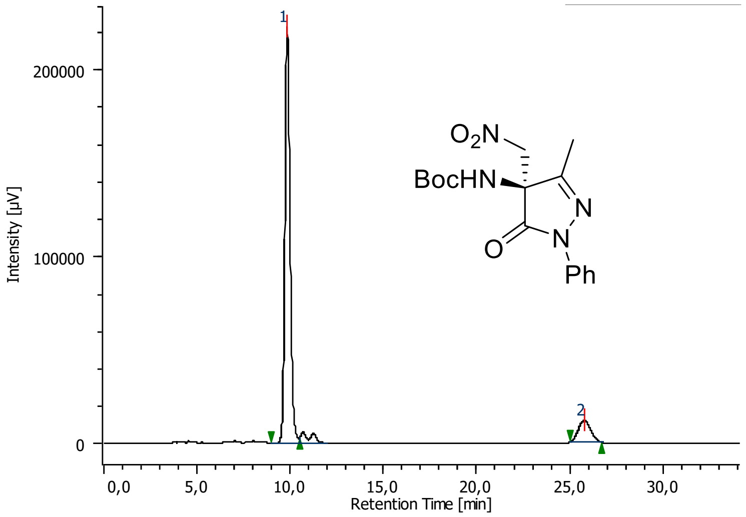

Peak Number	tR (min)	Area (u.a.)	Height (µV)	Area (%)	Symmetry Factor
1	**9,817**	4272067	222208	**88,935**	1,208
2	**25,725**	531523	11532	**11,065**	1,079

Figure S31. HPLC chromatograms of **2a**

tert-Butyl (S)-(3-ethyl-4-(nitromethyl)-5-oxo-1-phenyl-4,5-dihydro-1H-pyrazol-4-yl)carbamate (2b).

Racemic compound

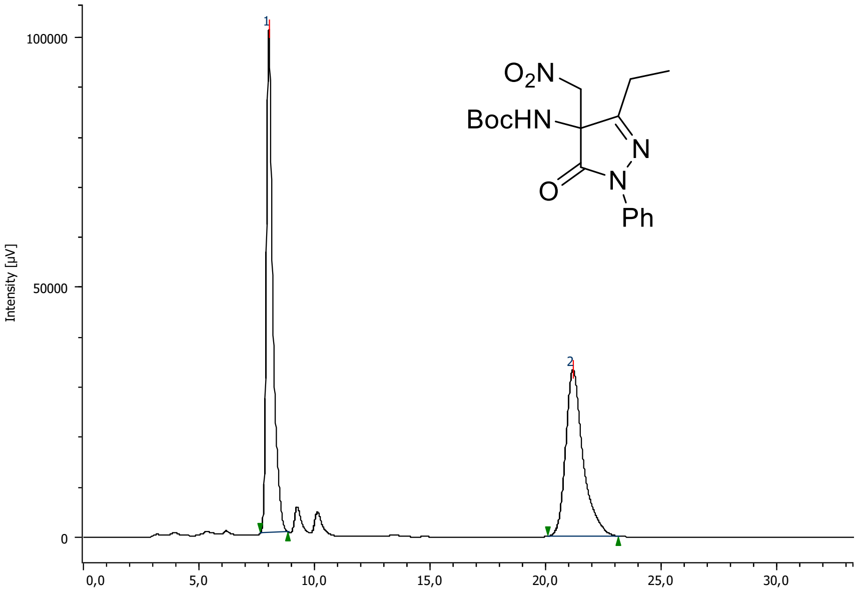

Peak Number	**t_R (min)**	Area (u.a.)	Height (µV)	**Area (%)**	Symmetry Factor
1	**8,017**	1941896	100504	**52,754**	1,582
2	**21,142**	1739130	33306	**47,246**	1,430

HPLC profile for **2b** compound, er **88:12**

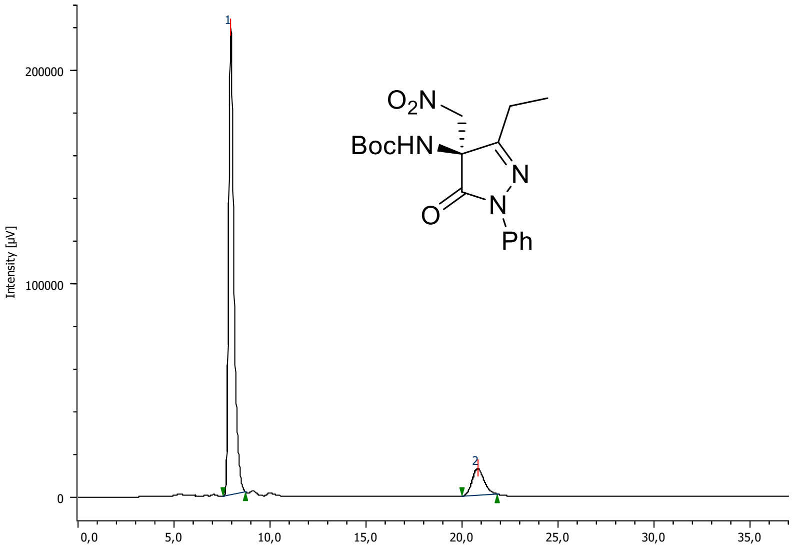

Peak Number	**t_R (min)**	Area (u.a.)	Height (µV)	**Area (%)**	Symmetry Factor
1	**7,950**	4107759	218033	**88,396**	1,510
2	**20,792**	539230	12420	**11,604**	1,174

Figure S32. HPLC chromatograms of **2b**

tert-Butyl (S)-(3-isopropyl-4-(nitromethyl)-5-oxo-1-phenyl-4,5-dihydro-1H-pyrazol-4-yl)carbamate (2c).

Racemic compound

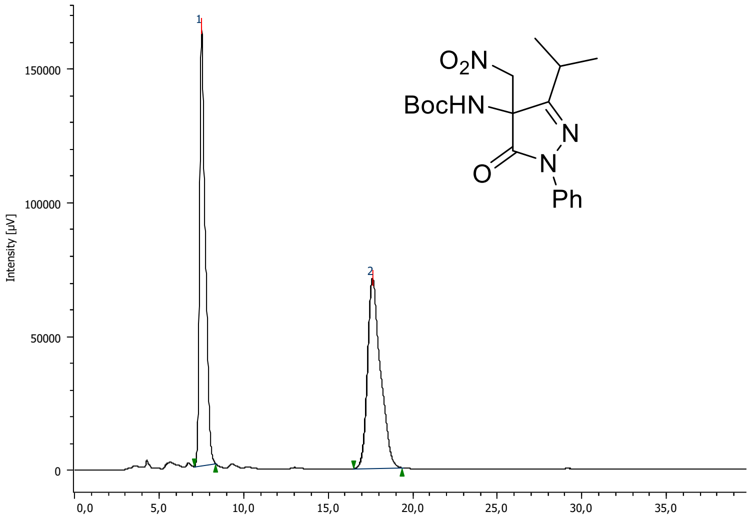

Peak Number	**t_R (min)**	Area (u.a.)	Height (µV)	**Area (%)**	Symmetry Factor
1	**7,508**	3760953	163959	**50,003**	1,525
2	**17,600**	3760495	70983	**49,997**	1,358

HPLC profile for **2c** compound, er **85:15**

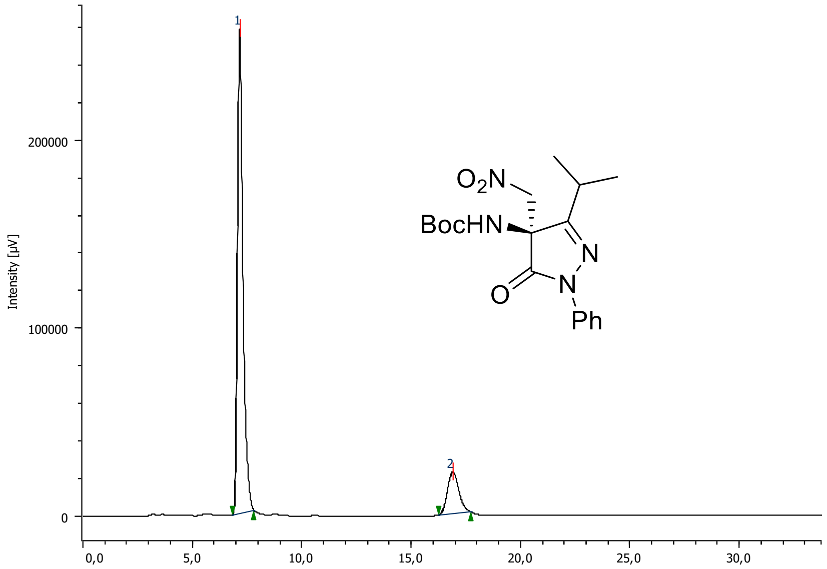

Peak Number	**t_R (min)**	Area (u.a.)	Height (µV)	**Area (%)**	Symmetry Factor
1	**7,158**	4580016	257107	**85,254**	1,478
2	**16,883**	792155	22034	**14,746**	1,164

Figure S33. HPLC chromatograms of **2c**

tert-Butyl (S)-(3-cyclopropyl-4-(nitromethyl)-5-oxo-1-phenyl-4,5-dihydro-1H-pyrazol-4-yl) carbamate (2d).

Racemic compound

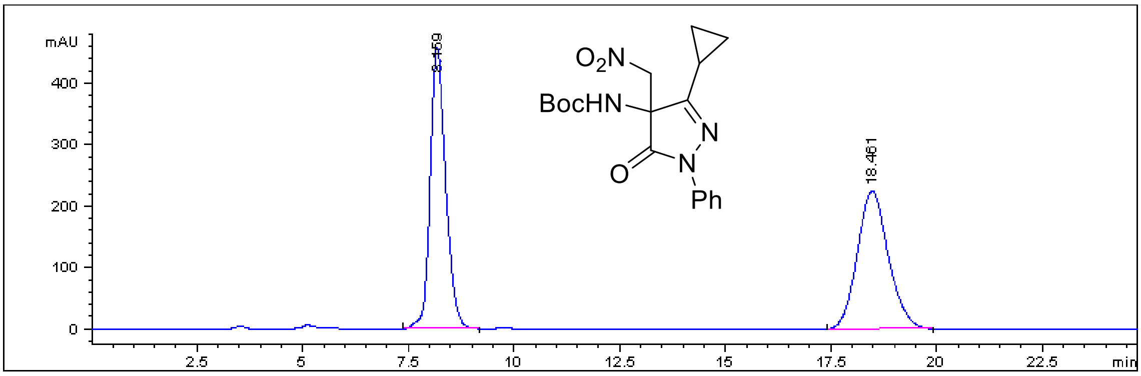

Peak Number	**t_R (min)**	Area (u.a.)	Height (µV)	**Area (%)**	Symmetry Factor
1	**8.159**	11806.8	458.9	**50.514**	0.736
2	**18.461**	11566.5	224.7	**49.486**	0.876

HPLC profile for **2d** compound, er **88:12**

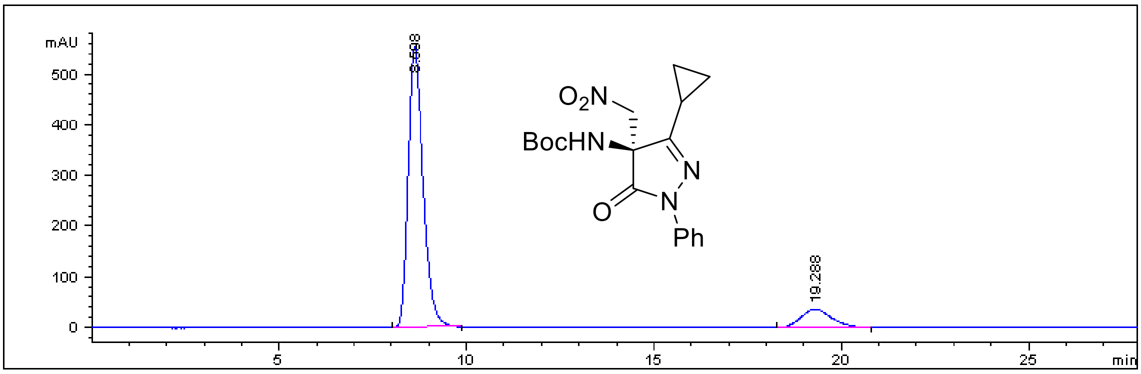

Peak Number	**t_R (min)**	Area (u.a.)	Height (µV)	**Area (%)**	Symmetry Factor
1	**8.598**	15440.5	553.4	**87.854**	0.692
2	**19.288**	2134.7	37.1	**12.146**	0.818

Figure S34. HPLC chromatograms of **2d**

tert-Butyl (S)-(4-(nitromethyl)-5-oxo-1,3-diphenyl-4,5-dihydro-1H-pyrazol-4-yl)carbamate (2f).

Racemic compound

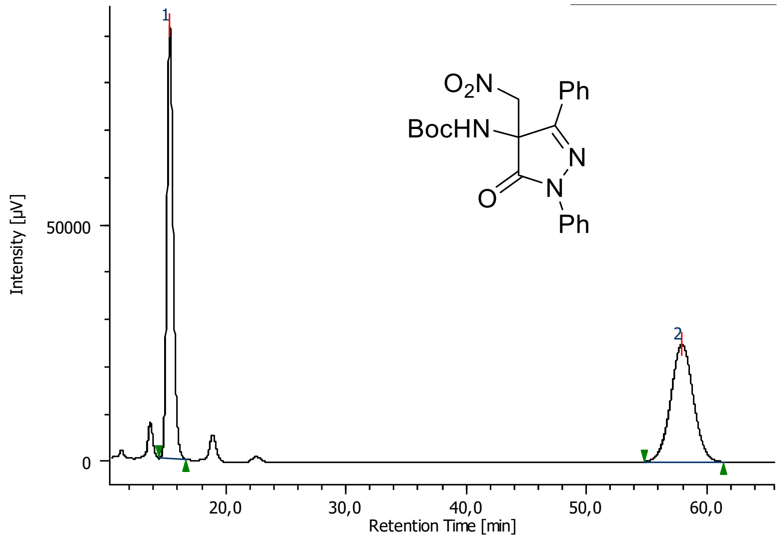

Peak Number	**t_R (min)**	Area (u.a.)	Height (µV)	**Area (%)**	Symmetry Factor
1	**15,342**	3284851	91360	**50,309**	1,175
2	**57,858**	3244454	24817	**49,691**	1,016

HPLC profile for **2f** compound, er **73:27**

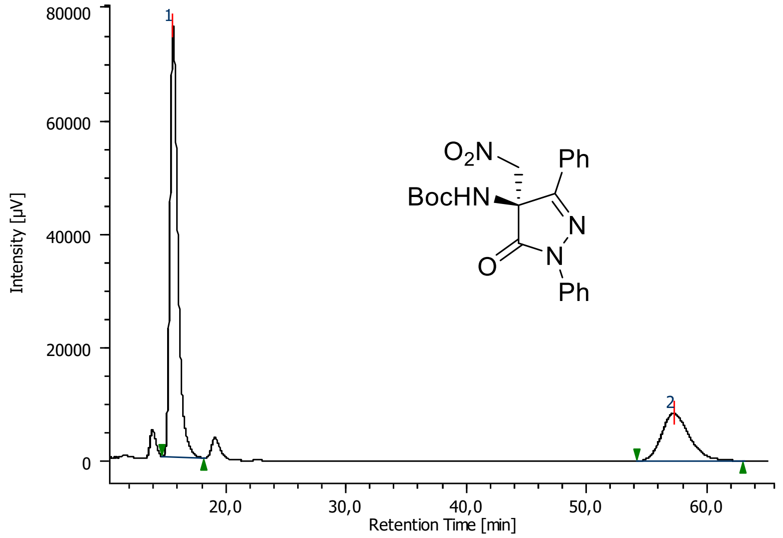

Peak Number	**t_R (min)**	Area (u.a.)	Height (µV)	**Area (%)**	Symmetry Factor
1	**15,617**	3437326	75688	**72,880**	1,487
2	**57,167**	1279062	8219	**27,120**	1,184

Figure S35. HPLC chromatograms of **2f**

tert-butyl (S)-(3-methyl-4-(nitromethyl)-5-oxo-1-(p-tolyl)-4,5-dihydro-1H-pyrazol-4-yl)carbamate (2g).

Racemic compound

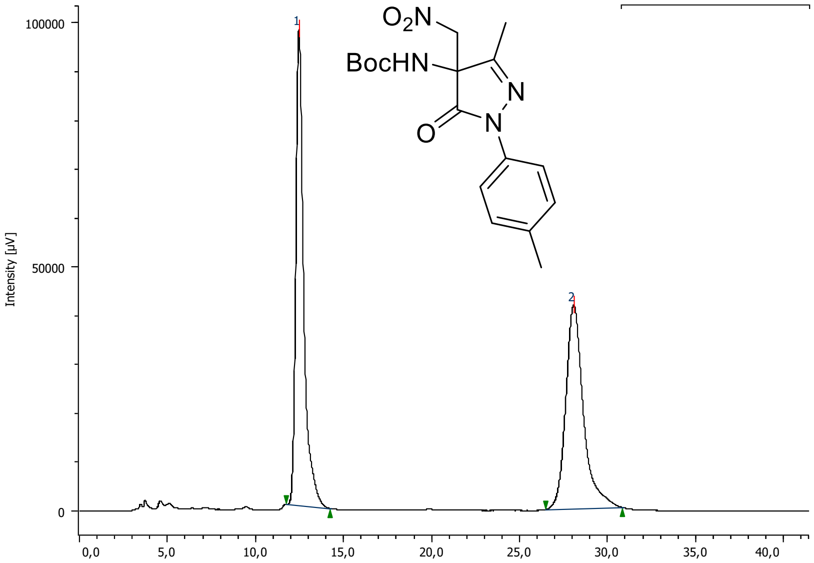

Peak Number	t_R (min)	Area (u.a.)	Height (µV)	Area (%)	Symmetry Factor
1	**12,458**	3029941	97467	**50,958**	1,632
2	**28,042**	2916059	41766	**49,042**	1,381

HPLC profile for **2g** compound, er **89:11**

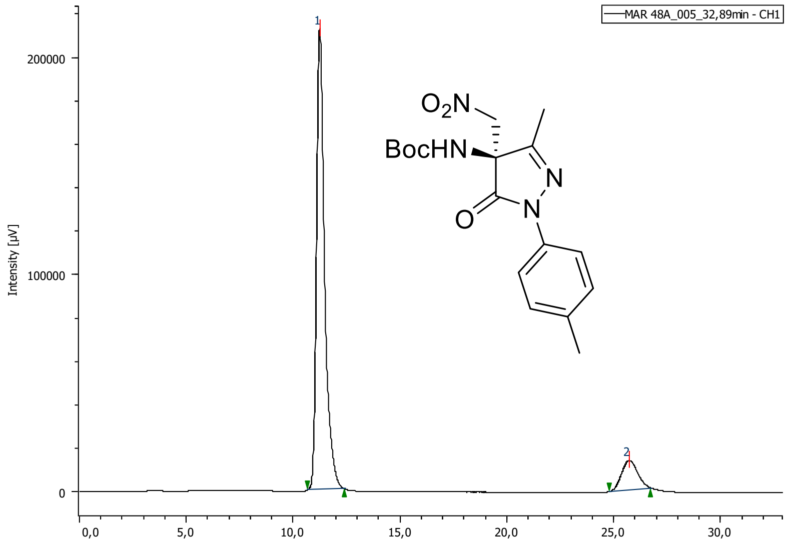

Peak Number	t_R (min)	Area (u.a.)	Height (µV)	Area (%)	Symmetry Factor
1	**11,233**	5619640	211885	**88,926**	1,538
2	**25,700**	699829	13512	**11,074**	1,086

Figure S36. HPLC chromatograms of **2g**

tert-Butyl (S)-(1-(4-bromophenyl)-3-methyl-4-(nitromethyl)-5-oxo-4,5-dihydro-1H-pyrazol-4-yl) carbamate (2h).

Racemic compound

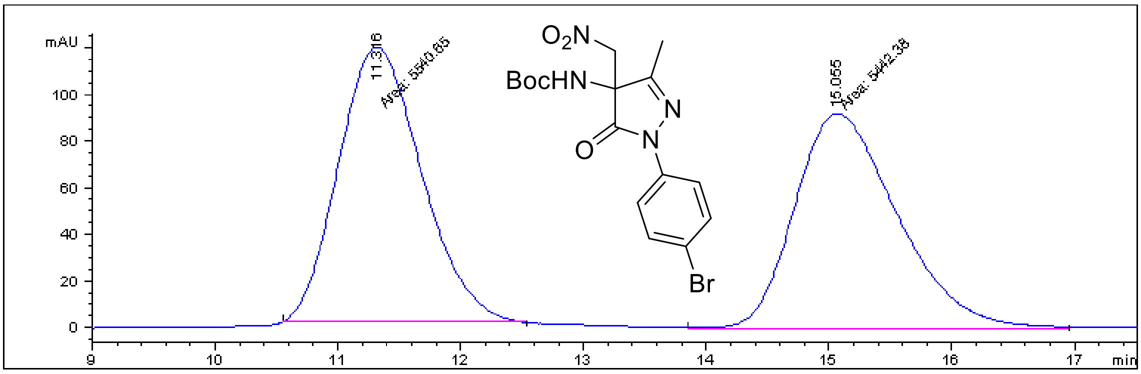

Peak Number	t_R (min)	Area (u.a.)	Height (µV)	Area (%)	Symmetry Factor
1	**11.316**	5540.6	117.9	**50.447**	0.844
2	**15.055**	5442.4	92.5	**49.553**	0.668

HPLC profile for **2h** compound, er **88:12**

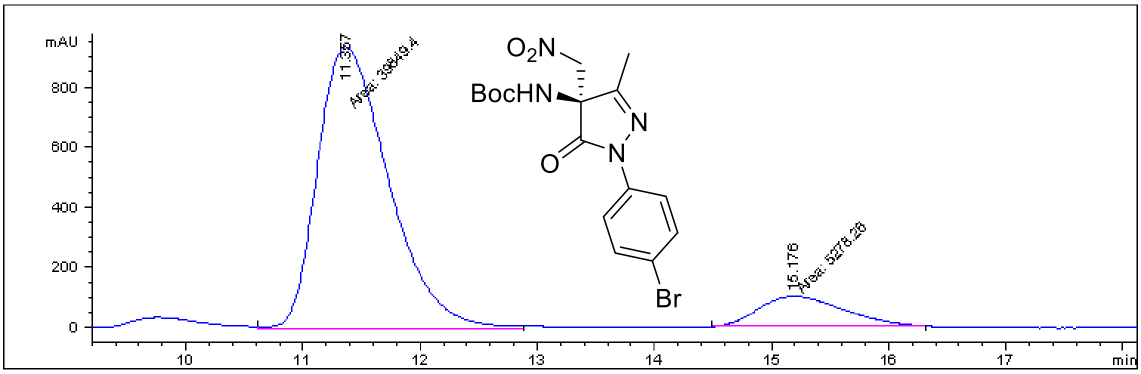

Peak Number	t_R (min)	Area (u.a.)	Height (µV)	Area (%)	Symmetry Factor
1	**11.357**	39849.4	938.8	**88.304**	0.625
2	**15.176**	5278.3	102.4	**11.696**	0.652

Figure S37. HPLC chromatograms of **2h**

tert-Butyl (S)-(1-(4-chlorophenyl)-3-methyl-4-(nitromethyl)-5-oxo-4,5-dihydro-1H-pyrazol-4-yl) carbamate (2i).

Racemic compound

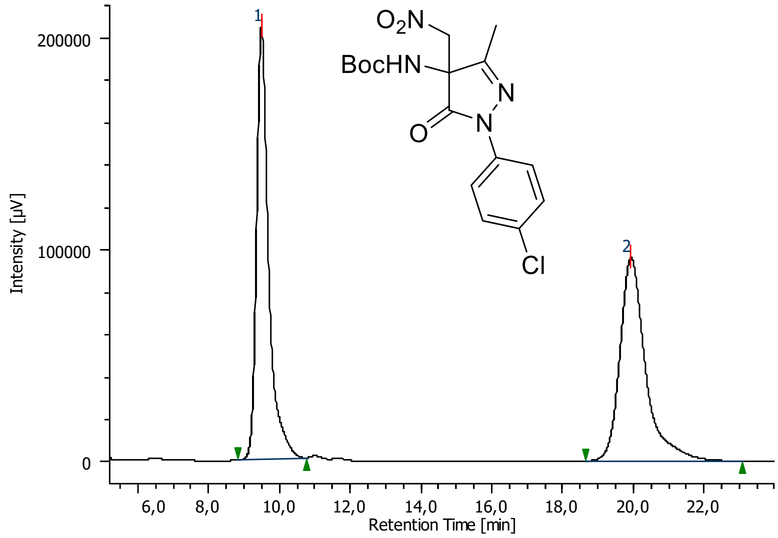

Peak Number	**t_R (min)**	Area (u.a.)	Height (µV)	**Area (%)**	Symmetry Factor
1	**9.492**	4780237	203807	**50.320**	1.587
2	**19.908**	4719381	95437	**49.680**	1.441

HPLC profile for **2i** compound, er **87:13**

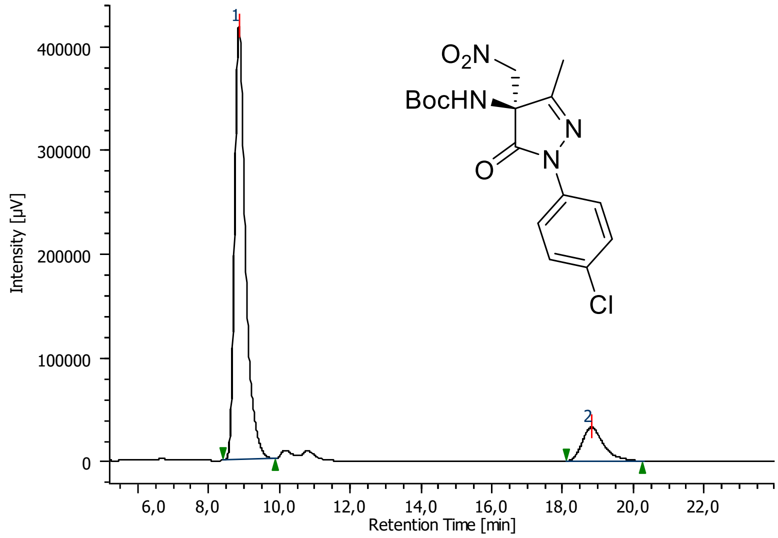

Peak Number	**t_R (min)**	Area (u.a.)	Height (µV)	**Area (%)**	Symmetry Factor
1	**8,858**	8800880	416783	**86,710**	1,516
2	**18,792**	1348925	32062	**13,290**	1,395

Figure S38. HPLC chromatograms of **2i**

tert-Butyl (S)-(1-(3-chlorophenyl)-3-methyl-4-(nitromethyl)-5-oxo-4,5-dihydro-1H-pyrazol-4-yl) carbamate (2j).

Racemic compound

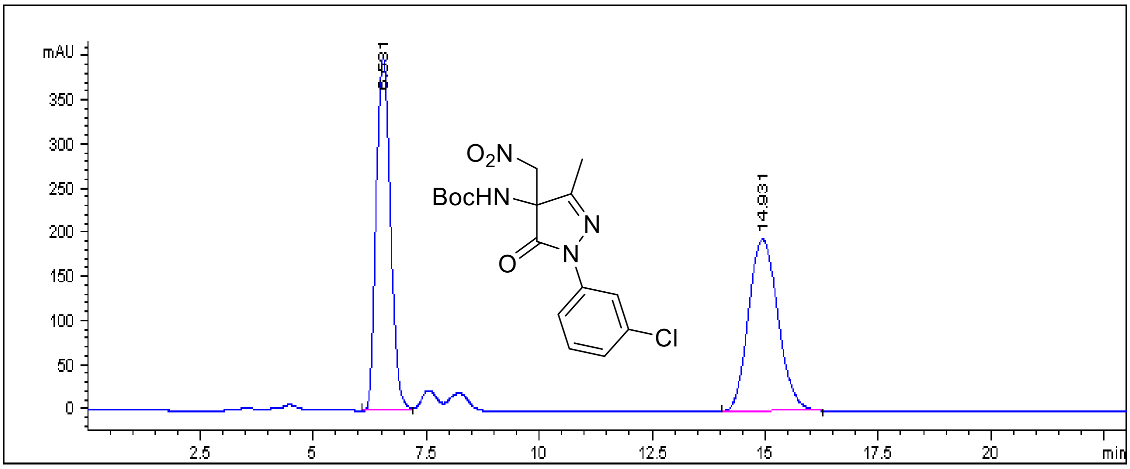

Peak Number	**t_R (min)**	Area (u.a.)	Height (µV)	**Area (%)**	Symmetry Factor
1	**6.531**	8856.6	400.5	**49.856**	0.855
2	**14.931**	8907.8	195.6	**50.144**	0.861

HPLC profile for **2j** compound, er **83:17**

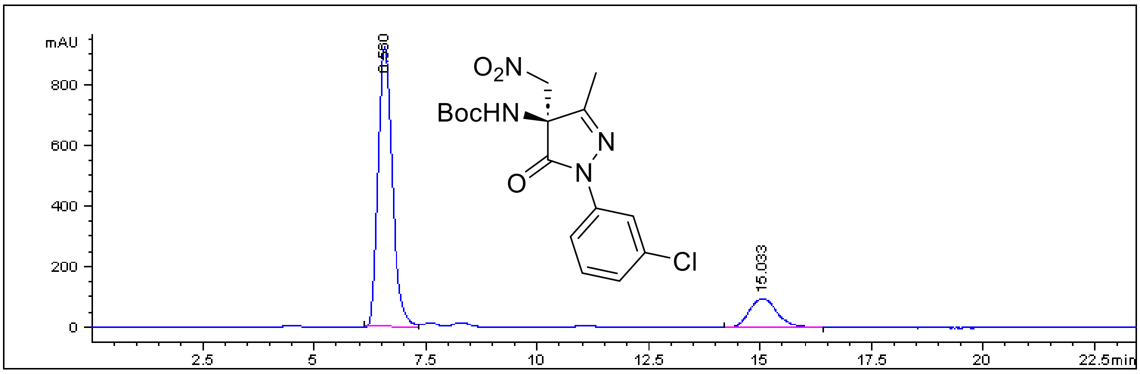

Peak Number	**t_R (min)**	Area (u.a.)	Height (µV)	**Area (%)**	Symmetry Factor
1	**6.56**	20721.6	924.8	**82.935**	0.787
2	**15.033**	4263.8	96	**17.065**	0.835

Figure S39. HPLC chromatograms of **2j**

tert-Butyl (S)-(1-(2-chlorophenyl)-3-methyl-4-(nitromethyl)-5-oxo-4,5-dihydro-1H-pyrazol-4-yl) carbamate (2k).

Racemic compound

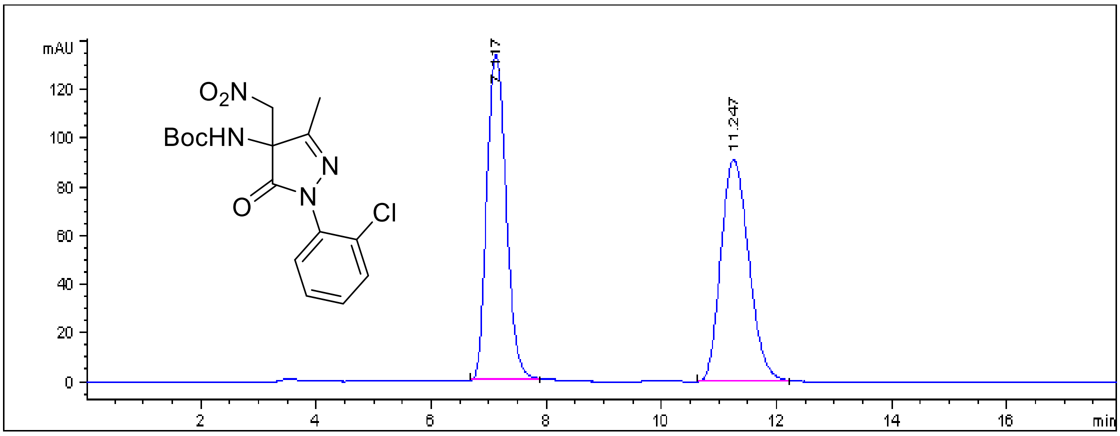

Peak Number	**t_R (min)**	Area (u.a.)	Height (μV)	**Area (%)**	Symmetry Factor
1	**7.117**	3127.1	133.9	**50.348**	0.845
2	**11.247**	3083.9	91.2	**49.652**	0.803

HPLC profile for **2k** compound, er **78:22**

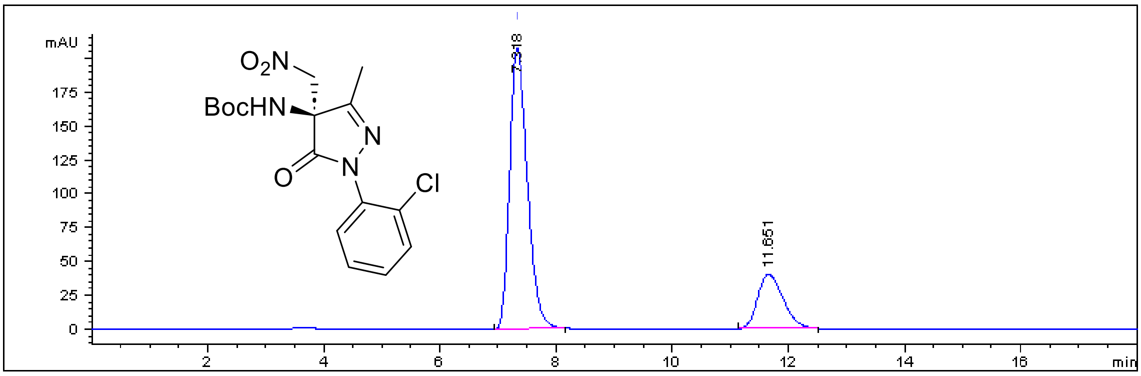

Peak Number	**t_R (min)**	Area (u.a.)	Height (μV)	**Area (%)**	Symmetry Factor
1	**7.318**	4429.3	208.3	**78.060**	0.686
2	**11.651**	1244.9	40.5	**21.940**	0.719

Figure S40. HPLC chromatograms of **2k**

tert-Butyl (S)-(1-benzyl-3-methyl-4-(nitromethyl)-5-oxo-4,5-dihydro-1H-pyrazol-4-yl)carbamate (2l).

Racemic compound

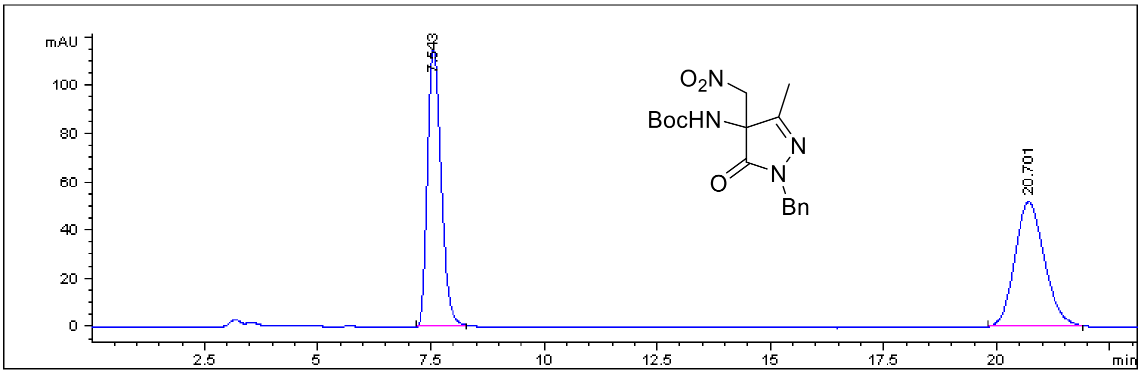

Peak Number	t_R (min)	Area (u.a.)	Height (µV)	Area (%)	Symmetry Factor
1	**7.543**	2381.7	116	**50.218**	0.717
2	**20.701**	2361	52	**49.782**	0.872

HPLC profile for **2l** compound, er **79:21**

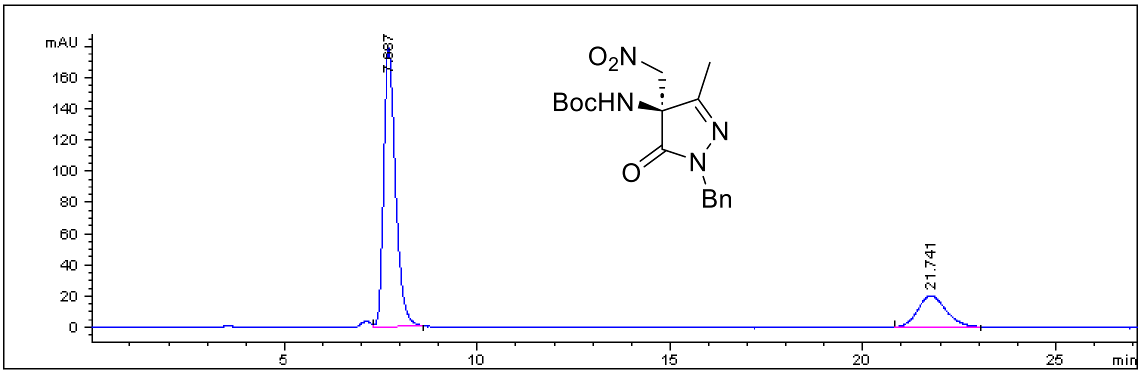

Peak Number	t_R (min)	Area (u.a.)	Height (µV)	Area (%)	Symmetry Factor
1	**7.687**	3896.5	179.3	**78.666**	0.673
2	**21.741**	1056.7	20.4	**21.334**	0.838

Figure S41. HPLC chromatograms of **2l**

tert-Butyl (S)-(1,3-dimethyl-4-(nitromethyl)-5-oxo-4,5-dihydro-1H-pyrazol-4-yl)carbamate (2m).

Racemic compound

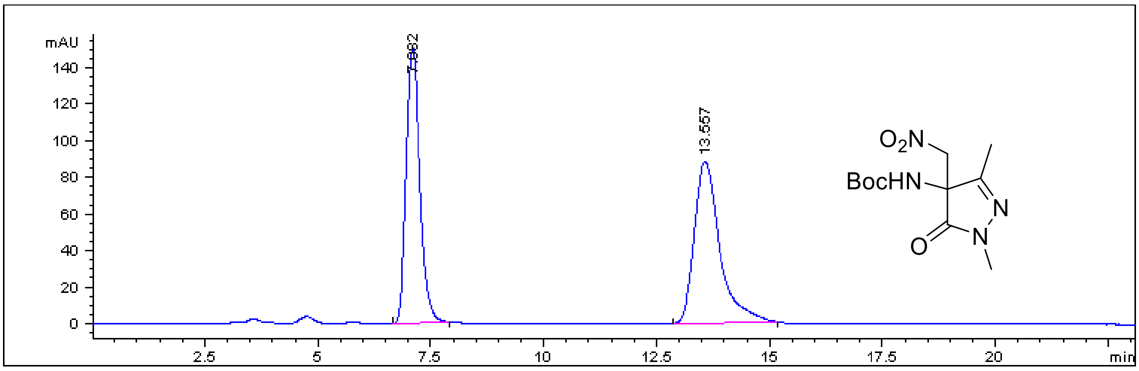

Peak Number	**t_R (min)**	Area (u.a.)	Height (μV)	**Area (%)**	Symmetry Factor
1	**7.082**	3239.3	151.1	**47.861**	0.820
2	**13.557**	3528.9	88.8	**52.139**	0.661

HPLC profile for **2m** compound, er **80:20**

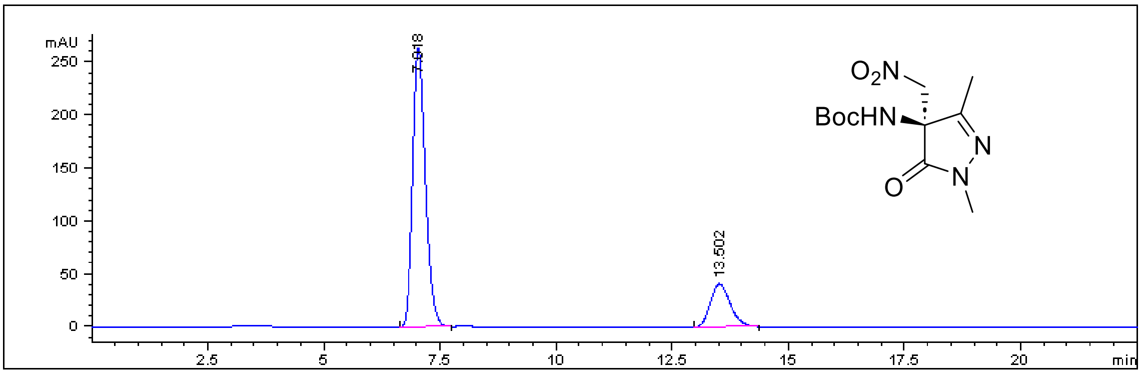

Peak Number	**t_R (min)**	Area (u.a.)	Height (μV)	**Area (%)**	Symmetry Factor
1	**7.018**	5047	264.5	**80.100**	0.767
2	**13.502**	1253.9	41.4	**19.900**	0.768

Figure S42. HPLC chromatograms of **2m**

tert-Butyl (S)-(3-isopropyl-1-methyl-4-(nitromethyl)-5-oxo-4,5-dihydro-1H-pyrazol-4-yl)carbamate (2n).

Racemic compound

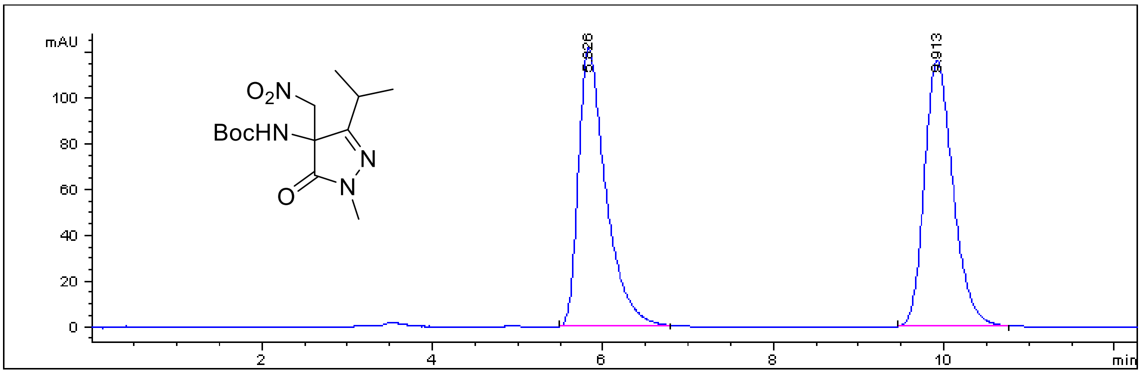

Peak Number	**t$_R$ (min)**	Area (u.a.)	Height (µV)	**Area (%)**	Symmetry Factor
1	**5.826**	2727.2	122.3	**49.975**	0.550
2	**9.913**	2729.9	116.3	**50.025**	0.746

HPLC profile for **2n** compound, er **77:23**

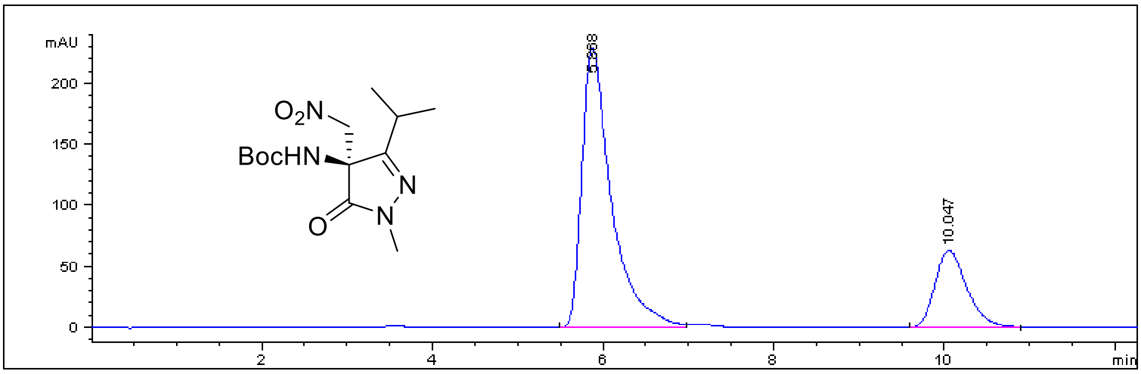

Peak Number	**t$_R$ (min)**	Area (u.a.)	Height (µV)	**Area (%)**	Symmetry Factor
1	**5.868**	5553.6	230	**77.146**	0.494
2	**10.047**	1645.2	63.1	**22.854**	0.725

Figure S43. HPLC chromatograms of **2n**

tert-butyl ((S)-3-methyl-4-((S)-1-nitroethyl)-5-oxo-1-phenyl-4,5-dihydro-1H-pyrazol-4-yl)carbamate (4a)

Racemic compound

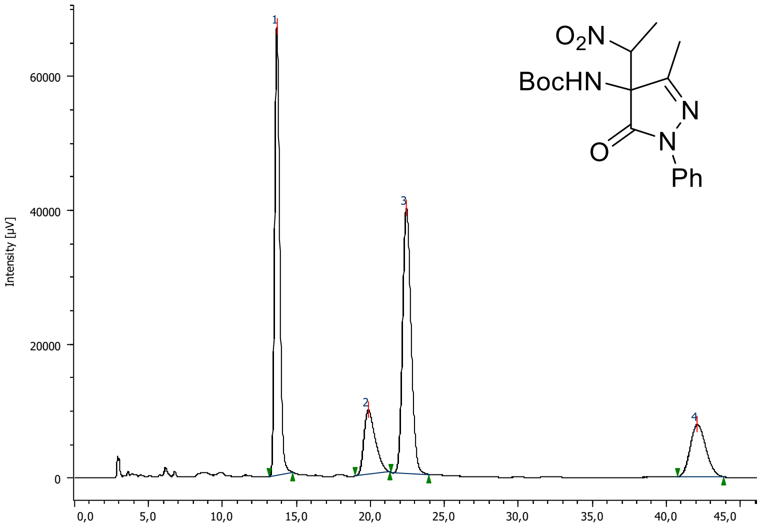

Peak Number	**t_R (min)**	Area (u.a.)	Height (µV)	**Area (%)**	Symmetry Factor
1	**13,667**	1575714	66871	**37,205**	1,189
2	**19,833**	514386	9659	**12,146**	1,474
3	**22,408**	1571479	39600	**37,105**	1,153
4	**42,050**	573611	7808	**13,544**	1,157

HPLC profile for **4a** compound, er: **86:14$_{major}$, 85:15$_{minor}$**

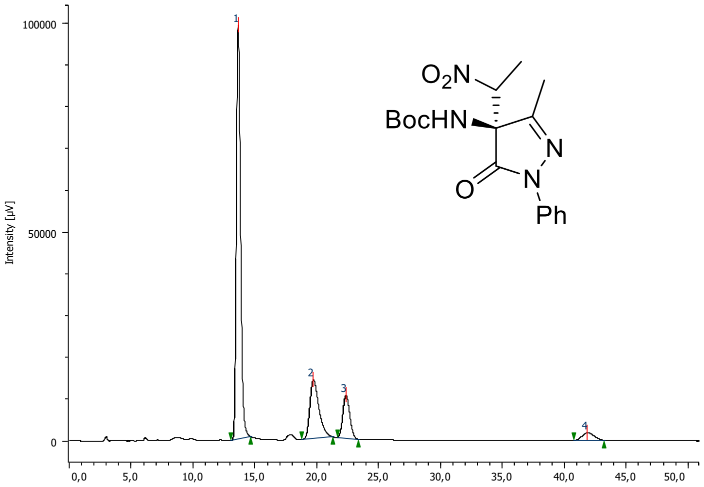

Peak Number	**t_R (min)**	Area (u.a.)	Height (µV)	**Area (%)**	Symmetry Factor
1	**13,633**	2370160	98883	**65,715**	1,238
2	**19,708**	717194	14120	**19,885**	1,479
3	**22,333**	394850	10247	**10,948**	1,151
4	**41,800**	124543	1809	**3,453**	1,136

Figure S44. HPLC chromatograms of **4a**

(S)-4-Amino-5-methyl-4-(nitromethyl)-2-phenyl-2,4-dihydro-3H-pyrazol-3-one (5a).

Racemic compound

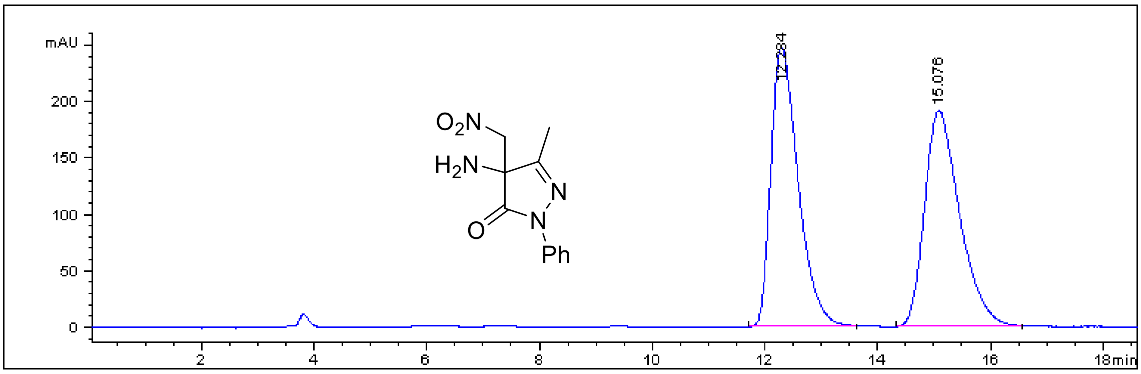

Peak Number	**t$_R$ (min)**	Area (u.a.)	Height (μV)	**Area (%)**	Symmetry Factor
1	**12.284**	8307.8	247.5	**50.086**	0.596
2	**15.076**	8279.3	191.3	**49.914**	0.653

HPLC profile for **5a** compound, er **98:2**

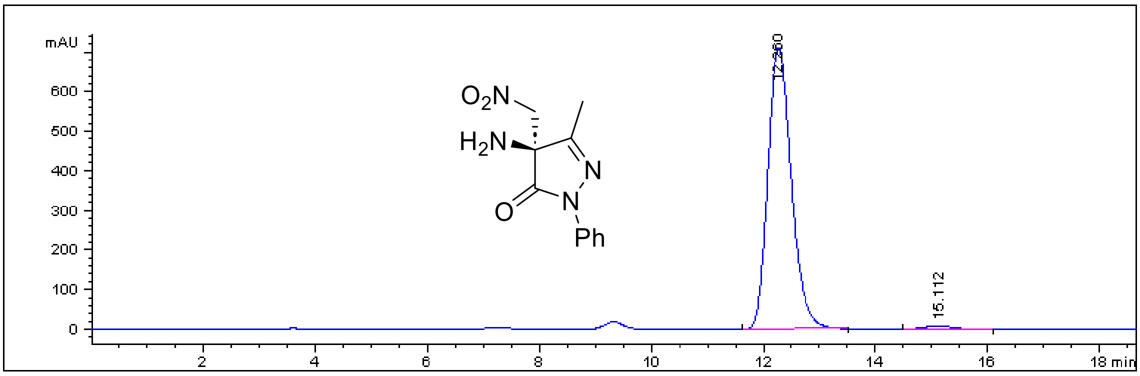

Peak Number	**t$_R$ (min)**	Area (u.a.)	Height (μV)	**Area (%)**	Symmetry Factor
1	**12.26**	20802.3	711.9	**98.445**	0.798
2	**15.112**	328.6	8,3	**1.555**	0.948

Figure S45. HPLC chromatograms of **5a**

(S)-1-(3-Methyl-4-(nitromethyl)-5-oxo-1-phenyl-4,5-dihydro-1H-pyrazol-4-yl)-3-phenylurea (7a).

Racemic compound

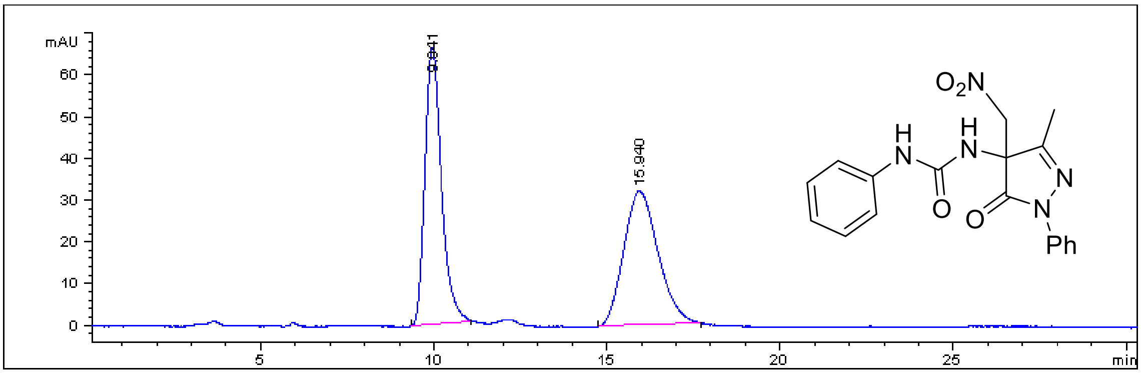

Peak Number	**t_R (min)**	Area (u.a.)	Height (µV)	**Area (%)**	Symmetry Factor
1	**9.941**	2218	66.7	**49.646**	0.73
2	**15.94**	2249.6	32.2	**50.354**	0.841

HPLC profile for **7a** compound, er **98:2**

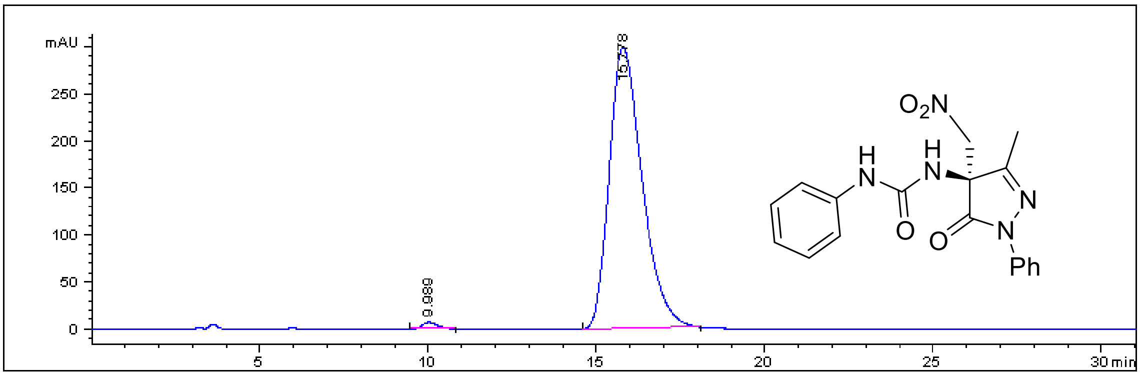

Peak Number	**t_R (min)**	Area (u.a.)	Height (µV)	**Area (%)**	Symmetry Factor
1	**9.989**	240.2	7.1	**1.159**	0.636
2	**15.778**	20484.2	298.8	**98.841**	0.694

Figure S46. HPLC chromatograms of **7a**

(S)-1-(4-bromophenyl)-3-(3-methyl-4-(nitromethyl)-5-oxo-1-phenyl-4,5-dihydro-1H-pyrazol-4-yl)urea (8a).

Racemic compound

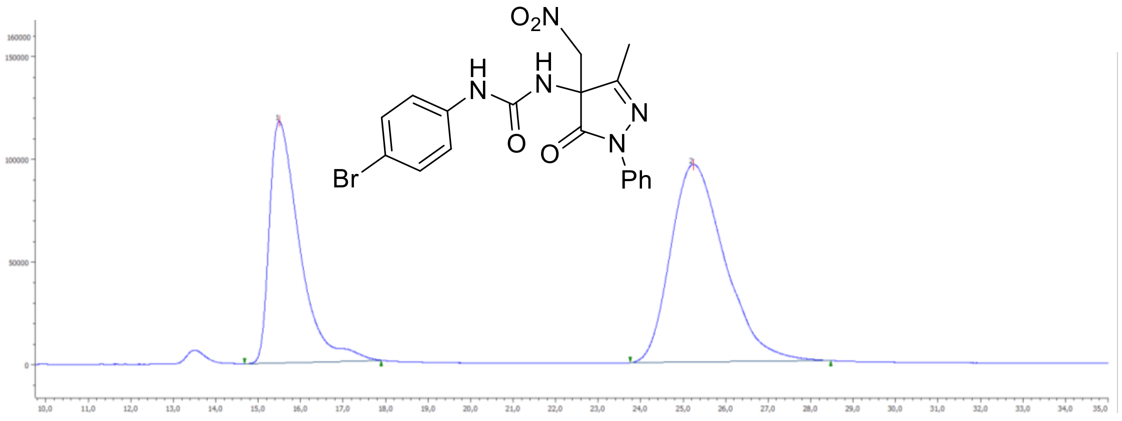

Peak Number	**t_R (min)**	Area (u.a.)	Height (µV)	**Area (%)**	Symmetry Factor
1	**15.493**	5803399	117446	**40.519**	2.216
2	**25.233**	8519381	96192	**59.481**	1.359

HPLC profile for **8a** compound, er **97:3**

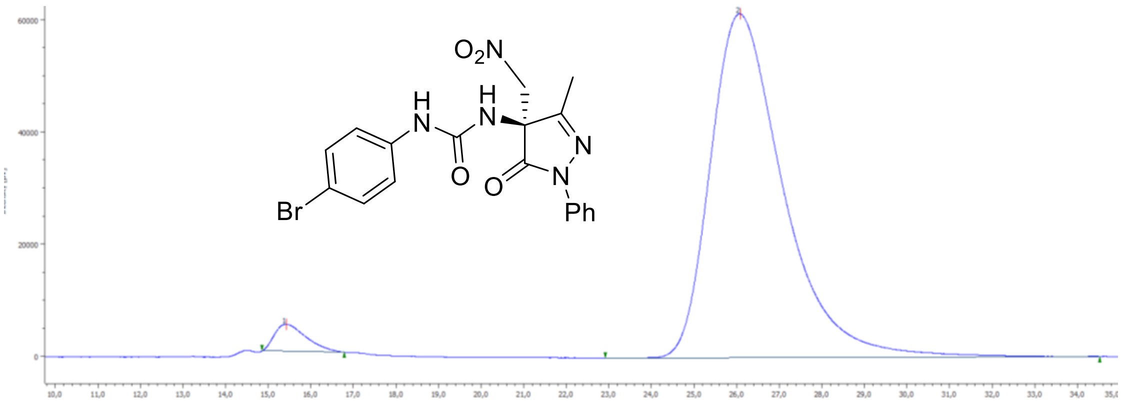

Peak Number	**t_R (min)**	Area (u.a.)	Height (µV)	**Area (%)**	Symmetry Factor
1	**15.420**	249665	4824	**3.320**	1.604
2	**26.070**	7270577	61135	**96.680**	1.481

Figure S47. HPLC chromatograms of **8a**

(S)-N-(3-Methyl-4-(nitromethyl)-5-oxo-1-phenyl-4,5-dihydro-1H-pyrazol-4-yl)acetamide (10a).

Racemic compound

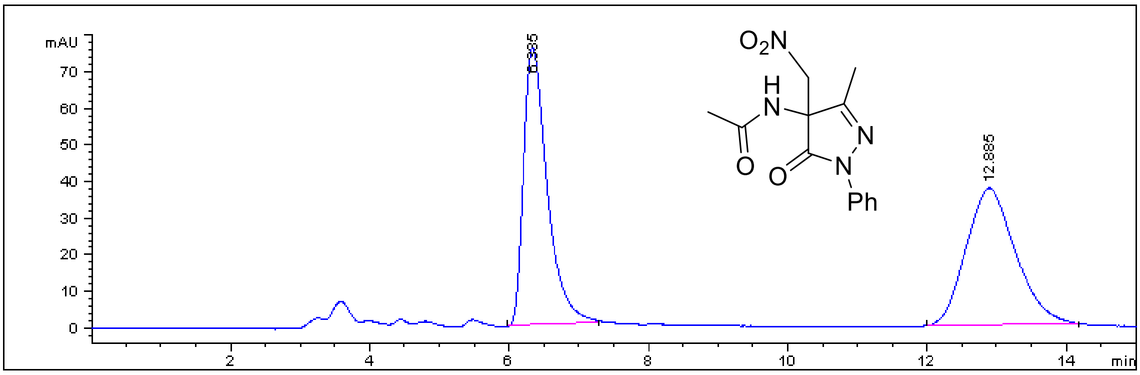

Peak Number	**t_R (min)**	Area (u.a.)	Height (μV)	**Area (%)**	Symmetry Factor
1	**6.335**	1803.5	76	**49.183**	0.589
2	**12.885**	1863.4	37.6	**50.817**	0.855

HPLC profile for **10a** compound, er **98:2**

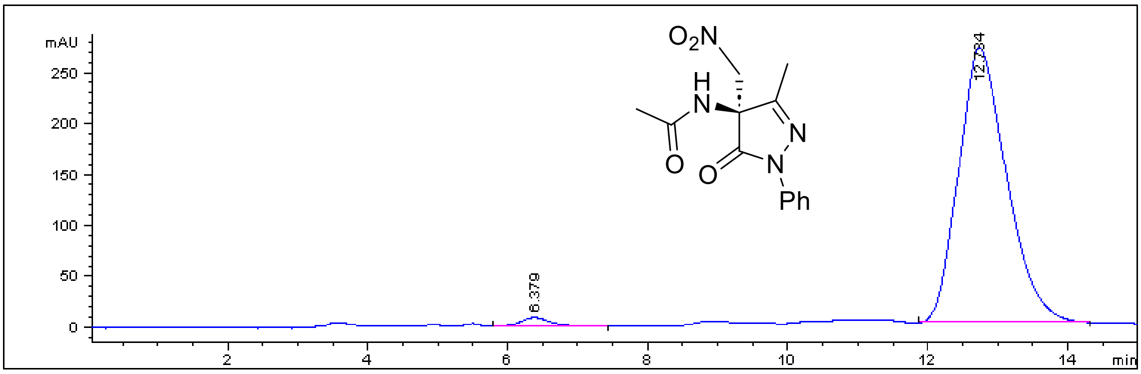

Peak Number	**t_R (min)**	Area (u.a.)	Height (μV)	**Area (%)**	Symmetry Factor
1	**6.379**	243	8.3	**1.845**	0.873
2	**12.734**	12926.9	270.3	**98.155**	0.752

Figure S48. HPLC chromatograms of **10a**
